# An Alternative Finite Difference WENO-Like Scheme with Physical Constraint Preservation for Divergence-Preserving Hyperbolic Systems

**DOI:** 10.1007/s42967-025-00517-y

**Published:** 2025-12-09

**Authors:** Dinshaw S. Balsara, Deepak Bhoriya, Chi-Wang Shu

**Affiliations:** 1 Physics Department, University of Notre Dame, Notre Dame, IN USA; 2 ACMS Department, University of Notre Dame, Notre Dame, IN USA; 3 Division of Applied Mathematics, Brown University, Providence, RI USA

**Keywords:** Hyperbolic PDEs, Divergence free numerical schemes, Physical contraint preservation (PCP), Computational electrodynamics (CED), Finite difference Weighted Essentially Non-Oscillatory (FD-WENO) methods, 35Q85, 35Q75, 35Q35, 35Q61, 35L75, 35L40, 85A30, 65M06, 65M22

## Abstract

Alternative finite difference Weighted Essentially Non-Oscillatory (AFD-WENO) schemes allow us to very efficiently update hyperbolic systems even in complex geometries. Recent innovations in AFD-WENO methods allow us to treat hyperbolic systems with non-conservative products almost as efficiently as conservation laws. However, some PDE systems, like computational electrodynamics (CED), magnetohydrodynamics (MHD), and relativistic magnetohydrodynamics (RMHD), have involution constraints that require divergence-free or divergence-preserving evolution of vector fields. In such situations, a Yee-style collocation of variables proves indispensable, and that collocation is retained in this work. In previous works, only higher order finite volume (FV) discretization of such involution-constrained systems was possible. In this work, we show that substantially more efficient AFD-WENO methods have been extended to encompass divergence-preserving hyperbolic PDEs. Our method retains the Yee-style collocation of normal components of the divergence-free/preserving vector field. However, the variables that require zone-centered evolution are evolved with AFD-WENO methods. Since those variables make up the bulk of the primal variables for the PDE of interest, this results in substantial savings in computational complexity. Even the volumetric reconstruction of the divergence-free/preserving vector field is bypassed. Instead, we realize that any divergence-preserving update of a vector field must have a general form. This general form looks closely like the familiar induction equation that is well known in CED or MHD. We exploit the generality of that form to extract the edge-centered variables that are needed in the update of the facially averaged vector field components. The two-dimensional (2D) Riemann solver is used to provide us with multidimensionally stabilized versions of these update terms. The generality of our approach is demonstrated by the fact that problems in CED, MHD, and RMHD can all be solved by the same general AFD-WENO algorithm that is presented here. Spatial accuracies up to the ninth order of accuracy are demonstrated. Several stringent test problems from CED, MHD, and RMHD are shown. We also show that the algorithm takes well to the physical constraint-preserving (PCP) formulation of AFD-WENO schemes that was presented by the authors. The efficient and time-explicit PCP strategy for divergence-preserving PDEs that we have presented here extends the applicability of our method to very stringent MHD and RMHD problems.

## Introduction

**Fig. 1:**
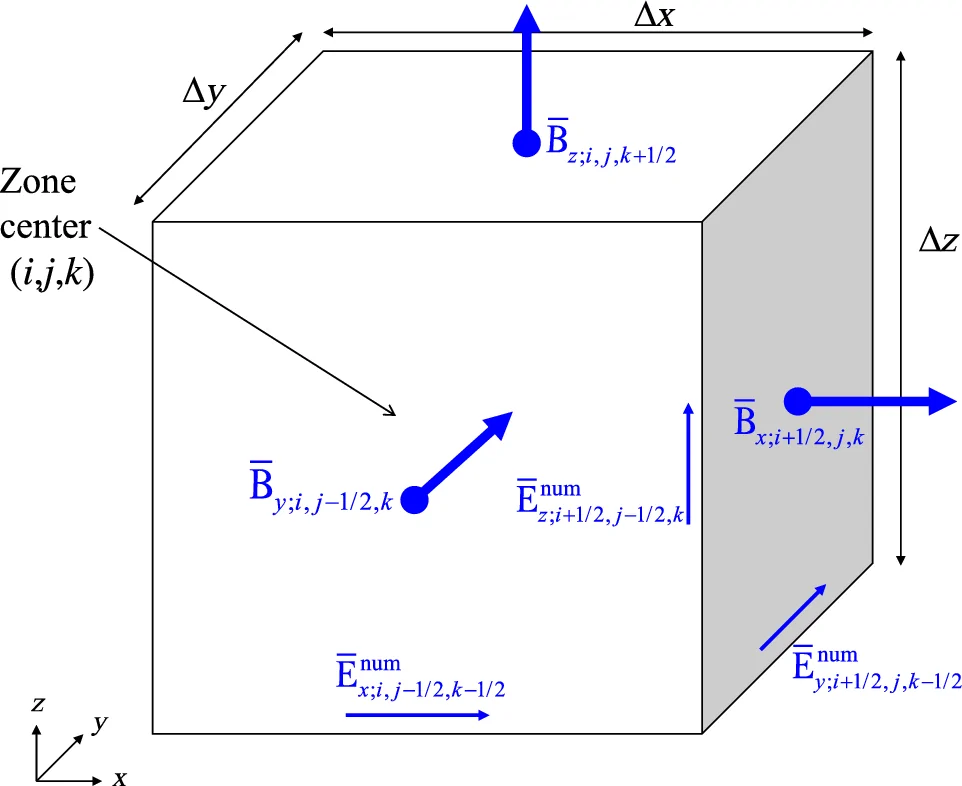
Primal variables of the scheme, given by the normal components of the magnetic induction, are facially collocated. The components of the primal magnetic field vector are shown by the thick blue arrows. The overbars on the magnetic field components indicate that these are facially averaged. They undergo an update from the induction equation. The edge-collocated electric fields, which are used for updating the facial magnetic induction components, are shown by the thin blue arrows close to the appropriate edge. They too have overbars to indicate that they are edge-averaged. The superscript “num” for the electric field components indicates that they are multidimensionally stabilized and, therefore, suitable for use in a numerical scheme

Novel applications in science and engineering have called for higher order schemes for the simulation of hyperbolic PDEs. Most of these PDEs are strongly non-linear which implies that it is beneficial to pick solution methods that are generally drawn from the family of higher order Godunov schemes. Within that general family of Godunov schemes, the two most popular strategies for achieving higher order accuracy consist of Weighted Essentially Non-Oscillatory (WENO) schemes and Discontinuous Galerkin (DG) schemes. Essentially Non-Oscillatory methods were developed in finite volume (FV) form by Harten et al. [56]. DG schemes were also subsequently developed (Cockburn and Shu [48, 49], Cockburn et al. [46, 47]). Early work on this topic focused on conservation laws. Strong Stability Preserving Runge-Kutta (SSP-RK) methods for high-order time-integration (Shu and Osher [77], Spiteri and Ruuth [80, 81]) were developed to match the high-order spatial accuracy. Yet, it quickly became apparent that practitioners wanted methods that could handle stiff source terms (Pareschi and Russo [71], Kupka et al. [66]) and non-conservative products (Baer and Nunziato [6], Andrianov and Warnecke [4], Castro et al. [44]). Newer classes of hyperbolic PDEs, such as magnetohydrodynamics (MHD) and computational electrodynamics (CED), came with their own involution constraints. For example, both MHD and CED require the ability to evolve vector fields in a divergence-free or divergence-preserving fashion. The divergence constraint is more of a geometrical constraint, with the result that a collocation of primal variables and update variables that is consistent with a Yee [87] type mesh becomes essential. Figure 1 shows how the induction equation, which is common to MHD and CED, is discretized. The components of the magnetic fields are collocated, in facially averaged form at the faces of the mesh; as in Fig. 1. These are updated using edge-averaged electric field components at the edges of the mesh; as in Fig. 1. The induction equation for MHD is generically written as

1 $$\frac{{\partial {\mathbf{B}}}}{\partial t} + \nabla \times {\mathbf{E}} = 0$$

with ${\mathbf{E}} = - {\mathbf{v}} \times {\mathbf{B}}$, where “${\mathbf{v}}$” is the fluid velocity. In Fig. 1, we see that the components of the magnetic field “${\mathbf{B}}$” (which are the primal variables of the scheme) are collocated at the faces of the mesh and the discrete divergence constraint is only preserved if the electric fields “${\mathbf{E}}$” (which provide the update) are collocated at the edges of the mesh. As a result, new styles of discretization and multidimensional upwinding had to be invented. This paper focuses on hyperbolic PDE systems that may indeed have a dominant zone-centered collocation of variables; however, the PDE system includes one or more vector fields that look like the update of the induction equation. Past efforts have focused on individual PDEs. The *overarching goal* of this paper is to discern the common structure of all PDE systems that have vector fields that have an update that looks like the induction equation, and then to develop common methods for the numerical evolution of such systems.

MHD was indeed the first involution-constrained system that was extended to include higher order Godunov methodology while preserving the divergence-free evolution of magnetic fields. Early work on divergence-free Godunov schemes for MHD was restricted to second order (Dai and Woodward [51], Ryu et al. [73], Balsara and Spicer [36]). The need to do adaptive mesh refinement (AMR) at second order forced one to pay attention to the TVD-based divergence-free reconstruction of vector fields (Balsara [8]). FV-WENO-based, divergence-free reconstruction, as well as divergence-free AMR, has been extended to higher orders (Balsara [10, 11], Balsara et al. [33], Balsara and Sarris [34]). This vector field reconstruction ensures that if the modes of variation within each face of Fig. 1 are known for the magnetic field components, then the entire divergence-free vector field can be reconstructed at all locations within the volume shown in Fig. 1. However, note too from Fig. 1 that the edge-centered electric fields are also needed. In keeping with the realization that upwinding provides stabilization, we realize that the edge-collocated electric fields shown in Fig. 1 will have to be upwinded in their two transverse directions. This insight led to the development of multidimensional Riemann solvers (Balsara [12, 13, 15, 16], Balsara et al. [24], Balsara and Dumbser [23], Balsara and Nkonga [31]). Higher order divergence-free reconstruction along with the multidimensional upwinding from the multidimensional Riemann solver opened the door to globally divergence-free higher order WENO-based MHD schemes (Balsara et al. [14, 30, 32]). FV-WENO-based schemes for CED that used the same two ideas were also invented (Balsara et al. [37, 38]). Balsara and Käppeli [26] formulated globally divergence-free DG schemes for MHD; and working DG schemes for MHD, which used WENO methods for stabilization, were presented in Balsara et al. [29]. Likewise, Balsara and Käppeli [27] formulated globally divergence-preserving DG schemes for CED; and working DG schemes for CED with up to fifth order of accuracy were presented in Hazra et al. [57].

All of the advances in the above paragraphs were reported within the context of FV formulations. However, ever since the seminal papers by Shu and Osher [77, 78], we have been aware that higher order finite difference WENO (FD-WENO) schemes are substantially more efficient compared to FV-WENO schemes. The first FD-WENO schemes for conservation laws that were suitable for production codes emerged in Jiang and Shu [62] and Balsara and Shu [35]. Modern PDEs also include non-conservative products and stiff source terms. In Balsara et al. [18], a way was found for simulating such PDEs with non-conservative products and stiff source terms using FD-WENO schemes. In Balsara et al. [19], we developed Alternative FD-WENO (AFD-WENO) methods that can accommodate non-conservative products and are suitable for production codes. The AFD-WENO schemes offer the following advantages. First, any Riemann solver can be used, which can be useful for several applications where Riemann solvers with special capabilities are desired. Second, the free stream condition can be respected on curvilinear meshes (Jiang et al. [63, 64]). Third, variables that are in the flux conservative form can be treated in a manner that respects conservation, while also accommodating non-conservative products. Fourth, stiff source terms can be included quite easily owing to the pointwise nature of the scheme. Fifth, well-balancing can be easily added to the scheme (Xu and Shu [86]). However, a major deficiency up to now has been the fact that AFD-WENO methods are not available for PDEs that have a globally divergence-free or divergence-preserving involution constraint. The *first important goal* of this paper is to develop schemes that use many of the AFD-WENO principles while including the divergence constraint.

All PDE systems that have a divergence constraint have a structure like the induction equation. Therefore, they will necessarily have to retain the Yee-mesh collocation philosophy shown in Fig. 1. Because the Stokes law has to be applied to the induction equation, it necessarily means that the facial magnetic fields in Fig. 1 are face-averaged and the edge-centered electric fields are edge-averaged in Fig. 1. As a result, we realize that the constrained vector fields will have to retain an FV style of update. However, we realize that the induction equation forms a very small part of much larger PDE systems. The MHD equations (Alfvén [3], Jeffrey and Taniuti [61], Roe and Balsara [72]), or the Chew Goldberger and Low (CGL) equations (Chew et al. [45], Bhoriya et al. [40], Singh et al. [79]), or the equations of Relativistic MHD (RMHD) (Anile [5], Balsara [9], del Zanna et al. [88], Balsara and Kim [28]) are cases in point. In all those PDEs, we have a much larger hydrodynamical set of equations that are coupled via magnetic fields to one single induction equation for the evolution of the magnetic vector field. As a result, it would still be advantageous to treat the rest of the variables with the highly efficient and versatile AFD-WENO formulation while retaining a concept of area-averaged updates for the facial components of the magnetic vector field. This is the compromise position that we will adopt in this paper.

Such compromise positions, which combine some elements of FD methods with some elements of FV methods to obtain greater computational efficiency, have been adopted before. For example, see the WENO-based work of Buchmüller et al. [42] who drew their inspiration from McCorquodale and Colella [68], Shi et al. [76], and Zhang et al. [89]. Recently, Donnert et al. [52] used a fifth-order FD-WENO formulation for zone-centered variables along with a second-order formulation for the magnetic fields to at least get a more accurate evolution of the fluid variables, even though the evolution of the magnetic fields was only second-order accurate. Seo and Ryu [75] improved on that paper by making higher order interpolations of the facial fluxes to get higher order edge-centered electric fields. This yielded a fifth-order FD-WENO formulation that was indeed fifth-order for the flow variables as well as the magnetic field. The above advantages have been made on a case-by-case basis for each different type of the PDE. The *second important goal* of this paper is to show that equations like the induction equation—which give rise to divergence-based involution constraints—all have a common structure. By focusing on that common structure, we find a common solution for the multidimensional upwinding of the induction equation. This common solution, which we develop in this paper, enables us to offer an AFD-WENO-based solution for all PDEs that have a divergence-based involution. The benefit from this innovation is that vast swathes of divergence-constrained PDEs can all be treated using AFD-WENO schemes of progressively higher order using the same algorithmic framework.

It should also be mentioned that WENO reconstruction and interpolation have seen several recent advances which make it easy to implement such ideas. In Henrick et al. [58] and Borges et al. [41], it was shown that accuracy can be retained at critical points by modifying the non-linear WENO weights. In Balsara et al. [25], it was shown that the smoothness indicators for one-dimensional (1D) WENO reconstruction can be written analytically as the sum of perfect squares. In the supplement to Balsara et al. [33], it was shown that the smoothness indicators for two-dimensional (2D) and three-dimensional (3D) WENO reconstruction can also be written analytically as the sum of perfect squares, and the WENO reconstruction was simplified in two and three dimensions. In Balsara et al. [19], 1D WENO interpolation was also presented at very high orders. Several authors (Levy et al. [67], Zhu and Qiu [90], Balsara et al. [25], Cravero and Semplice [50], Semplice et al. [74], Zhu and Shu [91]) have shown the value of using multiple stencils of different orders for stabilizing WENO schemes. These advances in WENO reconstruction and WENO interpolation make it easy to formulate and implement the methods presented here. (This distinction between reconstruction and interpolation is very important. In Balsara et al. [25], several WENO reconstruction formulae at different orders have been provided that are useful for classical FD-WENO. In Balsara et al. [19], analogous pointwise WENO interpolation formulae have been provided at different orders for use in AFD-WENO.)

It is also worth mentioning for the interested reader that there is a very attractive ecosystem of ideas developing around the AFD-WENO methods. Well-balanced AFD-WENO methods for conservation laws have been documented in Xu and Shu [86]. It is also important to have higher order methods that can preserve positivity of density and pressure, and keep other variables within physical bounds. This goes under the rubric of physical constraint-preserving (PCP) methods. PCP methods for AFD-WENO have also been documented in Bhoriya et al. [39]. Other noteworthy work on PCP schemes in this area included papers by Wu and Shu [84, 85]. The methods have also been extended to extremely large hyperbolic systems like the equations of general relativity in Balsara et al. [22]. This illustrates that along with the basic algorithm, there are numerous additional capabilities available for AFD-WENO methods that make them generally useful in a large number of contexts.

The plan of this paper is as follows. In Sect. 2, we motivate the idea that it is possible to mix FD- and FV-WENO approaches to get the best advantages of computational efficiency. In Sect. 3, we discuss the structure of the induction equation and the multidimensional dissipation it requires to achieve multidimensionally upwinded schemes. In Sect. 4, we provide a step-by-step implementation of the scheme. Section 5 describes the additional steps needed for obtaining an efficient and time-explicit PCP algorithm for divergence-preserving PDEs. Section 6 presents the accuracy analysis for several hyperbolic PDEs that have a divergence-based involution constraint. Section 7 presents numerous stringent test problems for the same PDE systems. Section 8 ends with some conclusions.

## Motivating a Mixed, i.e., Finite Difference (FD) and Finite Volume (FV), Form for Divergence-Preserving PDEs

Practically, all users of higher order Godunov schemes would be comfortable with the idea of a higher order FD approximation of a conservation law $\partial_{t} {\mathbf{u}} + \partial_{x} {\mathbf{f}} + \partial_{y} {\mathbf{g}} + \partial_{z} {\mathbf{h}} = 0$. We consider the spatial discretization of this equation on a uniform mesh with zone sizes Δx, Δy, and Δz in the *x*-, *y*-, and *z*-directions, respectively. The multidimensional FD update that is discrete in space but continuous in time looks like

2 $$\partial_{t} {\mathbf{u}}_{i,j,k}^{{}} = - \frac{1}{\Delta x}\left( {{\mathbf{f}}_{i + 1/2,j,k}^{{}} - {\mathbf{f}}_{i - 1/2,j,k}^{{}} } \right) - \frac{1}{\Delta y}\left( {{\mathbf{g}}_{i,j + 1/2,k}^{{}} - {\mathbf{g}}_{i,j - 1/2,k}^{{}} } \right) - \frac{1}{\Delta z}\left( {{\mathbf{h}}_{i,j,k + 1/2}^{{}} - {\mathbf{h}}_{i,j,k - 1/2}^{{}} } \right).$$

The above update term can then be combined with an SSP-RK update to achieve higher order accuracy in time. Although the variables in the above equation do not have the same meaning/interpretation as they would have in an FV approximation, we understand that the flux does, nevertheless, satisfy a telescoping property with the result that a notion of conservation can still be asserted. AFD-WENO schemes do have the selling point that they offer high accuracy along with high processing speed for multidimensional problems. Consequently, we begin by claiming that we would like to have ultra-efficient mimetic AFD-WENO schemes for involution-constrained PDEs.

Here, we will devise AFD-WENO-like schemes that update zone-centered variables as point values. However, it proves very valuable to retain the integral sense in which Eq. (1) holds. In other words, we want the discrete in space, but continuous in time, version of Eq. (1) to be given by the FV-style approximation

3 $$\partial_{t} {\overline{\mathrm{B}}}_{x; \, i + 1/2,j,k} { = } - \frac{1}{\Delta y\Delta z}\left( {\Delta z{\overline{\mathrm{E}}}_{z; \, i + 1/2,j + 1/2,k}^{{{\mathrm{num}}}} - \Delta z{\overline{\mathrm{E}}}_{z; \, i + 1/2,j - 1/2,k}^{{{\mathrm{num}}}} + \Delta y{\overline{\mathrm{E}}}_{y; \, i + 1/2,j,k - 1/2}^{{{\mathrm{num}}}} - \Delta y{\overline{\mathrm{E}}}_{y; \, i + 1/2,j,k + 1/2}^{{{\mathrm{num}}}} } \right);$$

4 $$\partial_{t} {\overline{\mathrm{B}}}_{y; \, i,j - 1/2,k} { = } - \frac{1}{\Delta x\Delta z}\left( {\Delta x{\overline{\mathrm{E}}}_{x; \, i,j - 1/2,k + 1/2}^{{{\mathrm{num}}}} - \Delta x{\overline{\mathrm{E}}}_{x; \, i,j - 1/2,k - 1/2}^{{{\mathrm{num}}}} + \Delta z{\overline{\mathrm{E}}}_{z; \, i - 1/2,j - 1/2,k}^{{{\mathrm{num}}}} - \Delta z{\overline{\mathrm{E}}}_{z; \, i + 1/2,j - 1/2,k}^\mathrm{num} } \right);$$

5 $$\partial_{t} {\overline{\mathrm{B}}}_{z; \, i,j,k + 1/2} { = } - \frac{1}{\Delta x\Delta y}\left( {\Delta x{\overline{\mathrm{E}}}_{x; \, i,j - 1/2,k + 1/2}^{{{\mathrm{num}}}} - \Delta x{\overline{\mathrm{E}}}_{x; \, i,j + 1/2,k + 1/2}^{{{\mathrm{num}}}} + \Delta y{\overline{\mathrm{E}}}_{y; \, i + 1/2,j,k + 1/2}^{{{\mathrm{num}}}} - \Delta y{\overline{\mathrm{E}}}_{y; \, i - 1/2,j,k + 1/2}^{{{\mathrm{num}}}} } \right).$$

The magnetic field components with overbars, ${\overline{\mathrm{B}}}_{x}$, ${\overline{\mathrm{B}}}_{y}$, and ${\overline{\mathrm{B}}}_{z}$ in the formulae above, are area averages over the faces of the mesh; see Fig. 1. The numerically stabilized electric field components with overbars, ${\overline{\mathrm{E}}}_{x}^{{{\mathrm{num}}}}$, ${\overline{\mathrm{E}}}_{y}^{{{\mathrm{num}}}}$, and ${\overline{\mathrm{E}}}_{z}^{{{\mathrm{num}}}}$ in the formulae above, are high-order accurate line averages along the edges of the mesh; see Fig. 1. The overbars in the above three equations are intended to highlight this averaging. Please realize that without this integral interpretation, Stokes’ law will not work. The divergence constraint on the magnetic field will, therefore, be exactly preserved by the above three equations in an integral sense.

We realize, therefore, that Eq. (2) will be satisfied in the sense of point values. Usually, most PDE systems will have a large number of zone-centered conserved variables. Furthermore, these PDEs need to satisfy a free stream condition even when curvilinear logically Cartesian meshes are used. For that reason, AFD-WENO is an optimal solution choice for Eq. (2). This is especially true if stiff sources are also involved; see Balsara et al. [18–20]. Therefore, our choice of AFD-WENO will keep the computational cost down to a minimum for the part of the calculation that is computationally very costly. On the other hand, it is rare to have multiple equation sets of the form shown in Eq. (1). Typically, most PDEs of interest have only one divergence-preserving vector field. The only counter-example that we know of is Maxwell’s equations, which have two such sets. We do not seek a multidimensional constraint-preserving reconstruction for the divergence-preserving vector field, because that could increase the cost if it is not coupled to an efficient ADER predictor step. Instead, we choose a nominal FV-style discretization for Eq. (1), but we do this using the full set of FD tricks at our disposal, thereby reducing the computational cost for evolving all types of divergence-preserving PDEs. Thus, our solution strategy will be a hybrid: we will use AFD-WENO methods for conservation law-like structures in the solution vector, but we will also nominally use an FV approach for the evolution of the divergence-preserving parts of the PDE.

## Understanding the Structure of the Multidimensional Dissipation in Divergence-Preserving PDEs

From the discussion in Sect. 1, and from Fig. 1, we have seen that the electric field components (that are parallel to the edges of the mesh) have to be stabilized in the two directions that are transverse to the field (and indeed transverse to the edges of the mesh). This calls for a 2D Riemann solver to give us a numerically stabilized value for the electric field components along each edge. Here, we focus on the *z*-component of the electric field that lies along the *z*-edges of the mesh; the analogous expressions for the *x*- and *y*-edges can be obtained by cyclic rotations.

In this section, we derive the explicit expressions of the numerically stabilized edge-aligned electric field in the LLF and HLL limits. The HLL limit is very useful, because it ensures that we have a good supersonic limit for the electric field that we get from the multidimensional Riemann solver. Our practical experience has been that for stringent MHD test problems, it is sometimes beneficial to have a well-designed supersonic limit in the Riemann solver. This is true both for the 1D and 2D Riemann solvers. The section is divided into three sub-sections. In Sect. 3.1, we do some stage-setting. In Sect. 3.2, we derive the edge-aligned electric field for the 2D LLF Riemann solver. In Sect. 3.3, we derive the edge-aligned electric field for the 2D HLL Riemann solver. Strategies for endowing the multidimensional HLL Riemann solver with internal sub-structure have also been described in Balsara and Nkonga [31], so we do not need to describe that here.

### Stage-Setting for the Evaluation of the Edge-Centered Electric Field

We focus on blending AFD-WENO with Eqs. (3), (4), and (5) in the text. The AFD-WENO algorithm already requires us to evaluate the fluxes as point values at each zone center. To that end, it is worth noting that the electric fields are also available from the fluxes, which are evaluated at the zone centers. Therefore, the electric field components can be interpolated with higher order accuracy and those higher order interpolants can be evaluated at the edges of the mesh. However, note that these zone-centered point values for the electric fields do not have any contribution from the numerical dissipation that is needed to stabilize the scheme. Therefore, to efficiently evaluate the numerically stabilized electric field at the edges of the mesh, we have to make two innovations. First, we have to efficiently interpolate the dissipation-free zone-centered electric field components with high accuracy to the appropriate edges of the mesh. Second, we have to find a way to add numerical dissipation in a way that is highly accurate and proportional only to the jumps in the reconstructed magnetic field components at the edges of the mesh. Both these innovations are needed to stabilize the update in Eq. (1).

Let us illustrate the above two innovations by briefly focusing on the edge-collocated *z*-component of the electric field in Fig. 1. It has four zones surrounding it, so we will have four *z*-components of the electric field at those four zone centers. We will need to interpolate those *z*-components of the electric field with high accuracy to the center of the zone edge shown in Fig. 1. (A strategy for efficiently turning point values into line integrals will be described later.) This is accomplished via a 2D WENO interpolation in the *xy*-plane and is, therefore, quite inexpensive. We will soon show that for all equations that are formally similar to Eq. (1), the numerical dissipation is governed entirely by the jumps in the facially reconstructed *x*- and *y*-components of the magnetic field at the *z*-edge being considered in Fig. 1. Notice that the *x*-component of the facial magnetic field in Fig. 1 only needs to be reconstructed two-dimensionally from the neighboring faces in the *yz*-directions, whereas the *y*-component of the facial magnetic field in Fig. 1 only needs to be reconstructed two-dimensionally from the neighboring faces in the *xz*-directions. These are also inexpensive 2D WENO reconstructions and the result of those reconstructions can be re-used at other neighboring edges. Therefore, this step is also very inexpensive. We pay attention to the very important topic of multidimensional dissipation for all equations that are formally similar to Eq. (1) in this section.

Equation (1) can be written in the flux form as

6 $$\frac{\partial }{\partial t}\left( {\begin{array}{*{20}c} {B_{x} } \\ {B_{y} } \\ {B_{z} } \\ \end{array} } \right) + \frac{\partial }{\partial x}\left( {\begin{array}{*{20}c} 0 \\ { - E_{z} } \\ {E_{y} } \\ \end{array} } \right) + \frac{\partial }{\partial y}\left( {\begin{array}{*{20}c} {E_{z} } \\ 0 \\ { - E_{x} } \\ \end{array} } \right) + \frac{\partial }{\partial z}\left( {\begin{array}{*{20}c} { - E_{y} } \\ {E_{x} } \\ 0 \\ \end{array} } \right) = 0.$$

In general, Eq. (6) will be a sub-portion of a larger PDE system. The fluxes in Eq. (6) always have a very special anti-symmetrical form. Notice from Eq. (6) that the second component of the *x*-flux is just the negative of the first component of the *y*-flux; the third component of the *y*-flux is just the negative of the second component of the *z*-flux; likewise, the first component of the *z*-flux is just the negative of the third component of the *x*-flux. At a deeper level, these anti-symmetries are an inevitable consequence of the tensorial invariance of Maxwell’s equations, but we do not delve into that idea any further here. This form of the fluxes in Eq. (6) is very central to update equations that have the structure shown in Eq. (1). Therefore, we do not posit any constitutive relationship between the vector field “**E**” and the vector field “**B**”. (For example, in MHD, we have the constitutive relation ${\mathbf{E}} = - {\mathbf{v}} \times {\mathbf{B}}$, with “**v**” as the velocity vector of the fluid. However, for the purposes of the argument developed in this section, we do not use this fact.) Even without asserting a constitutive relationship, our intention is to show that the very structure of Eq. (1) naturally gives us a very special structure for the multidimensional Riemann solver. The anti-symmetry that is central to Eq. (1) gives rise to the dualism that was originally exploited in numerical schemes (Balsara and Spicer [36]). Furthermore, we wish to show that the structure is such as to give us a centered average term along with a very general structure of the dissipation term. (Recall that the 1D LLF Riemann solver can also be written as a centered average flux and a dissipation term. Recall too that this convenient split was then put to good use as a building block for a classical FD-WENO scheme.) In a similar fashion, we wish to use the multidimensional Riemann solver of Balsara [12, 13, 15] to write the electric field at the edges as a centered average term along with a multidimensional dissipation term.

**Fig. 2:**
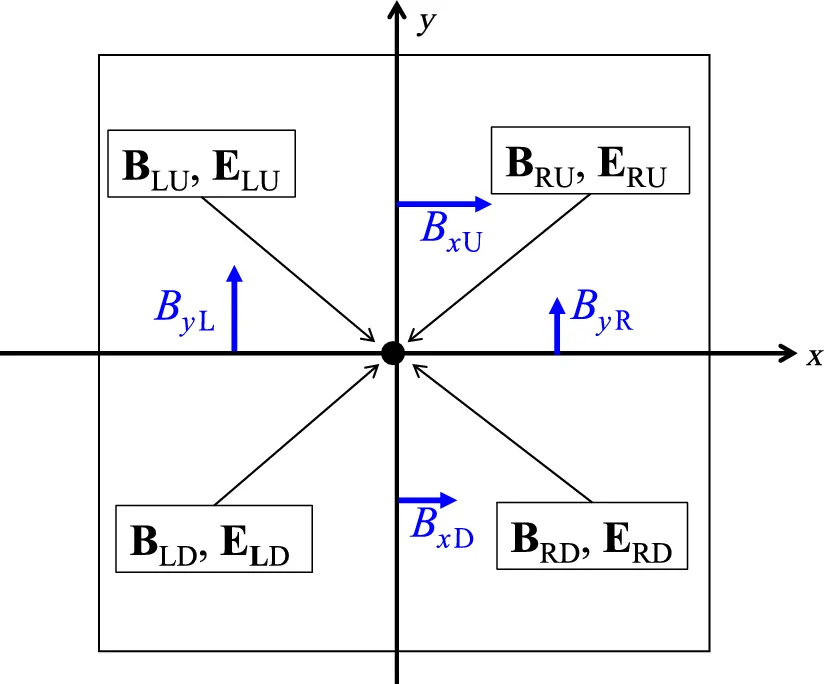
Four zones in the *xy*-plane that come together at the *z*-edge of a 3D mesh. Since the mesh is viewed from the top in-plane view, the *z*-edge is shown by the black dot, and the four abutting zones are shown as four squares. The four incoming states have subscripts given by “RU” for right-upper; “LU” for left-upper; “LD” for left-down; “RD” for right-down. Figure 2 shows the situation before the states start interacting via four 1D and one multidimensional Riemann problems. The black arrows indicate that higher order 2D interpolation can eventually be used to obtain the centered part of the electric field at the *z*-edge. The blue arrows denote the normal components of the magnetic field at the zone faces. Owing to the constraint, these field components (blue arrows) are continuous across zone faces. They can, therefore, be obtained from the higher order facial reconstruction of the normal component of the magnetic field within each face. This provides higher order values of the *x*- and *y*-components of the magnetic field at the *z*-edge that minimize the dissipation terms

**Fig. 3:**
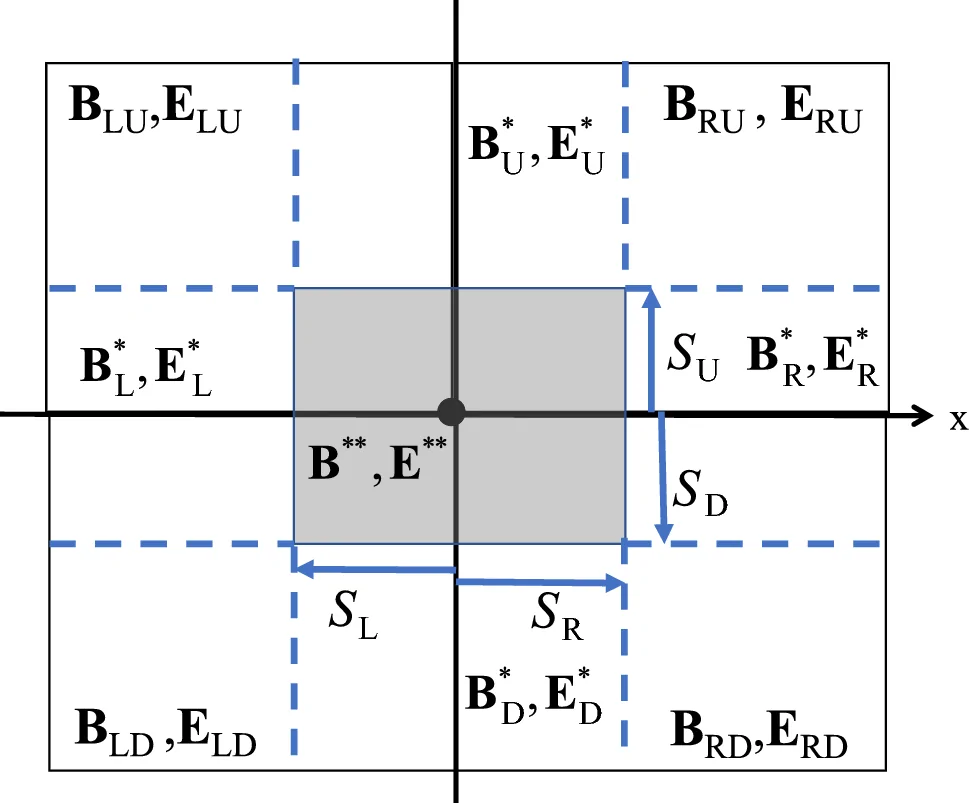
Same situation as Fig. 2. However, it shows the situation after the four incoming states start interacting with each other. Four 1D Riemann problems, shown by dashed lines, develop between the four pairs of incoming states. The resolved states from the 1D Riemann problems are shown by a superscript with a single star. The shaded region depicts the strongly interacting state that arises when the four 1D Riemann problems interact with one another. The strongly interacting state is shown by a superscript with a double star. We want to find the *z*-component of the electric field in the strongly interacting state. This gives us the *z*-component of the electric field at the *z*-edge, which is shown by the dot in this 2D projection

To keep the discussion general, we will assume that Eq. (1) is a sub-part of a larger PDE system which sets the wave speeds in all directions. In two dimensions, the input states are shown in Fig. 2 and the resulting wave structure of the 2D Riemann problem is shown in Fig. 3. Those figures also serve to explain the notation that is used here. The subscript “RU” stands for right-upper, “LU” stands for left-upper, “LD” stands for left-down, and “RD” stands for right-down. Figure 3 shows a wave model with speeds that span $\left[ {S_{\mathrm{L}} ,S_{\mathrm{R}} } \right] \times \left[ {S_{\mathrm{D}} ,S_{\mathrm{U}} } \right]$. While we use the HLL version of the Riemann solver, we will also be interested in the LLF variant of this Riemann solver given by setting $S_{\mathrm{R}} = - S_{\mathrm{L}} = S_{\mathrm{U}} = - S_{\mathrm{D}} = S$, where “*S*” is some estimate of the maximal wave speed for the waves around the edge of interest. We will also build in the continuity of the normal component of the magnetic field at the faces of the mesh from the very start, so that we have $B_{x\mathrm{RD}} = B_{x\mathrm{LD}} \equiv B_{x\mathrm{D}}$, $B_{x\mathrm{RU}} = B_{x\mathrm{LU}} \equiv B_{x\mathrm{U}}$, $B_{y\mathrm{RU}} = B_{y\mathrm{RD}} \equiv B_{y\mathrm{R}}$ and $B_{y\mathrm{LU}} = B_{y\mathrm{LD}} \equiv B_{y\mathrm{L}}$. This is also shown in Fig. 2. As a result, we realize that the only inputs to the 2D Riemann solver are the four facial magnetic fields $B_{x\mathrm{D}}$, $B_{x\mathrm{U}}$, $B_{y\mathrm{L}}$, and $B_{y\mathrm{R}}$ along with the four electric field components $E_{z\mathrm{RU}}$, $E_{z\mathrm{LU}}$, $E_{z\mathrm{LD}}$, and $E_{z\mathrm{RD}}$. This dramatically simplifies subsequent derivations. Consequently, the 1D HLL Riemann solver in the *x*-direction between the two upper states in Fig. 2 gives us the resolved state and flux, which we use to get

7a $$\begin{aligned} \left\{ \begin{aligned} & B_{x\mathrm{U}}^{*} = B_{x\mathrm{U}} { ;} \\& B_{y\mathrm{U}}^{*} = {{\left( {S_{\mathrm{R}} B_{y\mathrm{R}} - S_{\mathrm{L}} B_{y\mathrm{L}} } \right)} \mathord{\left/ {\vphantom {{\left( {S_{\mathrm{R}} B_{y\mathrm{R}} - S_{\mathrm{L}} B_{y\mathrm{L}} } \right)} {\left( {S_{\mathrm{R}} - S_{\mathrm{L}} } \right)}}} \right. \kern-0pt} {\left( {S_{\mathrm{R}} - S_{\mathrm{L}} } \right)}} + {{\left( {E_{z\mathrm{RU}} - E_{z\mathrm{LU}} } \right)} \mathord{\left/ {\vphantom {{\left( {E_{z\mathrm{RU}} - E_{zLU} } \right)} {\left( {S_{\mathrm{R}} - S_{\mathrm{L}} } \right)}}} \right. \kern-0pt} {\left( {S_{\mathrm{R}} - S_{\mathrm{L}} } \right)}}{ ;} \\& E_{z\mathrm{U}}^{*} = {{\left( {S_{\mathrm{R}} E_{z\mathrm{LU}} - S_{\mathrm{L}} E_{z\mathrm{RU}} } \right)} \mathord{\left/ {\vphantom {{\left( {S_{\mathrm{R}} E_{z\mathrm{LU}} - S_{\mathrm{L}} E_{z\mathrm{RU}} } \right)} {\left( {S_{\mathrm{R}} - S_{\mathrm{L}} } \right)}}} \right. \kern-0pt} {\left( {S_{\mathrm{R}} - S_{\mathrm{L}} } \right)}} - S_{\mathrm{R}} S_{\mathrm{L}} {{\left( {B_{y\mathrm{R}} - B_{y\mathrm{L}} } \right)} \mathord{\left/ {\vphantom {{\left( {B_{y\mathrm{R}} - B_{y\mathrm{L}} } \right)} {\left( {S_{\mathrm{R}} - S_{\mathrm{L}} } \right)}}} \right. \kern-0pt} {\left( {S_{\mathrm{R}} - S_{\mathrm{L}} } \right)}} \, {.} \end{aligned} \right. \end{aligned}$$

We can also use $S_{\mathrm{R}} = - S_{\mathrm{L}} = S$ to write the LLF variant of the above equation as

7b $$\begin{aligned} \left\{ \begin{aligned} & B_{x\mathrm{U}}^{*} = B_{x\mathrm{U}} { ;} \\& B_{y\mathrm{U}}^{*} = {{\left( {B_{y\mathrm{R}} + B_{y\mathrm{L}} } \right)} \mathord{\left/ {\vphantom {{\left( {B_{y\mathrm{R}} + B_{y\mathrm{L}} } \right)} 2}} \right. \kern-0pt} 2} + {{\left( {E_{z\mathrm{RU}} - E_{z\mathrm{LU}} } \right)} \mathord{\left/ {\vphantom {{\left( {E_{z\mathrm{RU}} - E_{z\mathrm{LU}} } \right)} {\left( {2S} \right)}}} \right. \kern-0pt} {\left( {2S} \right)}}{ ;} \\& E_{z\mathrm{U}}^{*} = {{\left( {E_{z\mathrm{LU}} + E_{z\mathrm{RU}} } \right)} \mathord{\left/ {\vphantom {{\left( {E_{z\mathrm{LU}} + E_{z\mathrm{RU}} } \right)} 2}} \right. \kern-0pt} 2} + S{{\left( {B_{y\mathrm{R}} - B_{y\mathrm{L}} } \right)} \mathord{\left/ {\vphantom {{\left( {B_{y\mathrm{R}} - B_{y\mathrm{L}} } \right)} 2}} \right. \kern-0pt} 2}{.} \end{aligned} \right. \end{aligned}$$

To get the analogous formulae for the *x*-directional Riemann solver between the two lower states in Fig. 2, we just set U→D in Eq. (7). When the 1D HLL Riemann solver in the *y*-direction is applied between the two right states in Fig. 2, we use the resolved state and flux to get

8a $$\begin{aligned} \left\{ \begin{aligned} & B_{x\mathrm{R}}^{*} = {{\left( {S_{\mathrm{U}} B_{x\mathrm{U}} - S_{\mathrm{D}} B_{x\mathrm{D}} } \right)} \mathord{\left/ {\vphantom {{\left( {S_{\mathrm{U}} B_{x\mathrm{U}} - S_{\mathrm{D}} B_{x\mathrm{D}} } \right)} {\left( {S_{\mathrm{U}} - S_{\mathrm{D}} } \right)}}} \right. \kern-0pt} {\left( {S_{\mathrm{U}} - S_{\mathrm{D}} } \right)}} - {{\left( {E_{z\mathrm{RU}} - E_{z\mathrm{RD}} } \right)} \mathord{\left/ {\vphantom {{\left( {E_{z\mathrm{RU}} - E_{z\mathrm{RD}} } \right)} {\left( {S_{\mathrm{U}} - S_{\mathrm{D}} } \right)}}} \right. \kern-0pt} {\left( {S_{\mathrm{U}} - S_{\mathrm{D}} } \right)}}{ ;} \\& B_{y\mathrm{R}}^{*} = B_{y\mathrm{R}} { ;} \\& E_{z\mathrm{R}}^{*} = {{\left( {S_{\mathrm{U}} E_{z\mathrm{RD}} - S_{\mathrm{D}} E_{z\mathrm{RU}} } \right)} \mathord{\left/ {\vphantom {{\left( {S_{\mathrm{U}} E_{z\mathrm{RD}} - S_{\mathrm{D}} E_{z\mathrm{RU}} } \right)} {\left( {S_{\mathrm{U}} - S_{\mathrm{D}} } \right)}}} \right. \kern-0pt} {\left( {S_{\mathrm{U}} - S_{\mathrm{D}} } \right)}} + S_{\mathrm{U}} S_{\mathrm{D}} {{\left( {B_{x\mathrm{U}} - B_{x\mathrm{D}} } \right)} \mathord{\left/ {\vphantom {{\left( {B_{x\mathrm{U}} - B_{x\mathrm{D}} } \right)} {\left( {S_{\mathrm{U}} - S_{\mathrm{D}} } \right)}}} \right. \kern-0pt} {\left( {S_{\mathrm{U}} - S_{\mathrm{D}} } \right)}} \, {.} \end{aligned} \right. \end{aligned}$$

We can also use $S_{\mathrm{U}} = - S_{\mathrm{D}} = S$ to write the LLF variant of the above equation as

8b $$\begin{aligned} \left\{ \begin{aligned} & B_{x\mathrm{R}}^{*} = {{\left( {B_{x\mathrm{U}} + B_{x\mathrm{D}} } \right)} \mathord{\left/ {\vphantom {{\left( {B_{x\mathrm{U}} + B_{x\mathrm{D}} } \right)} 2}} \right. \kern-0pt} 2} - {{\left( {E_{z\mathrm{RU}} - E_{z\mathrm{RD}} } \right)} \mathord{\left/ {\vphantom {{\left( {E_{z\mathrm{RU}} - E_{z\mathrm{RD}} } \right)} {\left( {2S} \right)}}} \right. \kern-0pt} {\left( {2S} \right)}}{ ;} \\& B_{y\mathrm{R}}^{*} = B_{y\mathrm{R}} { ;} \\& E_{z\mathrm{R}}^{*} = {{\left( {E_{z\mathrm{RD}} + E_{z\mathrm{RU}} } \right)} \mathord{\left/ {\vphantom {{\left( {E_{z\mathrm{RD}} + E_{z\mathrm{RU}} } \right)} 2}} \right. \kern-0pt} 2} - S{{\left( {B_{x\mathrm{U}} - B_{x\mathrm{D}} } \right)} \mathord{\left/ {\vphantom {{\left( {B_{x\mathrm{U}} - B_{x\mathrm{D}} } \right)} 2}} \right. \kern-0pt} 2}{.} \end{aligned} \right. \end{aligned}$$

To get the analogous formulae for the *y*-directional Riemann solver between the two left states in Fig. 2, we just set $\mathrm{R} \to \mathrm{L}$ in Eq. (8). Equations (7) and (8) give us the resolved states in Fig. 3. These are the states that circumscribe the strongly interacting state in Fig. 3.

The resolved state in Fig. 3 is most easily obtained by applying Eqs. (12), (13), and (14) from Balsara [15]. We synopsize the essential results in this paragraph. We consider a PDE of the form $$\partial_{t} {\mathbf{U}} + \partial_{x} {\mathbf{F}} + \partial_{y} {\mathbf{G}} = 0$$. When four states come together at the edge of the mesh, four 1D Riemann problems are formed. The 1D Riemann problems evolve self-similarly. However, those four 1D Riemann problems interact among themselves, resulting in a strongly interacting state that also evolves self-similarly. The strongly interacting state is shown by the variables with the double starred superscript in Fig. 3. Since the subsonic case is the most interesting case, and the one that occurs most frequently in the code, we are interested in that strongly interacting state. In the three ensuing equations, the strongly interacting state in Fig. 3 is thought to be covered by a coordinate system in the similarity variables $$\tilde{\xi } \equiv {x \mathord{\left/ {\vphantom {x t}} \right. \kern-0pt} t}$$ and $$\tilde{\psi } \equiv {y \mathord{\left/ {\vphantom {y t}} \right. \kern-0pt} t}$$. This coordinate system spans $$\left( {\tilde{\xi },\tilde{\psi }} \right) \in \left[ {S_{\mathrm{L}} ,S_{\mathrm{R}} } \right] \times \left[ {S_{\mathrm{D}} ,S_{\mathrm{U}} } \right]$$. The math becomes easier if we pick a coordinate system that is centered on the strongly interacting state. This is done with the help of linear transformations $$\xi \equiv {{\left( {\tilde{\xi } - \xi_{c} } \right)} \mathord{\left/ {\vphantom {{\left( {\tilde{\xi } - \xi_{c} } \right)} {\Delta \xi }}} \right. \kern-0pt} {\Delta \xi }}$$ and $$\psi \equiv {{\left( {\tilde{\psi } - \psi_{c} } \right)} \mathord{\left/ {\vphantom {{\left( {\tilde{\psi } - \psi_{c} } \right)} {\Delta \psi }}} \right. \kern-0pt} {\Delta \psi }}$$, with the definitions $$\Delta \xi \equiv S_{\mathrm{R}} - S_{\mathrm{L}}$$, $$\xi_{c} \equiv {{\left( {S_{\mathrm{R}} + S_{\mathrm{L}} } \right)} \mathord{\left/ {\vphantom {{\left( {S_{R} + S_{L} } \right)} 2}} \right. \kern-0pt} 2}$$, $$\Delta \psi \equiv S_{\mathrm{U}} - S_{\mathrm{D}}$$, and $$\psi_{c} \equiv {{\left( {S_{\mathrm{U}} + S_{\mathrm{D}} } \right)} \mathord{\left/ {\vphantom {{\left( {S_{\mathrm{U}} + S_{\mathrm{D}} } \right)} 2}} \right. \kern-0pt} 2}$$. This linear rescaling of the coordinates maps the wave speeds from $$\left[ {S_{\mathrm{L}} ,S_{\mathrm{R}} } \right] \times \left[ {S_{\mathrm{D}} ,S_{\mathrm{U}} } \right]$$ to $$\left[ { - 1/2,1/2} \right]^{2}$$. For ease of use, we specialize the equations for the strongly interacting state vector $${\mathbf{U}}^{**}$$ and its associated fluxes $${\mathbf{F}}^{**}$$ and $${\mathbf{G}}^{**}$$ for our current needs in this paper as

9 $${\mathbf{U}}^{**} = - \left[ \begin{gathered} \frac{1}{2\Delta \xi }\int\nolimits_{ - 1/2}^{1/2} {\left( {{\mathbf{F}}\left( {1/2,\psi } \right) - S_{\mathrm{R}} {\mathbf{U}}\left( {1/2,\psi } \right)} \right)\mathrm{d}\psi } - \frac{1}{2\Delta \xi }\int\nolimits_{ - 1/2}^{1/2} {\left( {{\mathbf{F}}\left( { - 1/2,\psi } \right) - S_{\mathrm{L}} {\mathbf{U}}\left( { - 1/2,\psi } \right)} \right)\mathrm{d}\psi } \hfill \\ + \frac{1}{2\Delta \psi }\int\nolimits_{ - 1/2}^{1/2} {\left( {{\mathbf{G}}\left( {\xi ,1/2} \right) - S_{\mathrm{U}} {\mathbf{U}}\left( {\xi ,1/2} \right)} \right)\mathrm{d}\xi } - \frac{1}{2\Delta \psi }\int\nolimits_{ - 1/2}^{1/2} {\left( {{\mathbf{G}}\left( {\xi , - 1/2} \right) - S_{\mathrm{D}} {\mathbf{U}}\left( {\xi , - 1/2} \right)} \right)\mathrm{d}\xi } \hfill \\ \end{gathered} \right],$$

10 $${\mathbf{F}}^{**} = \xi_{c} {\mathbf{U}}^{**} + \left[ \begin{gathered} \frac{1}{2}\int\nolimits_{ - 1/2}^{1/2} {\left( {{\mathbf{F}}\left( {1/2,\psi } \right) - S_{\mathrm{R}} {\mathbf{U}}\left( {1/2,\psi } \right)} \right)\mathrm{d}\psi } + \frac{1}{2}\int\nolimits_{ - 1/2}^{1/2} {\left( {{\mathbf{F}}\left( { - 1/2,\psi } \right) - S_{\mathrm{L}} {\mathbf{U}}\left( { - 1/2,\psi } \right)} \right)\mathrm{d}\psi } \hfill \\ + \frac{\Delta \xi }{{\Delta \psi }}\int\nolimits_{ - 1/2}^{1/2} {\xi \left( {{\mathbf{G}}\left( {\xi ,1/2} \right) - S_{\mathrm{U}} {\mathbf{U}}\left( {\xi ,1/2} \right)} \right)\mathrm{d}\xi } - \frac{\Delta \xi }{{\Delta \psi }}\int\nolimits_{ - 1/2}^{1/2} {\xi \left( {{\mathbf{G}}\left( {\xi , - 1/2} \right) - S_{\mathrm{D}} {\mathbf{U}}\left( {\xi , - 1/2} \right)} \right)\mathrm{d}\xi } \hfill \\ \end{gathered} \right],$$

11 $${\mathbf{G}}^{**} = \psi_{c} {\mathbf{U}}^{**} + \left[ \begin{gathered} \frac{\Delta \psi }{{\Delta \xi }}\int\nolimits_{ - 1/2}^{1/2} {\psi \left( {{\mathbf{F}}\left( {1/2,\psi } \right) - S_{\mathrm{R}} {\mathbf{U}}\left( {1/2,\psi } \right)} \right)\mathrm{d}\psi } - \frac{\Delta \psi }{{\Delta \xi }}\int\nolimits_{ - 1/2}^{1/2} {\psi \left( {{\mathbf{F}}\left( { - 1/2,\psi } \right) - S_{\mathrm{L}} {\mathbf{U}}\left( { - 1/2,\psi } \right)} \right)\mathrm{d}\psi } \hfill \\ + \frac{1}{2}\int\nolimits_{ - 1/2}^{1/2} {\left( {{\mathbf{G}}\left( {\xi ,1/2} \right) - S_{\mathrm{U}} {\mathbf{U}}\left( {\xi ,1/2} \right)} \right)\mathrm{d}\xi } + \frac{1}{2}\int\nolimits_{ - 1/2}^{1/2} {\left( {{\mathbf{G}}\left( {\xi , - 1/2} \right) - S_{\mathrm{D}} {\mathbf{U}}\left( {\xi , - 1/2} \right)} \right)\mathrm{d}\xi } \hfill \\ \end{gathered} \right].$$

$$E_{z}^{**}$$ can now be obtained from taking the negative of the second component of $${\mathbf{F}}^{**}$$ or by taking the first component of $${\mathbf{G}}^{**}$$. Here, we have simply stated the above three equations with the minimal requisite explanation. In Balsara [15], detailed explanations for the derivations are given.

### Edge-Aligned Electric Field for the Multidimensional LLF Riemann Solver

We are first interested in the LLF limit. Notice that $$E_{z}^{**}$$ can be obtained as the negative of the second component of the resolved *x*-flux. This would require the use of Eq. (10). Alternatively, $$E_{z}^{**}$$ can be obtained as the first component of the resolved *y*-flux. This would require the use of Eq. (11). Regardless of the two alternative ways for obtaining $$E_{z}^{**}$$, the mathematics gives us the same result. We get

12 $$E_{z}^{**} = {{\left( {E_{z\mathrm{RU}} + E_{z\mathrm{LU}} + E_{z\mathrm{LD}} + E_{z\mathrm{RD}} } \right)} \mathord{\left/ {\vphantom {{\left( {E_{z\mathrm{RU}} + E_{z\mathrm{LU}} + E_{z\mathrm{LD}} + E_{z\mathrm{RD}} } \right)} 4}} \right. \kern-0pt} 4}{ + }S{{\left[ {B_{x\mathrm{D}} - B_{x\mathrm{U}} + B_{y\mathrm{R}} - B_{y\mathrm{L}} } \right]} \mathord{\left/ {\vphantom {{\left[ {B_{x\mathrm{D}} - B_{x\mathrm{U}} + B_{y\mathrm{R}} - B_{y\mathrm{L}} } \right]} 2}} \right. \kern-0pt} 2}.$$

For a 2D LLF Riemann solver, $$E_{z}^{**}$$ is also the same as he numerically stabilized *z*-component of the electric field given by $$E_{z}^{\mathrm{num}}$$. We begin by pointing out that when the variation in the solution is restricted entirely to the *x*-direction or *y*-direction, Eq. (12) reduces correctly to the 1D limit. It is, therefore, consistent. Equation (12) then shows us that the arithmetically averaged round bracket, i.e., $${{\left( {E_{z\mathrm{RU}} + E_{z\mathrm{LU}} + E_{z\mathrm{LD}} + E_{z\mathrm{RD}} } \right)} \mathord{\left/ {\vphantom {{\left( {E_{z\mathrm{RU}} + E_{z\mathrm{LU}} + E_{z\mathrm{LD}} + E_{z\mathrm{RD}} } \right)} 4}} \right. \kern-0pt} 4}$$, is the central term. It is free of dissipation and carries the *z*-component of the electric field that we need at the *z*-edges of the mesh. This is the electric field that can be evaluated at the zone centers and interpolated in a 2D WENO fashion to the centers of the *z*-edges, as documented in the figure caption of Fig. 2. WENO is useful for doing this, because it ensures that we pick up the largest 2D stencil that is smooth while avoiding possible stencils that might be non-smooth. To do this, we will have to design a pointwise 2D interpolation strategy for WENO; an analogous 2D reconstruction strategy for WENO has been presented in Balsara et al. [33]. The round bracket in Eq. (12) restores consistency to the *z*-component of the electric field; however, to stabilize it, we need a contribution from the dissipation. The dissipation in Eq. (12) is entirely carried by the square bracket, i.e., $${{\left[ {B_{x\mathrm{D}} - B_{x\mathrm{U}} + B_{y\mathrm{R}} - B_{y\mathrm{L}} } \right]} \mathord{\left/ {\vphantom {{\left[ {B_{x\mathrm{D}} - B_{x\mathrm{U}} + B_{y\mathrm{R}} - B_{y\mathrm{L}} } \right]} 2}} \right. \kern-0pt} 2}$$. If simple face-centered values are used, we will get the first order dissipation. This dissipation can be very high. However, we can do much better if the facially reconstructed magnetic field components are evaluated at the zone edges. This can be obtained from the 2D WENO reconstruction of the normal components of the magnetic fields within the 2D faces. We, therefore, see that the additional WENO interpolation and reconstruction strategies that need to be invented are very few or already in hand. They are also very light-weight, because the interpolation does not need to be projected into characteristic space; rather, it is only applied to a single component of the solution vector or to a single component of the flux vector.

We can additionally write out the *x*-, *y*-, and *z*-components of the resolved magnetic field at the *z*-edge using Eq. (9). For $$B_{x}^{**}$$, we get

13 $$B_{x}^{**} = {{\left( {B_{x\mathrm{U}} + B_{x\mathrm{D}} } \right)} \mathord{\left/ {\vphantom {{\left( {B_{x\mathrm{U}} + B_{x\mathrm{D}} } \right)} 2}} \right. \kern-0pt} 2} + {{\left( {E_{z\mathrm{LD}} - E_{z\mathrm{LU}} + E_{z\mathrm{RD}} - E_{z\mathrm{RU}} } \right)} \mathord{\left/ {\vphantom {{\left( {E_{z\mathrm{LD}} - E_{z\mathrm{LU}} + E_{z\mathrm{RD}} - E_{z\mathrm{RU}} } \right)} {\left( {4S} \right)}}} \right. \kern-0pt} {\left( {4S} \right)}}.$$

We can see the familiar structure that emerges in a Riemann solver where the flux terms also influence the resolved states in the Riemann problem. For $$B_{y}^{**}$$, we get

14 $$B_{y}^{**} = {{\left( {B_{y\mathrm{R}} + B_{y\mathrm{L}} } \right)} \mathord{\left/ {\vphantom {{\left( {B_{y\mathrm{R}} + B_{y\mathrm{L}} } \right)} 2}} \right. \kern-0pt} 2}{ + }{{\left( {E_{z\mathrm{RD}} - E_{z\mathrm{LD}} + E_{z\mathrm{RU}} - E_{z\mathrm{LU}} } \right)} \mathord{\left/ {\vphantom {{\left( {E_{z\mathrm{RD}} - E_{z\mathrm{LD}} + E_{z\mathrm{RU}} - E_{z\mathrm{LU}} } \right)} {\left( {4S} \right)}}} \right. \kern-0pt} {\left( {4S} \right)}}.$$

For $$B_{z}^{**}$$, we get

15 $$\begin{gathered} B_{z}^{**} = {{\left( {B_{z\mathrm{RU}} + B_{z\mathrm{LU}} + B_{z\mathrm{LD}} + B_{z\mathrm{RD}} } \right)} \mathord{\left/ {\vphantom {{\left( {B_{z\mathrm{RU}} + B_{z\mathrm{LU}} + B_{z\mathrm{LD}} + B_{z\mathrm{RD}} } \right)} 4}} \right. \kern-0pt} 4} + {{\left( { - E_{x\mathrm{D}}^{*} + E_{x\mathrm{U}}^{*} + E_{y\mathrm{L}}^{*} - E_{y\mathrm{R}}^{*} } \right)} \mathord{\left/ {\vphantom {{\left( { - E_{x\mathrm{D}}^{*} + E_{x\mathrm{U}}^{*} + E_{y\mathrm{L}}^{*} - E_{y\mathrm{R}}^{*} } \right)} {\left( {4S} \right)}}} \right. \kern-0pt} {\left( {4S} \right)}} \hfill \\ \qquad \quad { + }{{\left[ {\left( {E_{x\mathrm{RU}} + E_{x\mathrm{LU}} } \right) - \left( {E_{x\mathrm{RD}} + E_{x\mathrm{LD}} } \right) - \left( {E_{y\mathrm{RU}} + E_{y\mathrm{RD}} } \right) + \left( {E_{y\mathrm{LU}} + E_{y\mathrm{LD}} } \right)} \right]} \mathord{\left/ {\vphantom {{\left[ {\left( {E_{x\mathrm{RU}} + E_{x\mathrm{LU}} } \right) - \left( {E_{x\mathrm{RD}} + E_{x\mathrm{LD}} } \right) - \left( {E_{y\mathrm{RU}} + E_{y\mathrm{RD}} } \right) + \left( {E_{y\mathrm{LU}} + E_{y\mathrm{LD}} } \right)} \right]} {\left( {8S} \right)}}} \right. \kern-0pt} {\left( {8S} \right)}}. \hfill \\ \end{gathered}$$

Equation (15) shows that for an involution-constrained system, $$B_{z}^{**}$$ cannot be obtained from the Riemann solver per se, unless more components of the parent PDE are used. However, Eqs. (12), (13), and (14) show that $$E_{z}^{**}$$, $$B_{x}^{**}$$, and $$B_{y}^{**}$$ can indeed be obtained from the multidimensional Riemann solver and that is all that we actually need to make progress with the scheme design. In fairness, even $$B_{x}^{**}$$ and $$B_{y}^{**}$$ are not useful for the scheme that we design in this paper. However, we provide them here because they could become very useful when the inclusion of parabolic terms is considered.

### Edge-Aligned Electric Field for the Multidimensional HLL Riemann Solver

For some PDEs, such as Maxwell's equations, where the maximal signal speed is always the speed of light, we will always have a subsonic Riemann solver. For other PDEs, such as the MHD equations, the CGL equations, and the relativistic MHD equations, the flow speed can sometimes be strongly supersonic. The LLF Riemann solver can become problematic in such situations, because the associated wave model opens up the Riemann fan much more than is warranted. The HLL Riemann solver is very useful in such situations, because it has a well-defined supersonic limit. The same is true for the multidimensional HLL Riemann solver. For this reason, we describe the 2D HLL Riemann solver as it applies to Eq. (6).

Using Eqs. (7HLL) and (8HLL) and their analogs in Eq. (9), we get the resolved *x*-component of the magnetic field in the strongly interacting state as

16 $$B_{x}^{**} = {{\left( {S_{\mathrm{U}} B_{x\mathrm{U}} - S_{\mathrm{D}} B_{x\mathrm{D}} } \right)} \mathord{\left/ {\vphantom {{\left( {S_{\mathrm{U}} B_{x\mathrm{U}} - S_{\mathrm{D}} B_{x\mathrm{D}} } \right)} {\left( {S_{\mathrm{U}} - S_{\mathrm{D}} } \right)}}} \right. \kern-0pt} {\left( {S_{\mathrm{U}} - S_{\mathrm{D}} } \right)}} + {{\left( {E_{z\mathrm{LD}} - E_{z\mathrm{LU}} + E_{z\mathrm{RD}} - E_{z\mathrm{RU}} } \right)} \mathord{\left/ {\vphantom {{\left( {E_{z\mathrm{LD}} - E_{z\mathrm{LU}} + E_{z\mathrm{RD}} - E_{z\mathrm{RU}} } \right)} {\left( {2\left( {S_{\mathrm{U}} - S_{\mathrm{D}} } \right)} \right)}}} \right. \kern-0pt} {\left( {2\left( {S_{\mathrm{U}} - S_{\mathrm{D}} } \right)} \right)}}.$$

The first term in the above equation has a nice geometrical interpretation. It represents the area-weighted average of the *x*-component of the magnetic field over the wave model shown in Fig. 3. The second term in Eq. (16) can be interpreted as a contribution from the dissipation and is quite analogous to the second term in Eq. (13). We can also obtain the resolved *y*-component of the magnetic field in the strongly interacting state as

17 $$B_{y}^{**} = {{\left( {S_{\mathrm{R}} B_{y\mathrm{R}} - S_{\mathrm{L}} B_{y\mathrm{L}} } \right)} \mathord{\left/ {\vphantom {{\left( {S_{\mathrm{R}} B_{y\mathrm{R}} - S_{\mathrm{L}} B_{y\mathrm{L}} } \right)} {\left( {S_{\mathrm{R}} - S_{\mathrm{L}} } \right)}}} \right. \kern-0pt} {\left( {S_{\mathrm{R}} - S_{\mathrm{L}} } \right)}} + {{\left( { - E_{z\mathrm{LD}} - E_{z\mathrm{LU}} + E_{z\mathrm{RD}} + E_{z\mathrm{RU}} } \right)} \mathord{\left/ {\vphantom {{\left( { - E_{z\mathrm{LD}} - E_{z\mathrm{LU}} + E_{z\mathrm{RD}} + E_{z\mathrm{RU}} } \right)} {\left( {2\left( {S_{\mathrm{R}} - S_{\mathrm{L}} } \right)} \right)}}} \right. \kern-0pt} {\left( {2\left( {S_{\mathrm{R}} - S_{\mathrm{L}} } \right)} \right)}}.$$

The first and second terms of Eq. (17) can be similarly interpreted. As seen from Eq. (15), it is not valuable to try to obtain $$B_{z}^{**}$$, nor is it valuable for the developments that are to follow. The resolved *z*-component of the electric field in the strongly interacting state can be obtained from the negative of the second component of the *x*-flux as obtained from Eq. (10). We then get our first approximation for the resolved *z*-component of the electric field

18 $$\begin{gathered} E_{z;1}^{**} = - {{\left( {S_{\mathrm{R}} + S_{\mathrm{L}} } \right)B_{{y}}^{**} } \mathord{\left/ {\vphantom {{\left( {S_{\mathrm{R}} + S_{\mathrm{L}} } \right)B_{y}^{**} } 2}} \right. \kern-0pt} 2} + {{\left( {S_{\mathrm{U}} \left( {E_{z\mathrm{LD}} + E_{z\mathrm{RD}} } \right) - S_{\mathrm{D}} \left( {E_{z\mathrm{LU}} + E_{z\mathrm{RU}} } \right)} \right)} \mathord{\left/ {\vphantom {{\left( {S_{\mathrm{U}} \left( {E_{z\mathrm{LD}} + E_{z\mathrm{RD}} } \right) - S_{\mathrm{D}} \left( {E_{z\mathrm{LU}} + E_{z\mathrm{RU}} } \right)} \right)} {\left( {2\left( {S_{\mathrm{U}} - S_{\mathrm{D}} } \right)} \right)}}} \right. \kern-0pt} {\left( {2\left( {S_{\mathrm{U}} - S_{\mathrm{D}} } \right)} \right)}} \hfill \\ \qquad \quad \, - S_{\mathrm{U}} S_{\mathrm{D}} {{\left( {B_{x\mathrm{D}} - B_{x\mathrm{U}} } \right)} \mathord{\left/ {\vphantom {{\left( {B_{x\mathrm{D}} - B_{x\mathrm{U}} } \right)} {\left( {S_{\mathrm{U}} - S_{\mathrm{D}} } \right)}}} \right. \kern-0pt} {\left( {S_{\mathrm{U}} - S_{\mathrm{D}} } \right)}} + {{\left( {S_{\mathrm{R}} B_{y\mathrm{R}} + S_{\mathrm{L}} B_{y\mathrm{L}} } \right)} \mathord{\left/ {\vphantom {{\left( {S_{\mathrm{R}} B_{y\mathrm{R}} + S_{\mathrm{L}} B_{y\mathrm{L}} } \right)} 2}} \right. \kern-0pt} 2}. \hfill \\ \end{gathered}$$

The resolved *z*-component of the electric field in the strongly interacting state can also be obtained from the first component of the *y*-flux as obtained from Eq. (11). We then get our second approximation for the resolved *z*-component of the electric field

19 $$\begin{gathered} E_{z;2}^{**} = {{\left( {S_{\mathrm{U}} + S_{\mathrm{D}} } \right)B_{x}^{**} } \mathord{\left/ {\vphantom {{\left( {S_{\mathrm{U}} + S_{\mathrm{D}} } \right)B_{x}^{**} } 2}} \right. \kern-0pt} 2} + {{\left( {S_{\mathrm{R}} \left( {E_{z\mathrm{LD}} + E_{z\mathrm{LU}} } \right) - S_{\mathrm{L}} \left( {E_{z\mathrm{RD}} + E_{z\mathrm{RU}} } \right)} \right)} \mathord{\left/ {\vphantom {{\left( {S_{\mathrm{R}} \left( {E_{z\mathrm{LD}} + E_{z\mathrm{LU}} } \right) - S_{\mathrm{L}} \left( {E_{z\mathrm{RD}} + E_{z\mathrm{RU}} } \right)} \right)} {\left( {2\left( {S_{\mathrm{R}} - S_{\mathrm{L}} } \right)} \right)}}} \right. \kern-0pt} {\left( {2\left( {S_{\mathrm{R}} - S_{\mathrm{L}} } \right)} \right)}} \hfill \\ \qquad \quad - {{\left( {S_{\mathrm{U}} B_{x\mathrm{U}} + S_{\mathrm{D}} B_{x\mathrm{D}} } \right)} \mathord{\left/ {\vphantom {{\left( {S_{\mathrm{U}} B_{x\mathrm{U}} + S_{\mathrm{D}} B_{x\mathrm{D}} } \right)} 2}} \right. \kern-0pt} 2} - S_{\mathrm{R}} S_{\mathrm{L}} {{\left( {B_{y\mathrm{R}} - B_{y\mathrm{L}} } \right)} \mathord{\left/ {\vphantom {{\left( {B_{y\mathrm{R}} - B_{y\mathrm{L}} } \right)} {\left( {S_{\mathrm{R}} - S_{\mathrm{L}} } \right)}}} \right. \kern-0pt} {\left( {S_{\mathrm{R}} - S_{\mathrm{L}} } \right)}}. \hfill \\ \end{gathered}$$

Notice that when the wave speeds revert to their LLF limits, $S_{\mathrm{R}} = - S_{\mathrm{L}} = S_{\mathrm{U}} = - S_{\mathrm{D}} = S$, Eqs. (18) and (19) revert to the LLF formula in Eq. (12). For the HLL case, the expressions in Eqs. (18) and (19) are not identical and it is best to take the arithmetic average of the two expressions as our resolved *z*-component of the electric field in the strongly interacting state.

**Fig. 4:**
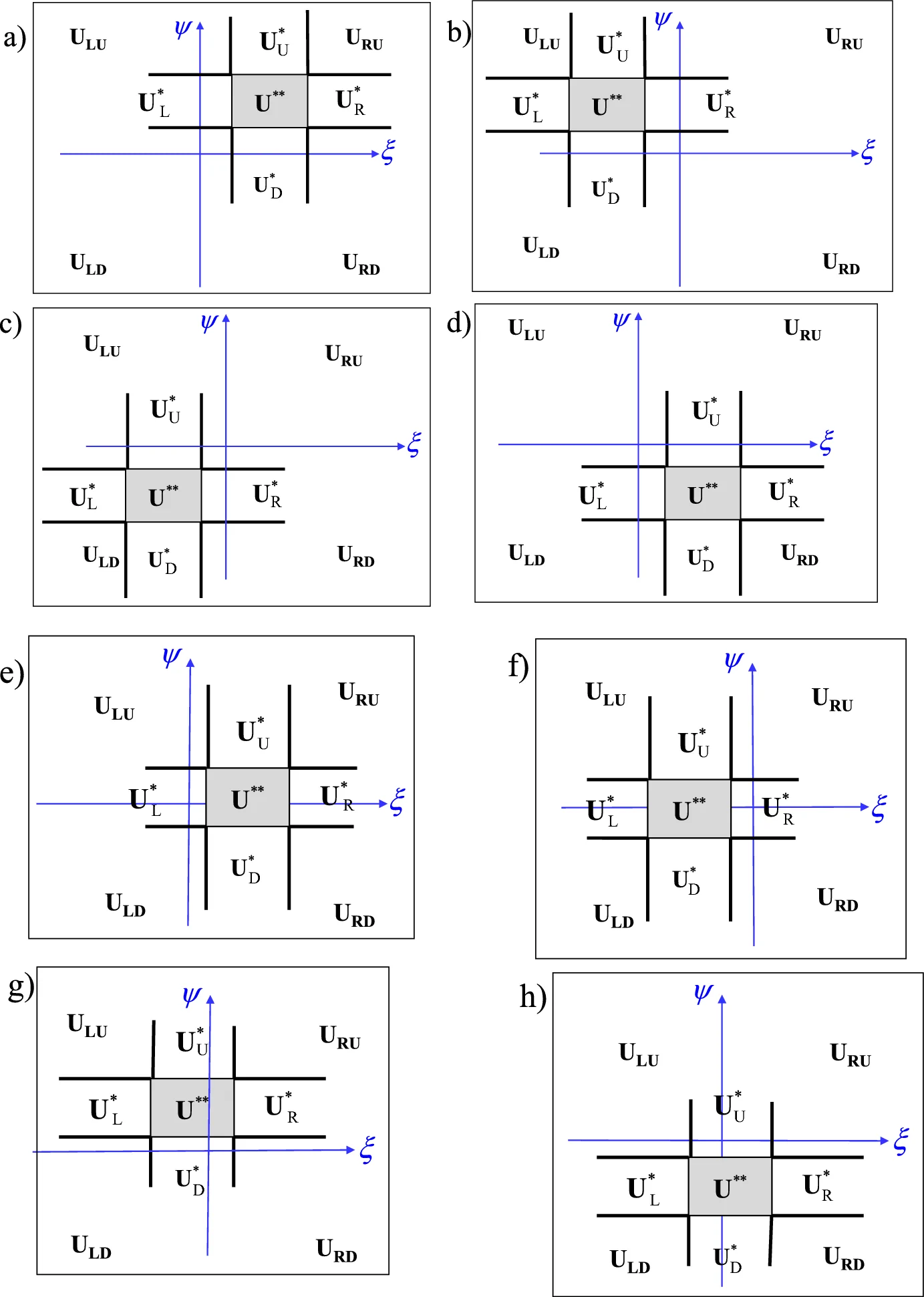
**a–d** Four cases that are fully supersonic in both directions. The axes, in similarity variables, are shown in blue. The wave model is shown in black. **e–h** Four cases that are fully supersonic in only one of the two directions. The axes, in similarity variables, are shown in blue. The wave model is shown in black

The LLF Riemann solver in one dimension or two dimensions is sometimes a slightly weaker option, because it lacks a supersonic limit. The above expressions show us how to obtain the edge-aligned *z*-component of the electric field from the 2D HLL Riemann solver in the fully subsonic case where $S_{\mathrm{L}} \leqslant 0 \leqslant S_{\mathrm{R}}$ and $S_{\mathrm{D}} \leqslant 0 \leqslant S_{\mathrm{U}}$, as shown in Fig. 3. However, there are also eight possible supersonic cases. They are shown in Fig. 4. The resolved *z*-component of the electric field that is to be used for numerical work, $E_{z}^{{{\mathrm{num}}}}$, can then be obtained by the following ninefold stack of conditionals that can be evaluated as:

20 $$\begin{gathered} {\mathrm{if}}\;\left( {S_{\mathrm{L}} \geqslant 0{\text{ and }}S_{\mathrm{D}} \geqslant 0} \right)\;{\mathrm{then}} \hfill \\ \, E_{z}^{{{\mathrm{num}}}} = E_{z\mathrm{LD}} {\text{; return;}} \hfill \\ {\mathrm{else}}\;{\mathrm{if}}\;\left( {S_{\mathrm{R}} \leqslant 0{\text{ and }}S_{\mathrm{D}} \geqslant 0} \right)\;{\mathrm{then}} \hfill \\ \, E_{z}^{{{\mathrm{num}}}} = E_{z\mathrm{RD}} {\text{; return;}} \hfill \\ {\mathrm{else}}\;{\mathrm{if}}\;\left( {S_{\mathrm{R}} \leqslant 0{\text{ and }}S_{\mathrm{U}} \leqslant 0} \right)\;{\mathrm{then}} \hfill \\ \, E_{{\mathrm{z}}}^{{{\mathrm{num}}}} = E_{z\mathrm{RU}} {\text{; return;}} \hfill \\ {\mathrm{else}}\;{\mathrm{if}}\;\left( {S_{\mathrm{L}} \geqslant 0{\text{ and }}S_{\mathrm{U}} \leqslant 0} \right)\;{\mathrm{then}} \hfill \\ \, E_{z}^{{{\mathrm{num}}}} = E_{z\mathrm{LU}} {\text{; return;}} \hfill \\ {\mathrm{else}}\;{\mathrm{if}}\;\left( {S_{\mathrm{L}} \geqslant 0} \right)\;{\mathrm{then}} \hfill \\ \, E_{z}^{{{\mathrm{num}}}} = E_{z\mathrm{L}}^{*} {\text{; return;}} \hfill \\ {\mathrm{else}}\;{\mathrm{if}}\;\left( {S_{\mathrm{R}} \leqslant 0} \right)\;{\mathrm{then}} \hfill \\ \, E_{z}^{{{\mathrm{num}}}} = E_{z\mathrm{R}}^{*} {\text{; return;}} \hfill \\ {\mathrm{else}}\;{\mathrm{if}}\;\left( {S_{\mathrm{D}} \geqslant 0} \right)\;{\mathrm{then}} \hfill \\ \, E_{z}^{{{\mathrm{num}}}} = E_{z\mathrm{D}}^{*} {\text{; return;}} \hfill \\ {\mathrm{else}}\;{\mathrm{if}}\;\left( {S_{\mathrm{U}} \leqslant 0} \right)\;{\mathrm{then}} \hfill \\ \, E_{z}^{{{\mathrm{num}}}} = E_{z\mathrm{U}}^{*} {\text{; return;}} \hfill \\ {\mathrm{else}} \hfill \\ \, E_{z}^{{{\mathrm{num}}}} = {{\left( {E_{z;1}^{**} + E_{z;2}^{**} } \right)} \mathord{\left/ {\vphantom {{\left( {E_{z;1}^{**} + E_{z;2}^{**} } \right)} 2}} \right. \kern-0pt} 2}{\text{; return;}} \hfill \\ {\mathrm{end}}\;{\mathrm{if}} \hfill \\ \end{gathered}$$

The subsonic limit, provided by the strongly interacting state in the last conditional above, is the most important case in the design of a multidimensional Riemann solver, since most problems operate in that limit most of the time. The stack of conditionals in the above equation is designed to provide optimal efficiency in computer code. The conditionals in the above stack are designed to be mutually exclusive. As soon as one of the conditionals checks out as positive, the subroutine should evaluate that numerical $E_{z}^{{{\mathrm{num}}}}$ and return without considering the subsequent conditionals. Therefore, only the variable that occurs within one of the conditional statements needs to be evaluated when that particular conditional is invoked.

## Step-by-Step Implementation of the Scheme

All the requisite pieces that go into the algorithm are now in hand. Here, we describe an AFD-WENO scheme that uses the pointwise collocated zone-centered variables and facially averaged normal components of vector fields that are divergence-constraint preserving. (If the PDE system does not have any non-conservative products, the AFD-WENO formulation may even be replaced with an FD-WENO formulation. This is accomplished by replacing Step 3 below with a standard flux-reconstruction-based FD-WENO algorithm to obtain fluxes.) In general, the conserved fluid variables are zone-centered point values, while the divergence-preserving normal components of the vector fields are facially averaged. These are the primal variables of the scheme. This is the most economical choice and it allows us to design a scheme where the zone-centered variables follow the AFD-WENO update, while the facially collocated variables will be updated in an FV sense. While this split approach may seem odd, it has two major advantages. First, it retains most of the efficiency advantage of the AFD-WENO scheme; since this is a costly step, it yields substantial savings. Second, since high-order divergence-preserving AMR of vector fields is a solved problem (see Balsara et al. [33], Balsara and Sarris [34]), all those advantages are retained. We assume a 3D Cartesian mesh with $N_{x} \times N_{y} \times N_{z}$ zones with uniform zone sizes Δx, Δy, and Δz in the *x*-, *y*-, and *z*-directions, respectively. To keep the notation tractable, we will establish a convention that applies to this section. We will denote averaged quantities with capital letters with an overbar, while we denote point values with corresponding small letters. Thus, we start with zone-centered, pointwise, conserved variables ${\mathbf{u}}_{i,j,k}$ for $\left( {i,j,k} \right) \in \left[ {1,N_{x} } \right] \times \left[ {1,N_{y} } \right] \times \left[ {1,N_{z} } \right]$ and facially averaged variables for the magnetic field denoted by ${\overline{\mathrm{B}}}_{x;i + 1/2,j,k}$ for $\left( {i,j,k} \right) \in \left[ {0,N_{x} } \right] \times \left[ {1,N_{y} } \right] \times \left[ {1,N_{z} } \right]$, ${\overline{\mathrm{B}}}_{y;i,j + 1/2,k}$ for $\left( {i,j,k} \right) \in \left[ {1,N_{x} } \right] \times \left[ {0,N_{y} } \right] \times \left[ {1,N_{z} } \right]$ and ${\overline{\mathrm{B}}}_{z;i,j,k + 1/2}$ for $\left( {i,j,k} \right) \in \left[ {1,N_{x} } \right] \times \left[ {1,N_{y} } \right] \times \left[ {0,N_{z} } \right]$. Notice the difference between the zone-centered pointwise small letters and the facially averaged capital letters with overbars. Here, we describe the algorithm within the context of the ultra-simple 2D LLF Riemann solver, because that is what most people will implement first. (Since this is an AFD-WENO scheme, it can accommodate any type of pointwise Riemann solver. Therefore, one can initially implement a 2D LLF Riemann solver and subsequently replace it with a 2D HLL Riemann solver.)

We illustrate the algorithm at the fifth order, but all the steps are fully generalizable to all orders. The algorithm proceeds with the following steps.

**Step 1) 2D Reconstruction of Facial B Field Components to Obtain Face-Centered Point Values** At each face, we can make the area-averaged, 2D reconstruction for the facial component of the magnetic field. The 2D high-order WENO-based reconstruction strategy that is needed for this step has already been described in the supplement to Balsara et al. [33]. For example, at the *x*-face, and up to the 5th order, we can make a 2D FV-WENO reconstruction of all the facial modes as

21 $$\begin{aligned} B_{x;i + 1/2,j,k} \left( {y,z} \right) = \; & {\overline{\mathrm{B}}}_{x;i + 1/2,j,k} + b_{y} y + b_{z} z + b_{yy} \left( {y^{2} - 1/12} \right) + b_{zz} \left( {z^{2} - 1/12} \right) + b_{yz} yz \\& { + }b_{yyy} \left( {y^{3} - 3y/20} \right) + b_{zzz} \left( {z^{3} - 3z/20} \right) + b_{yyz} \left( {y^{2} - 1/12} \right)z + b_{yzz} y\left( {z^{2} - 1/12} \right) \\& { + }b_{yyyy} \left( {y^{4} - 3y^{2} /14 + 3/560} \right) + b_{zzzz} \left( {z^{4} - 3z^{2} /14 + 3/560} \right) + b_{yyyz} \left( {y^{3} - 3y/20} \right)z \\& { + }b_{yzzz} y\left( {z^{3} - 3z/20} \right) + b_{yyzz} \left( {y^{2} - 1/12} \right)\left( {z^{2} - 1/12} \right). \end{aligned}$$

Note that because this is a 2D area-averaged WENO reconstruction, the area-averaged quantity, ${\overline{\mathrm{B}}}_{x;i + 1/2,j,k}$, is set. The WENO reconstruction is only tasked with finding the best approximations for the other coefficients on the right-hand side of Eq. (21). From Eq. (21), we can evaluate and store the point value of the *x*-component of the magnetic field at the center of the *x*-face as

22 $$\begin{aligned}b_{x;i + 1/2,j,k} &= B_{x;i + 1/2,j,k} \left( {y = 0,z = 0} \right) \\& = {\overline{\mathrm{B}}}_{x;i + 1/2,j,k} - b_{yy} /12 - b_{zz} /12 + 3 \, b_{yyyy} /560 + 3 \, b_{zzzz} /560 + b_{yyzz} /144.\end{aligned}$$

Analogously, we can obtain $b_{y;i,j + 1/2,k}$ at the center of the *y*-face and $b_{z;i,j,k + 1/2}$ at the center of the *z*-face.

Equation (21) can also be used to evaluate the facial magnetic field component anywhere within a face. Specifically, we also use Eq. (21) to evaluate $B_{x;i + 1/2,j,k} \; \left( {y = 0,z = 1/2} \right)$, $B_{x;i + 1/2,j,k}\; \left( {y = 0,z = - 1/2} \right)$, $B_{x;i + 1/2,j,k}\; \left( {y = 1/2,z = 0} \right)$, and $B_{x;i + 1/2,j,k}\; \left( {y = - 1/2,z = 0} \right)$ at the edge centers of the four edges that surround the *x*-face in question. These four values should be stored for each *x*-face. Similar evaluations can be made and stored at the *y*- and *z*-faces. These edge-centered evaluations of the magnetic fields will help us with the evaluation of the dissipation terms for the numerical electric field, as shown in Eq. (23) of Step 6.

**Step 2) 1D Interpolation from Facial Point Values to Zone-Centered Point Values** With these facial point values in hand from Eq. (22), we can use the special type of 1D WENO interpolation that was developed in Sect. 4 of Balsara et al. [19] to obtain point values of $b_{x;i,j,k}$, $b_{y;i,j,k}$, and $b_{z;i,j,k}$ at the zone centers. (To take MHD as an example, ${\mathbf{u}}_{i,j,k}$ can be used to obtain the pointwise fluid density, velocity, and pressure at each zone center. The interpolated $b_{x;i,j,k}$, $b_{y;i,j,k}$, and $b_{z;i,j,k}$ are also available through this step, so we have all the MHD variables that are needed at the zone centers.) This allows us to evaluate pointwise values for the fluxes, electric fields, and any other variables that we might need at the zone centers.

**Fig. 5:**
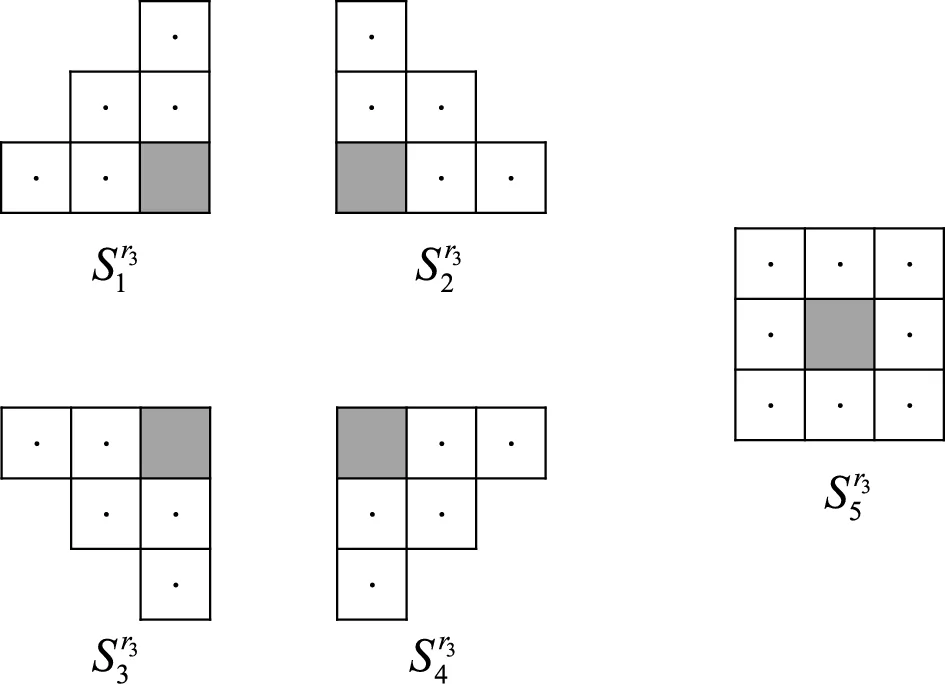
Four one-sided third-order and one central third-order stencil for the 2D-WENO interpolation. The (*i*,*j*)^th^ computational cell is denoted by the shaded region. The zone-centered point values of the neighboring zones are denoted by “centered-dot”

**Fig. 6:**
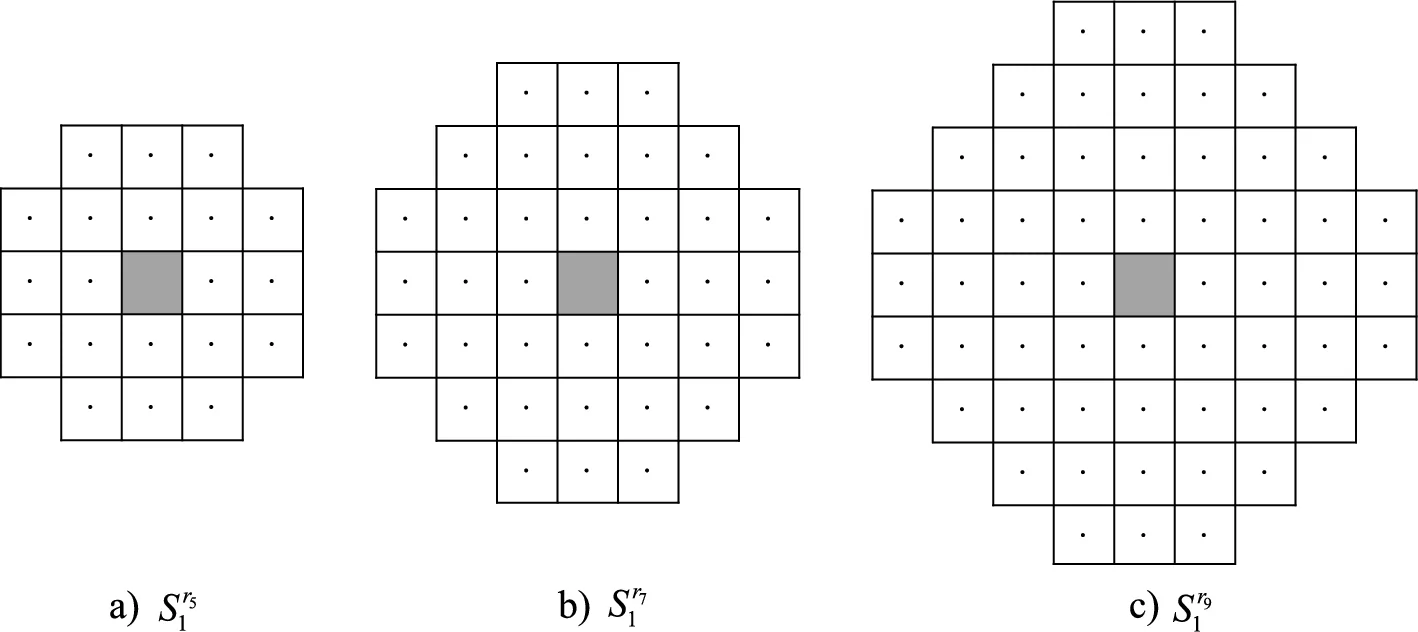
Higher order central stencils for the 2D-WENO interpolation. **a** Fifth order accurate central stencil, **b** seventh-order accurate central stencil, and **c** ninth order accurate central stencil. The (*i*,*j*)^th^ computational cell is denoted by the shaded region. The zone-centered point values of the neighboring zones are denoted by “centered-dot”

**Step 3) 2D Interpolation of the Three Electric Field Components; Electric Fields Input to 2D Riemann Solver** Recall that the zone-centered electric fields can always be obtained as various components of the zone-centered fluxes that were obtained in Step 2. Now that zone-centered pointwise values of the electric field, i.e., $e_{x;i,j,k}$, $e_{y;i,j,k}$, and $e_{z;i,j,k}$, have been evaluated, we make three 2D WENO interpolations of those variables in the *yz*-plane, the *xz*-plane, and the *xy*-plane, respectively. Specifically, within each zone, we interpolate $e_{x;i,j,k}$ in the *yz*-plane, we interpolate $e_{y;i,j,k}$ in the *xz*-plane, and we interpolate $e_{z;i,j,k}$ in the *xy*-plane. This enables us to obtain those same variables at edge-centered locations. (Such an efficient 2D WENO interpolation has been documented in Appendix A of this paper, at the 3rd, the 5th, and the 7th order, and higher order extensions, such as the ninth order, are easily made. Figures 5 and 6 show the kinds of stencils that are needed for the high-order interpolation.) The point of this interpolation is that it gives us the edge-centered point values of the electric field. At each edge, we have four abutting zones, and from each of those zones we will get one edge-aligned component of the electric field. Say we consider the *z*-edge given by $\left( {i + 1/2,j + 1/2,k} \right)$, as shown in Fig. 2. At that *z*-edge, we will have four different values of the *z*-component of the electric field. We have $e_{z;\mathrm{RU};i + 1/2,j + 1/2,k}$, which is interpolated with high-order accuracy from the “RU” zone relative to this edge. We have $e_{z;\mathrm{LU};i + 1/2,j + 1/2,k}$, which is interpolated with high-order accuracy from the “LU” zone relative to this edge. We have $e_{z;\mathrm{LD};i + 1/2,j + 1/2,k}$, which is interpolated with high-order accuracy from the “LD” zone relative to this edge. And we have $e_{z;\mathrm{RD};i + 1/2,j + 1/2,k}$, which is interpolated with high-order accuracy from the “RD” zone relative to this edge. Realize, therefore, from Eq. (12) that we have obtained everything that we need for evaluating the centered part of the electric field components in a pointwise fashion at the edge centers. This centered part is shown by the round bracket in Eq. (12). Realize that this averaged electric field is not yet stabilized, because we do not yet have the multidimensional dissipation term that is needed in the multidimensional Riemann solver. The process of obtaining the stabilizing terms, i.e., the square bracket in Eq. (12), will be described in the next point.

**Step 4) Reconstructed Magnetic Fields to Provide Inputs for the Dissipation Terms in the 2D Riemann Solver** Now focus on Fig. 2. Recall that the divergence-preserving condition ensures that the normal component of the magnetic field is continuous across the faces of the mesh. Therefore, along the *x* = constant surfaces of the mesh, we can use our 2D WENO reconstruction (from Step 1) of $\overline{B}_{x;i + 1/2,j + 1,k}$ in the face $\left( {i + 1/2,j + 1,k} \right)$ to obtain the point value $b_{x\mathrm{U};i + 1/2,j + 1/2,k}$ from above the black dot in Fig. 2. Similarly, we can use our 2D WENO reconstruction of $\overline{B}_{x;i + 1/2,j,k}$ in the face $\left( {i + 1/2,j,k} \right)$ to obtain the point value $b_{x\mathrm{D};i + 1/2,j + 1/2,k}$ from below the black dot in Fig. 2. Likewise, along the *y* = constant surfaces of the mesh, we can use our 2D WENO reconstruction (from Step 1) of $\overline{B}_{y;i + 1,j + 1/2,k}$ in the face $\left( {i + 1,j + 1/2,k} \right)$ to obtain the point value $b_{y\mathrm{R};i + 1/2,j + 1/2,k}$ from the right of the black dot in Fig. 2 and we can use another 2D WENO reconstruction of $\overline{B}_{y;i,j + 1/2,k}$ in the face $\left( {i,j + 1/2,k} \right)$ to obtain $b_{y\mathrm{L};i + 1/2,j + 1/2,k}$ from the left of the black dot in Fig. 2. When the solution is smooth, and when the interpolation is high order, we will have $b_{x\mathrm{U};i + 1/2,j + 1/2,k} \to b_{x\mathrm{D};i + 1/2,j + 1/2,k}$ and $b_{y\mathrm{R};i + 1/2,j + 1/2,k} \to b_{y\mathrm{L};i + 1/2,j + 1/2,k}$, with the result that the dissipation is significantly reduced. The end result is that at the edge centers of the mesh, we have the centered part of the *z*-component of the electric field, i.e., the round bracket in Eq. (12). We also have the dissipation term, i.e., the square bracket in Eq. (12).

**Step 5) Standard AFD-WENO Algorithm to Obtain Fluxes (or Fluctuations as Needed)** With the above items in hand, it is easy to see that the AFD-WENO algorithm from Balsara et al. [19] or Balsara et al. [20] can be used to obtain the time rate of change, $\left( {\partial_{t} {\mathbf{u}}} \right)_{i,j,k}$ for all the zone-centered variables. This is usually the most expensive step in the algorithm, because most PDEs have a large number of zone-centered primal variables. (This step usually becomes rather costly due to the fact that the first interpolation in this step has to be done in the characteristic variables.) Our use of the very economical AFD-WENO algorithm reduces the overall cost of the scheme.

**Step 6) Use Steps 1 and 3, Along with Speeds from Step 5 to Obtain Numerical Electric Field** Notice that in Step 1, the facial magnetic field components were reconstructed, and in Step 4, we showed how this reconstruction can be used to evaluate the numerical dissipation terms in the electric field. In Step 3, the electric fields were interpolated, so that we could obtain the centered part of the electric field at each edge. This enables us to obtain the four input electric fields that make up the centered part of the numerical electric field at the *z*-edge. The jumps in the magnetic field components at the *z*-edge also give us the numerical dissipation. Step 5 also gave us the speeds in all the directions at the *z*-edge. As a result, the pointwise, the stabilized electric field (that is useful for numerical schemes) can be written at the center of the *z*-edge as

23 $$\begin{aligned} e_{z;i + 1/2,j + 1/2,k}^{\mathrm{num}} & \equiv {{\left( {e_{z;\mathrm{RU};i + 1/2,j + 1/2,k} + e_{z;\mathrm{LU};i + 1/2,j + 1/2,k} + e_{z;\mathrm{LD};i + 1/2,j + 1/2,k} + e_{z;\mathrm{RD};i + 1/2,j + 1/2,k} } \right)} \mathord{\left/ {\vphantom {{\left( {e_{z;\mathrm{RU};i + 1/2,j + 1/2,k} + e_{z;\mathrm{LU};i + 1/2,j + 1/2,k} + e_{z;\mathrm{LD};i + 1/2,j + 1/2,k} + e_{z;\mathrm{RD};i + 1/2,j + 1/2,k} } \right)} 4}} \right. \kern-0pt} 4} \\& \quad + S{{\left[ {b_{x\mathrm{D};i + 1/2,j + 1/2,k} - b_{x\mathrm{U};i + 1/2,j + 1/2,k} + b_{y\mathrm{R};i + 1/2,j + 1/2,k} - b_{y\mathrm{L};i + 1/2,j + 1/2,k} } \right]} \mathord{\left/ {\vphantom {{\left[ {b_{x\mathrm{D};i + 1/2,j + 1/2,k} - b_{x\mathrm{U};i + 1/2,j + 1/2,k} + b_{y\mathrm{R};i + 1/2,j + 1/2,k} - b_{y\mathrm{L};i + 1/2,j + 1/2,k} } \right]} 2}} \right. \kern-0pt} 2}. \end{aligned}$$

This numerical electric field $e_{z;i + 1/2,j + 1/2,k}^{\mathrm{num}}$ is what we were seeking in this step and the previous step. Observe that this $e_{z;i + 1/2,j + 1/2,k}^{\mathrm{num}}$ is still a point value. However, it is a numerically stabilized point value. Similar edge-centered electric fields can be obtained at the *x*- and *y*-edges.

**Fig. 7:**
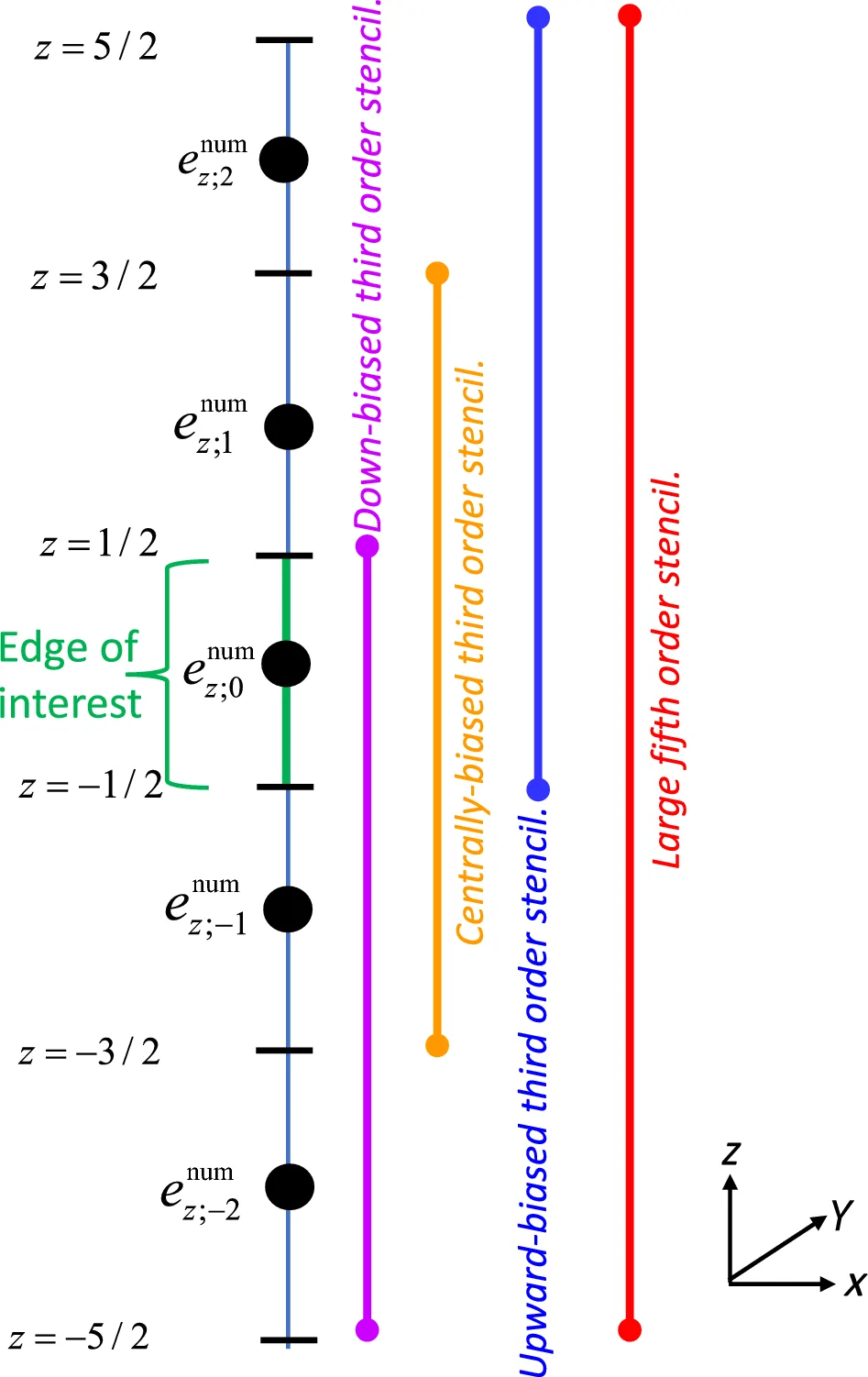
Part of the *z*-edge of the mesh. The points that make up the edge centers of the mesh are shown by the solid black dots. The high-order accurate pointwise values of $e_{z}^{\mathrm{num}}$ have been evaluated at the edge centers of the mesh using the 2D Riemann solver at each edge center. We want the high-order integral $\overline{E}_{z}^{\mathrm{num}}$ for values of “*z*” in the range [−1/2,1/2]. This edge of interest is also identified in green. The figure also shows the 1D stencils associated with the edge of interest for the third- and fifth-order WENO-AO interpolation schemes. We have three smaller third-order stencils and a large fifth-order stencil. We want a higher order line integral of $e_{z}^{\mathrm{num}}$ along the green edge of interest in the figure. Once a high order, 1D WENO interpolation polynomial has been evaluated using Legendre bases in the edge of interest, the leading term of that polynomial will give us the line integral with fifth order of accuracy. The interested reader should compare this figure with Fig. 1 from Balsara et al. [19] to realize that the solution to the problem described here is already available from Sect. 3 of the previously cited paper

**Step 7) From Point Values of the Electric Fields to Edge-Averaged Values** Notice that the update in Eqs. (3), (4), and (5) is based on an area-averaged interpretation of Eq. (1). Up to this point in the discussion, we only have point values of the numerically stabilized electric field components at the centers of each edge; see Fig. 7. Focusing on the *z*-component of the numerical electric field, we can now invoke pointwise 1D WENO interpolation along each *z*-edge. This pointwise 1D WENO interpolation algorithm has been described in Sect. 3 of Balsara et al. [19]. For instance, at the 5th order, the resulting interpolating polynomial can be written as

24 $$E_{z;i + 1/2,j + 1/2,k} \left( z \right) = \; \overline{E}_{z;i + 1/2,j + 1/2,k}^{\mathrm{num}} + e_{z} z + e_{zz} \left( {z^{2} - 1/12} \right) + e_{zzz} \left( {z^{3} - 3z/20} \right) + e_{zzzz} \left( {z^{2} - 3z^{2} /14 + 3/560} \right).$$

Note that because this is a 1D pointwise WENO interpolation, all the coefficients on the right-hand side of Eq. (24), including $\overline{E}_{z;i + 1/2,j + 1/2,k}^{\mathrm{num}}$, have to be evaluated. We can do the same operation along the *x*- and *y*-edges too. The term $\overline{E}_{z;i + 1/2,j + 1/2,k}^{\mathrm{num}}$ will naturally be the edge-averaged electric field along the *z*-edge. This is the variable that we seek in the update in Eqs. (3), (4), and (5). Note that we are using interpolation, not reconstruction; as a result, $\overline{E}_{z;i + 1/2,j + 1/2,k}^{\mathrm{num}}$ from Eq. (24) usually will not be the same as $e_{z;i + 1/2,j + 1/2,k}^{\mathrm{num}}$ from Eq. (23).

This completes our description of the scheme.

The scheme, as described here, can do several nice test problems and several scientific applications without further modification. For that reason, we have only described the baseline scheme here. However, there exist stringent MHD problems where one has very strong magnetic fields or very high-speed flows, resulting in very low plasma-*β*. Likewise, in several relativistic MHD problems, the presence of a very strong magnetic field results in very high magnetization of the plasma. We have also been able to extend the PCP property from our work in Bhoriya et al. [39] to encompass systems with a divergence-preserving constraint. This is described in the next section.

In this work, we have retained the zone-centered point values as the primal variables of our scheme. In some applications, it may be very useful to start with zone-centered point values and obtain a full FV reconstruction within a zone. This could be useful if additional physics needs to be specified within a zone, or if the zonal values have to be projected from one mesh to another mesh. In light of McCorquodale and Colella [68] and Buchmüller et al. [42], one might be given to believe that this can only be accomplished up to fourth order. This is not true. To drive this point home, Appendix B illustrates how the WENO formulation can be used to accomplish this at fifth order; but the procedure displayed in Appendix B can be carried out at all orders. One may also desire to obtain area-averaged fluxes from point values of fluxes at any order. In Appendix C, we again show that the WENO method can be used to do that up to fifth order and the method is extensible to all orders. Our goal in these appendices is to show that there might be a deeper connection between FV formulations and FD formulations at all orders and to illustrate how WENO methods can be used malleably to establish that connection.

## An Efficient and Time-Explicit Physical Constraint Preserving (PCP) Strategy for Divergence-Preserving PDEs

The PCP property is built around the notion that there exists a first-order scheme that preserves the PCP property. Therefore, whenever we are likely to lose physical realizability in a higher order scheme, we are willing to trade a decrease in accuracy in favor of physical realizability. A step-by-step implementation strategy for PCP in the numerical solution of conservation laws is given in Sect. 2 of Bhoriya et al. [39]. The core idea derives from Hu et al. [60]. Section 5 of Bhoriya et al. [39] also shows how a higher order in time SSP-RK update can be written as a linear combination of forward Euler steps. All those ideas are useful for the next two sub-sections.

### A First-Order PCP Scheme for Divergence-Free MHD

It is important to recall that all PCP methods for FD applications have in some way or form used the Hu et al. [60] idea of hybridization between a low-order scheme and a high-order scheme. However, the plan of carrying out such a hybridization for a divergence-constrained PDE is more intricate, because the facial magnetic field variables are indeed the primal variables. A first-order, zone-centered scheme for MHD that is updated with facial fluxes from an HLL or LLF Riemann solver will indeed be PCP, as was first proved by Gurski [55]. Such a scheme is very lightweight, because it does not entail any reconstruction or interpolation steps. The first, and most important, step is to obtain a PCP scheme for MHD that retains the facial magnetic fields as the primal variables.

For any first-order scheme, we can always average facial magnetic fields to obtain zone-centered magnetic fields. Because the facial magnetic fields are our primal magnetic fields, those fields will have to be updated using first-order accurate edge-centered electric fields, as described in Eqs. (18)–(20), or more simply in Eq. (12). The evaluation of the 2D Riemann solver at each edge is also very lightweight. The zone-centered magnetic field will then have to be obtained by arithmetic averaging of the facial magnetic field components to the zone center. Even for the most stringent of problems, such a scheme will remain PCP in most of the zones. There will be a rare few zones where the averaging of the facial magnetic fields to the zone centers will indeed destroy the PCP property. To get a bullet-proof scheme, we have to find a way to cure this problem. (Documenting such a low-order PCP scheme is the task of this sub-section. Describing how it is hybridized with a higher order scheme that is not PCP is the task of the next sub-section.)

Let us explain the previous paragraph in more mathematical terms, so that the reader can appreciate the issue that we discuss here. The discussion here will use a first order in time forward Euler approach, because a higher order in time can be achieved using an SSP-RK method that combines forward Euler methods. We start with a first-order version of Eq. (2) to obtain ${\mathbf{u}}_{i,j,k}^{n;\mathrm{LO}}$. (The superscript “LO” stands for low order; the superscript “HO” stands for high order.) However, for a divergence-preserving scheme with facial magnetic fields as the primal variables, the sixth, seventh, and eighth components of ${\mathbf{u}}_{i,j,k}^{n;\mathrm{LO}}$ will be replaced by ${{\left( {\overline{B}_{x;i + 1/2,j,k}^{n;\mathrm{LO}} + \overline{B}_{x;i - 1/2,j,k}^{n;\mathrm{LO}} } \right)} \mathord{\left/ {\vphantom {{\left( {\overline{B}_{x;i + 1/2,j,k}^{n;\mathrm{LO}} + \overline{B}_{x;i - 1/2,j,k}^{n;\mathrm{LO}} } \right)} 2}} \right. \kern-0pt} 2}$, ${{\left( {\overline{B}_{y;i,j + 1/2,k}^{n;\mathrm{LO}} + \overline{B}_{y;i,j - 1/2,k}^{n;\mathrm{LO}} } \right)} \mathord{\left/ {\vphantom {{\left( {\overline{B}_{y;i,j + 1/2,k}^{n;\mathrm{LO}} + \overline{B}_{y;i,j - 1/2,k}^{n;\mathrm{LO}} } \right)} 2}} \right. \kern-0pt} 2}$, and ${{\left( {\overline{B}_{z;i,j,k + 1/2}^{n;\mathrm{LO}} + \overline{B}_{z;i,j,k - 1/2}^{n;\mathrm{LO}} } \right)} \mathord{\left/ {\vphantom {{\left( {\overline{B}_{z;i,j,k + 1/2}^{n;\mathrm{LO}} + \overline{B}_{z;i,j,k - 1/2}^{n;\mathrm{LO}} } \right)} 2}} \right. \kern-0pt} 2}$, respectively. We start by assuming that ${\mathbf{u}}_{i,j,k}^{n;\mathrm{LO}}$ is PCP. We then make the forward Euler updates

25 $${\tilde{\mathbf{u}}}_{i,j,k}^{n + 1;\mathrm{LO}} = {\mathbf{u}}_{i,j,k}^{n;\mathrm{LO}} - \frac{\Delta t}{{\Delta x}}\left( {{\mathbf{f}}_{i + 1/2,j,k}^{\mathrm{LO}} - {\mathbf{f}}_{i - 1/2,j,k}^{\mathrm{LO}} } \right) - \frac{\Delta t}{{\Delta y}}\left( {{\mathbf{g}}_{i,j + 1/2,k}^{\mathrm{LO}} - {\mathbf{g}}_{i,j - 1/2,k}^{\mathrm{LO}} } \right) - \frac{\Delta t}{{\Delta z}}\left( {{\mathbf{h}}_{i,j,k + 1/2}^{\mathrm{LO}} - {\mathbf{h}}_{i,j,k - 1/2}^{\mathrm{LO}} } \right);$$

26 $$\overline{B}_{x; \, i + 1/2,j,k}^{n + 1;\mathrm{LO}} = \overline{B}_{x; \, i + 1/2,j,k}^{n;\mathrm{LO}} \, - \frac{\Delta t}{{\Delta y\Delta z}}\left( {\Delta z\overline{E}_{z; \, i + 1/2,j + 1/2,k}^{\mathrm{LO}} - \Delta z\overline{E}_{z; \, i + 1/2,j - 1/2,k}^{\mathrm{LO}} + \Delta y\overline{E}_{y; \, i + 1/2,j,k - 1/2}^{\mathrm{LO}} - \Delta y\overline{E}_{y; \, i + 1/2,j,k + 1/2}^{\mathrm{LO}} } \right);$$

27 $$\overline{B}_{y; \, i,j - 1/2,k}^{n + 1;\mathrm{LO}} { = }\overline{B}_{y; \, i,j - 1/2,k}^{n;\mathrm{LO}} \, - \frac{\Delta t}{{\Delta x\Delta z}}\left( {\Delta x\overline{E}_{x; \, i,j - 1/2,k + 1/2}^{\mathrm{LO}} - \Delta x\overline{E}_{x; \, i,j - 1/2,k - 1/2}^{\mathrm{LO}} + \Delta z\overline{E}_{z; \, i - 1/2,j - 1/2,k}^{\mathrm{LO}} - \Delta z\overline{E}_{z; \, i + 1/2,j - 1/2,k}^{\mathrm{LO}} } \right);$$

28 $$\overline{B}_{z; \, i,j,k + 1/2}^{n + 1;\mathrm{LO}} = \overline{B}_{z; \, i,j,k + 1/2}^{n;\mathrm{LO}} - \frac{\Delta t}{{\Delta x\Delta y}}\left( {\Delta x\overline{E}_{x; \, i,j - 1/2,k + 1/2}^{\mathrm{LO}} - \Delta x\overline{E}_{x; \, i,j + 1/2,k + 1/2}^{\mathrm{LO}} + \Delta y\overline{E}_{y; \, i + 1/2,j,k + 1/2}^{\mathrm{LO}} - \Delta y\overline{E}_{y; \, i - 1/2,j,k + 1/2}^{\mathrm{LO}} } \right).$$

Now, realize that because ${\mathbf{u}}_{i,j,k}^{n;\mathrm{LO}}$ started off PCP, and because Eq. (25) is a first-order update with a PCP-preserving HLL or LLF flux, we are guaranteed that the updated zone-centered variable ${\tilde{\mathbf{u}}}_{i,j,k}^{n + 1;\mathrm{LO}}$ will be PCP. It is only when the sixth, seventh, and eighth components of ${\tilde{\mathbf{u}}}_{i,j,k}^{n + 1;\mathrm{LO}}$ are replaced with ${{\left( {\overline{B}_{x;i + 1/2,j,k}^{n + 1;\mathrm{LO}} + \overline{B}_{x;i - 1/2,j,k}^{n + 1;\mathrm{LO}} } \right)} \mathord{\left/ {\vphantom {{\left( {\overline{B}_{x;i + 1/2,j,k}^{n + 1;\mathrm{LO}} + \overline{B}_{x;i - 1/2,j,k}^{n + 1;\mathrm{LO}} } \right)} 2}} \right. \kern-0pt} 2}$, ${{\left( {\overline{B}_{y;i,j + 1/2,k}^{n + 1;\mathrm{LO}} + \overline{B}_{y;i,j - 1/2,k}^{n + 1;\mathrm{LO}} } \right)} \mathord{\left/ {\vphantom {{\left( {\overline{B}_{y;i,j + 1/2,k}^{n + 1;\mathrm{LO}} + \overline{B}_{y;i,j - 1/2,k}^{n + 1;\mathrm{LO}} } \right)} 2}} \right. \kern-0pt} 2}$, and ${{\left( {\overline{B}_{z;i,j,k + 1/2}^{n + 1;\mathrm{LO}} + \overline{B}_{z;i,j,k - 1/2}^{n + 1;\mathrm{LO}} } \right)} \mathord{\left/ {\vphantom {{\left( {\overline{B}_{z;i,j,k + 1/2}^{n + 1;\mathrm{LO}} + \overline{B}_{z;i,j,k - 1/2}^{n + 1;\mathrm{LO}} } \right)} 2}} \right. \kern-0pt} 2}$ from Eqs. (26), (27), and (28) that we have the possibility of losing the PCP property in a few rare zones! After the replacement of the sixth, seventh, and eighth components of ${\tilde{\mathbf{u}}}_{i,j,k}^{n + 1;\mathrm{LO}}$ by the facial averages, let us call the zone-centered variables ${\mathbf{u}}_{i,j,k}^{n + 1;\mathrm{LO}}$. Therefore, it is this replacement by the facial averages that may, in a few rare zones, turn ${\tilde{\mathbf{u}}}_{i,j,k}^{n + 1;\mathrm{LO}}$ (which is PCP) into ${\mathbf{u}}_{i,j,k}^{n + 1;\mathrm{LO}}$ (which may not be PCP). Our task is to fix this situation.

Two clear fixes are obvious. The first fix draws upon work by Abgrall et al. [1, 2]. Realize that when ${\mathbf{u}}_{i,j,k}^{n + 1;\mathrm{LO}}$ loses the PCP property, it does so because of the slightest discretization error, i.e., the facial fluxes that were used to update the zone-centered energy did not bring in enough energy from neighboring zones to keep ${\mathbf{u}}_{i,j,k}^{n + 1;\mathrm{LO}}$ PCP. Let $\left[ {\mathbf{X}} \right]_{5}$ denote the fifth component of a vector ${\mathbf{X}}$; this is just a choice of notation. We can ever so slightly modify the fifth component of the flux for the MHD case as follows:

29 $$\begin{aligned} \left\{ \begin{aligned} & \left[ {{\tilde{\mathbf{f}}}_{i + 1/2,j,k}^{\mathrm{LO}} } \right]_{5} = \left[ {{\mathbf{f}}_{i + 1/2,j,k}^{\mathrm{LO}} } \right]_{5} - \overline{w}_{i + 1/2,j,k}^{ - } \alpha { ; }\left[ {{\tilde{\mathbf{f}}}_{i - 1/2,j,k}^{\mathrm{LO}} } \right]_{5} = \left[ {{\mathbf{f}}_{i - 1/2,j,k}^{\mathrm{LO}} } \right]_{5} + \overline{w}_{i - 1/2,j,k}^{ + } \alpha { ;} \\& \left[ {{\tilde{\mathbf{g}}}_{i,j + 1/2,k}^{\mathrm{LO}} } \right]_{5} = \left[ {{\mathbf{g}}_{i,j + 1/2,k}^{\mathrm{LO}} } \right]_{5} - \overline{w}_{i,j + 1/2,k}^{ - } \alpha { ; }\left[ {{\tilde{\mathbf{g}}}_{i,j - 1/2,k}^{\mathrm{LO}} } \right]_{5} = \left[ {{\mathbf{g}}_{i,j - 1/2,k}^{\mathrm{LO}} } \right]_{5} + \overline{w}_{i,j - 1/2,k}^{ + } \alpha { ;} \\& \left[ {{\tilde{\mathbf{h}}}_{i,j,k + 1/2}^{\mathrm{LO}} } \right]_{5} = \left[ {{\mathbf{h}}_{i,j,k + 1/2}^{\mathrm{LO}} } \right]_{5} - \overline{w}_{i,j,k + 1/2}^{ - } \alpha { ; }\left[ {{\tilde{\mathbf{h}}}_{i,j,k - 1/2}^{\mathrm{LO}} } \right]_{5} = \left[ {{\mathbf{h}}_{i,j,k - 1/2}^{\mathrm{LO}} } \right]_{5} + \overline{w}_{i,j,k - 1/2}^{ + } \alpha . \end{aligned} \right. \end{aligned}$$

The $\overline{w}$ variables will be described shortly and they are just a measure of a neighboring zone’s ability to give enough energy to the troubled zone so that the troubled zone has a positive pressure. We can then quantify the variable “α” in the above equation by setting it using the following equation:

30 $$\begin{gathered} \frac{1}{8\uppi }\left\{ \begin{gathered} \left( {{{\left( {\overline{B}_{x;i + 1/2,j,k}^{n + 1;\mathrm{LO}} + \overline{B}_{x;i - 1/2,j,k}^{n + 1;\mathrm{LO}} } \right)} \mathord{\left/ {\vphantom {{\left( {\overline{B}_{x;i + 1/2,j,k}^{n + 1;\mathrm{LO}} + \overline{B}_{x;i - 1/2,j,k}^{n + 1;\mathrm{LO}} } \right)} 2}} \right. \kern-0pt} 2}} \right)^{2} + \left( {{{\left( {\overline{B}_{y;i,j + 1/2,k}^{n + 1;\mathrm{LO}} + \overline{B}_{y;i,j - 1/2,k}^{n + 1;\mathrm{LO}} } \right)} \mathord{\left/ {\vphantom {{\left( {\overline{B}_{y;i,j + 1/2,k}^{n + 1;\mathrm{LO}} + \overline{B}_{y;i,j - 1/2,k}^{n + 1;\mathrm{LO}} } \right)} 2}} \right. \kern-0pt} 2}} \right)^{2} \hfill \\ + \left( {{{\left( {\overline{B}_{z;i,j,k + 1/2}^{n + 1;\mathrm{LO}} + \overline{B}_{z;i,j,k - 1/2}^{n + 1;\mathrm{LO}} } \right)} \mathord{\left/ {\vphantom {{\left( {\overline{B}_{z;i,j,k + 1/2}^{n + 1;\mathrm{LO}} + \overline{B}_{z;i,j,k - 1/2}^{n + 1;\mathrm{LO}} } \right)} 2}} \right. \kern-0pt} 2}} \right)^{2} - \left( {\left[ {{\tilde{\mathbf{u}}}_{i,j,k}^{n + 1;\mathrm{LO}} } \right]_{6} } \right)^{2} - \left( {\left[ {{\tilde{\mathbf{u}}}_{i,j,k}^{n + 1;\mathrm{LO}} } \right]_{7} } \right)^{2} - \left( {\left[ {{\tilde{\mathbf{u}}}_{i,j,k}^{n + 1;\mathrm{LO}} } \right]_{8} } \right)^{2} \hfill \\ \end{gathered} \right\} \hfill \\ \, = \alpha \left\{ {\frac{\Delta t}{{\Delta x}}\left( {\overline{w}_{i + 1/2,j,k}^{ - } + \overline{w}_{i - 1/2,j,k}^{ + } } \right) + \frac{\Delta t}{{\Delta y}}\left( {\overline{w}_{i,j + 1/2,k}^{ - } + \overline{w}_{i,j - 1/2,k}^{ + } } \right) + \frac{\Delta t}{{\Delta z}}\left( {\overline{w}_{i,j,k + 1/2}^{ - } + \overline{w}_{i,j,k - 1/2}^{ + } } \right)} \right\}. \hfill \\ \end{gathered}$$

Then, we can set

31 $$\begin{aligned} \left\{ \begin{aligned} & w_{i + 1/2,j,k}^{ - } { = }\left( {\max \left( {P_{i + 1,j,k}^{n;\mathrm{LO}} - P_{\min } ,0} \right)} \right)^{2} { ; }w_{i - 1/2,j,k}^{ + } { = }\left( {\max \left( {P_{i - 1,j,k}^{n;\mathrm{LO}} - P_{\min } ,0} \right)} \right)^{2} { ; }w_{i,j + 1/2,k}^{ - } { = }\left( {\max \left( {P_{i,j + 1,k}^{n;\mathrm{LO}} - P_{\min } ,0} \right)} \right)^{2} { ; } \\& w_{i,j - 1/2,k}^{ + } { = }\left( {\max \left( {P_{i,j - 1,k}^{n;\mathrm{LO}} - P_{\min } ,0} \right)} \right)^{2} { ; }w_{i,j,k + 1/2}^{ - } { = }\left( {\max \left( {P_{i,j,k + 1}^{n;\mathrm{LO}} - P_{\min } ,0} \right)} \right)^{2} { ; }w_{i,j,k - 1/2}^{ + } { = }\left( {\max \left( {P_{i,j,k - 1}^{n;\mathrm{LO}} - P_{\min } ,0} \right)} \right)^{2} . \end{aligned} \right. \end{aligned}$$

Here, $P_{\min }$ is a user-settable small parameter. (We have set $P_{\min } = 10^{ - 3}$ for all the tests reported here.) The weights with overbars can then be normalized, so that they add to unity in a WENO-like normalization that goes as follows:

32 $$\begin{aligned} \left\{ \begin{aligned} & \overline{w}_{i + 1/2,j,k}^{ - } { = }{{w_{i + 1/2,j,k}^{ - } } \mathord{\left/ {\vphantom {{w_{i + 1/2,j,k}^{ - } } {\left( {w_{i + 1/2,j,k}^{ - } + w_{i - 1/2,j,k}^{ + } + w_{i,j + 1/2,k}^{ - } + w_{i,j - 1/2,k}^{ + } + w_{i,j,k + 1/2}^{ - } + w_{i,j,k - 1/2}^{ + } } \right)}}} \right. \kern-0pt} {\left( {w_{i + 1/2,j,k}^{ - } + w_{i - 1/2,j,k}^{ + } + w_{i,j + 1/2,k}^{ - } + w_{i,j - 1/2,k}^{ + } + w_{i,j,k + 1/2}^{ - } + w_{i,j,k - 1/2}^{ + } } \right)}}; \\& \overline{w}_{i - 1/2,j,k}^{ + } = {{w_{i - 1/2,j,k}^{ + } } \mathord{\left/ {\vphantom {{w_{i - 1/2,j,k}^{ + } } {\left( {w_{i + 1/2,j,k}^{ - } + w_{i - 1/2,j,k}^{ + } + w_{i,j + 1/2,k}^{ - } + w_{i,j - 1/2,k}^{ + } + w_{i,j,k + 1/2}^{ - } + w_{i,j,k - 1/2}^{ + } } \right)}}} \right. \kern-0pt} {\left( {w_{i + 1/2,j,k}^{ - } + w_{i - 1/2,j,k}^{ + } + w_{i,j + 1/2,k}^{ - } + w_{i,j - 1/2,k}^{ + } + w_{i,j,k + 1/2}^{ - } + w_{i,j,k - 1/2}^{ + } } \right)}}; \\& \overline{w}_{i,j + 1/2,k}^{ - } = {{w_{i,j + 1/2,k}^{ - } } \mathord{\left/ {\vphantom {{w_{i,j + 1/2,k}^{ - } } {\left( {w_{i + 1/2,j,k}^{ - } + w_{i - 1/2,j,k}^{ + } + w_{i,j + 1/2,k}^{ - } + w_{i,j - 1/2,k}^{ + } + w_{i,j,k + 1/2}^{ - } + w_{i,j,k - 1/2}^{ + } } \right)}}} \right. \kern-0pt} {\left( {w_{i + 1/2,j,k}^{ - } + w_{i - 1/2,j,k}^{ + } + w_{i,j + 1/2,k}^{ - } + w_{i,j - 1/2,k}^{ + } + w_{i,j,k + 1/2}^{ - } + w_{i,j,k - 1/2}^{ + } } \right)}}; \\& \overline{w}_{i,j - 1/2,k}^{ + } = {{w_{i,j - 1/2,k}^{ + } } \mathord{\left/ {\vphantom {{w_{i,j - 1/2,k}^{ + } } {\left( {w_{i + 1/2,j,k}^{ - } + w_{i - 1/2,j,k}^{ + } + w_{i,j + 1/2,k}^{ - } + w_{i,j - 1/2,k}^{ + } + w_{i,j,k + 1/2}^{ - } + w_{i,j,k - 1/2}^{ + } } \right)}}} \right. \kern-0pt} {\left( {w_{i + 1/2,j,k}^{ - } + w_{i - 1/2,j,k}^{ + } + w_{i,j + 1/2,k}^{ - } + w_{i,j - 1/2,k}^{ + } + w_{i,j,k + 1/2}^{ - } + w_{i,j,k - 1/2}^{ + } } \right)}}; \\& \overline{w}_{i,j,k + 1/2}^{ - } = {{w_{i,j,k + 1/2}^{ - } } \mathord{\left/ {\vphantom {{w_{i,j,k + 1/2}^{ - } } {\left( {w_{i + 1/2,j,k}^{ - } + w_{i - 1/2,j,k}^{ + } + w_{i,j + 1/2,k}^{ - } + w_{i,j - 1/2,k}^{ + } + w_{i,j,k + 1/2}^{ - } + w_{i,j,k - 1/2}^{ + } } \right)}}} \right. \kern-0pt} {\left( {w_{i + 1/2,j,k}^{ - } + w_{i - 1/2,j,k}^{ + } + w_{i,j + 1/2,k}^{ - } + w_{i,j - 1/2,k}^{ + } + w_{i,j,k + 1/2}^{ - } + w_{i,j,k - 1/2}^{ + } } \right)}}; \\& \overline{w}_{i,j,k - 1/2}^{ + } = {{w_{i,j,k - 1/2}^{ + } } \mathord{\left/ {\vphantom {{w_{i,j,k - 1/2}^{ + } } {\left( {w_{i + 1/2,j,k}^{ - } + w_{i - 1/2,j,k}^{ + } + w_{i,j + 1/2,k}^{ - } + w_{i,j - 1/2,k}^{ + } + w_{i,j,k + 1/2}^{ - } + w_{i,j,k - 1/2}^{ + } } \right)}}} \right. \kern-0pt} {\left( {w_{i + 1/2,j,k}^{ - } + w_{i - 1/2,j,k}^{ + } + w_{i,j + 1/2,k}^{ - } + w_{i,j - 1/2,k}^{ + } + w_{i,j,k + 1/2}^{ - } + w_{i,j,k - 1/2}^{ + } } \right)}}. \end{aligned} \right. \end{aligned}$$

As long as a troubled zone has at least one neighboring zone that can lend it some thermal energy, it will be able to obtain some extra energy to restore its positive pressure. This is a fully conservative fix.

It is also possible that a zone may not have any von Neumann neighbors that have sufficient energy to lend it some thermal energy. It is only on those rare occasions that we take a different approach. Realize that $\tilde{u}_{i,j,k}^{n + 1;\mathrm{LO}}$ is still PCP, so it still has positive pressure. Therefore, we already have a pressure in the troubled zone that is positive, except that it was obtained via the update in Eq. (25). We can reset the troubled zone to have that same positive pressure as follows:

33 $$\begin{gathered} \left[ {{\mathbf{u}}_{i,j,k}^{n + 1;\mathrm{LO}} } \right]_{5} = \left[ {{\tilde{\mathbf{u}}}_{i,j,k}^{n + 1;\mathrm{LO}} } \right]_{5} + \left[ {{\mathbf{s}}_{i,j,k}^{\mathrm{LO}} } \right]_{5} {\text{ with the definition}} \hfill \\ \left[ {{\mathbf{s}}_{i,j,k}^{\mathrm{LO}} } \right]_{5} \equiv \frac{1}{8\uppi }\left\{ \begin{gathered} \left( {{{\left( {\overline{B}_{x;i + 1/2,j,k}^{n + 1;\mathrm{LO}} + \overline{B}_{x;i - 1/2,j,k}^{n + 1;\mathrm{LO}} } \right)} \mathord{\left/ {\vphantom {{\left( {\overline{B}_{x;i + 1/2,j,k}^{n + 1;\mathrm{LO}} + \overline{B}_{x;i - 1/2,j,k}^{n + 1;\mathrm{LO}} } \right)} 2}} \right. \kern-0pt} 2}} \right)^{2} + \left( {{{\left( {\overline{B}_{y;i,j + 1/2,k}^{n + 1;\mathrm{LO}} + \overline{B}_{y;i,j - 1/2,k}^{n + 1;\mathrm{LO}} } \right)} \mathord{\left/ {\vphantom {{\left( {\overline{B}_{y;i,j + 1/2,k}^{n + 1;\mathrm{LO}} + \overline{B}_{y;i,j - 1/2,k}^{n + 1;\mathrm{LO}} } \right)} 2}} \right. \kern-0pt} 2}} \right)^{2} \hfill \\ + \left( {{{\left( {\overline{B}_{z;i,j,k + 1/2}^{n + 1;\mathrm{LO}} + \overline{B}_{z;i,j,k - 1/2}^{n + 1;\mathrm{LO}} } \right)} \mathord{\left/ {\vphantom {{\left( {\overline{B}_{z;i,j,k + 1/2}^{n + 1;\mathrm{LO}} + \overline{B}_{z;i,j,k - 1/2}^{n + 1;\mathrm{LO}} } \right)} 2}} \right. \kern-0pt} 2}} \right)^{2} - \left( {\left[ {\tilde{u}_{i,j,k}^{n + 1;\mathrm{LO}} } \right]_{6} } \right)^{2} - \left( {\left[ {\tilde{u}_{i,j,k}^{n + 1;\mathrm{LO}} } \right]_{7} } \right)^{2} - \left( {\left[ {\tilde{u}_{i,j,k}^{n + 1;\mathrm{LO}} } \right]_{8} } \right)^{2} \hfill \\ \end{gathered} \right\}. \hfill \\ \end{gathered}$$

The low-order source term $\left[ {{\mathbf{s}}_{i,j,k}^{\mathrm{LO}} } \right]_{5}$ contributes only to the fifth component, which is the energy equation, for MHD. Only the fifth component of the source term vector ${\mathbf{s}}_{i,j,k}^{\mathrm{LO}}$ is non-zero and that too only for zones that have lost the PCP property in the first-order update. The really big advantage of this lower order formulation is that we always have a PCP formulation that is divergence-free which can be used to guide the higher order scheme, so that it always remains divergence-free and PCP. In the next sub-section, we will show how we do this in the most unobtrusive of ways, so that for the most part, we only use the high-order scheme, only resorting to the lower order scheme from this sub-section in zones where the PCP property may be lost.

### Hybridizing the First-Order PCP Scheme with the Higher Order Scheme

In each zone $\left( {i,j,k} \right)$, we define a variable $\theta_{i,j,k}$. Following the philosophy of Hu et al. [60], we will design a method such that when $\theta_{i,j,k} = 1$ for all the zones, the scheme will exclusively be a high-order scheme; this is the default when the MHD problem is not too stringent. When $\theta_{i,j,k} = 0$ for all the zones, the scheme will exclusively be a first-order scheme. Of course, it is not our intent/desire to have $\theta_{i,j,k} = 0$ in any of the zones, but for stringent problems, we might have $0 \leqslant \theta_{i,j,k} < 1$ in some of the zones. At each zone boundary, we can define a flux by

34 $$\begin{aligned} \left\{ \begin{aligned} & {\mathbf{f}}_{i + 1/2,j,k}^{\theta } = \left( {1 - \theta_{i + 1/2,j,k}^{\mathrm{face}} } \right){\tilde{\mathbf{f}}}_{i + 1/2,j,k}^{\mathrm{LO}} + \theta_{i + 1/2,j,k}^{\mathrm{face}} {\mathbf{f}}_{i + 1/2,j,k}^{\mathrm{HO}} {\text{ with }}\theta_{i + 1/2,j,k}^\mathrm{face} \equiv \min \left( {\theta_{i,j,k} ,\theta_{i + 1,j,k} } \right){ ;} \\& {\mathbf{g}}_{i,j + 1/2,k}^{\theta } = \left( {1 - \theta_{i,j + 1/2,k}^{\mathrm{face}} } \right){\tilde{\mathbf{g}}}_{i,j + 1/2,k}^{\mathrm{LO}} + \theta_{i,j + 1/2,k}^{\mathrm{face}} {\mathbf{g}}_{i,j + 1/2,k}^{\mathrm{HO}} {\text{ with }}\theta_{i,j + 1/2,k}^{\mathrm{face}} \equiv \min \left( {\theta_{i,j,k} ,\theta_{i,j + 1,k} } \right){ ;} \\& {\mathbf{h}}_{i,j,k + 1/2}^{\theta } = \left( {1 - \theta_{i,j,k + 1/2}^{\mathrm{face}} } \right){\tilde{\mathbf{h}}}_{i,j,k + 1/2}^{\mathrm{LO}} + \theta_{i,j,k + 1/2}^{\mathrm{face}} {\mathbf{h}}_{i,j,k + 1/2}^{\mathrm{HO}} {\text{ with }}\theta_{i,j,k + 1/2}^{\mathrm{face}} \equiv \min \left( {\theta_{i,j,k} ,\theta_{i,j,k + 1} } \right). \end{aligned} \right. \end{aligned}$$

**Fig. 8:**
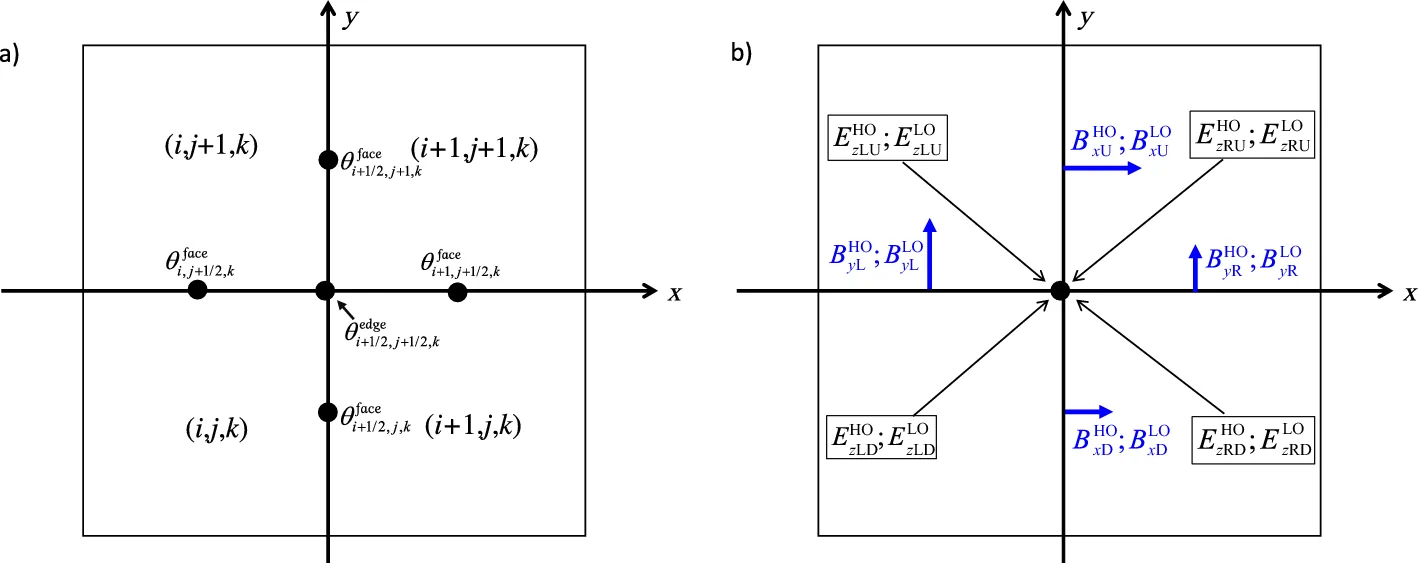
Are analogous to Fig. 2, because they show four zones in the *xy*-plane that come together at the *z*-edge of a 3D mesh. **a** Four zones that surround a *z*-edge. It shows how the “*θ*” variables that are evaluated at the *xz*- and *yz*-faces can be used to form an effective “*θ*” at the *z*-edge of the mesh. This effective “*θ*” at the *z*-edge can then be used to lower the order of the edge-centered *z*-component of the electric field that is used in the update of the facial magnetic fields in the *xz*- and *yz*-faces. Like Fig. 2, **b** shows the inputs that go into the evaluation of the *z*-component of the electric field. The only difference from Fig. 2 is that we now have the option of making a high-order evaluation (which uses all the high-order WENO reconstructions and interpolations as described in the text) which is superscripted with “HO”; a low-order (first-order) evaluation which is superscripted with “LO”

The left panel of Fig. 8 shows how these facial values of θ are collocated. Likewise, at each edge, we can define

35 $$\begin{aligned} \left\{ \begin{aligned} & \overline{E}_{z;i + 1/2,j + 1/2,k}^{\theta } = \left( {1 - \theta_{i + 1/2,j + 1/2,k}^{\mathrm{edge}} } \right)\overline{E}_{z;i + 1/2,j + 1/2,k}^{\mathrm{LO}} \, + \theta_{i + 1/2,j + 1/2,k}^{\mathrm{edge}} \overline{E}_{z;i + 1/2,j + 1/2,k}^{\mathrm{HO}} \\& {\text{ with }}\theta_{i + 1/2,j + 1/2,k}^{\mathrm{edge}} \equiv \min \left( {\theta_{i,j,k} ,\theta_{i + 1,j,k} ,\theta_{i,j + 1,k} ,\theta_{i + 1,j + 1,k} } \right){ ;} \\& \overline{E}_{y;i + 1/2,j,k + 1/2}^{\theta } = \left( {1 - \theta_{i + 1/2,j,k + 1/2}^{\mathrm{edge}} } \right)\overline{E}_{y;i + 1/2,j,k + 1/2}^{\mathrm{LO}} + \theta_{i + 1/2,j,k + 1/2}^{\mathrm{edge}} \overline{E}_{y;i + 1/2,j,k + 1/2}^{\mathrm{HO}} \, \\& {\text{ with }}\theta_{i + 1/2,j,k + 1/2}^{\mathrm{edge}} \equiv \min \left( {\theta_{i,j,k} ,\theta_{i + 1,j,k} ,\theta_{i,j,k + 1} ,\theta_{i + 1,j,k + 1} } \right){ ;} \\& \overline{E}_{x;i,j + 1/2,k + 1/2}^{\theta } = \left( {1 - \theta_{i,j + 1/2,k + 1/2}^{\mathrm{edge}} } \right)\overline{E}_{x;i,j + 1/2,k + 1/2}^{\mathrm{LO}} + \theta_{i,j + 1/2,k + 1/2}^{\mathrm{edge}} \overline{E}_{x;i,j + 1/2,k + 1/2}^{\mathrm{HO}} \, \\& {\text{ with }}\theta_{i,j + 1/2,k + 1/2}^{\mathrm{edge}} \equiv \min \left( {\theta_{i,j,k} ,\theta_{i,j + 1,k} ,\theta_{i,j,k + 1} ,\theta_{i,j + 1,k + 1} } \right). \end{aligned} \right. \end{aligned}$$

**Fig. 9:**
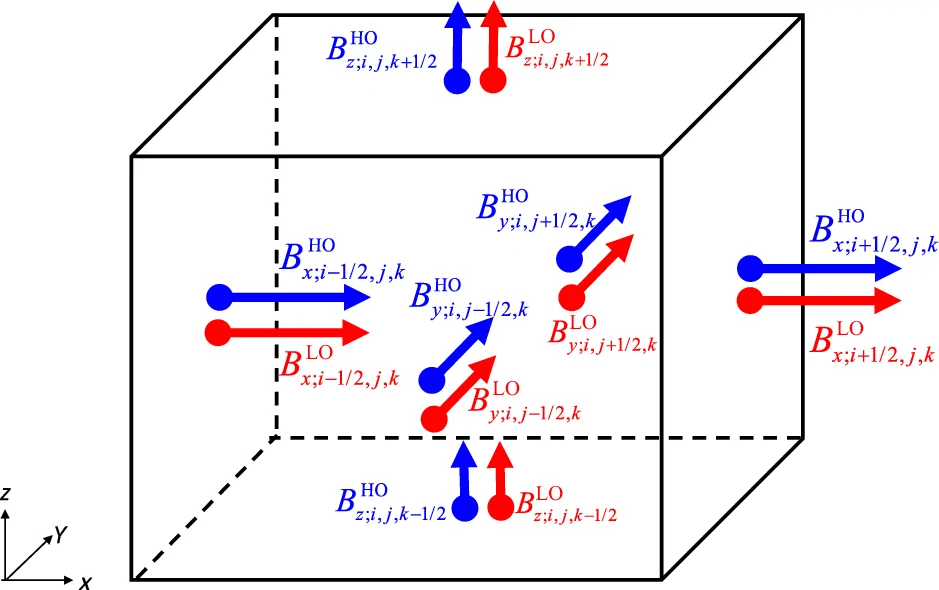
This figure is analogous to Fig. 1, because it shows the components of the magnetic field in the faces of the mesh. The difference from Fig. 1 is that within each face we now have a high-order component which is superscripted with “HO”; a low-order component which is superscripted with “LO”. Both components in each face have been advanced in time using a forward Euler scheme with a timestep Δ*t*. This temporal advance of the facial magnetic field components is made before a call to the AFD-WENO routine, and both the components within each face will be used for the PCP update

The left panel of Fig. 9 shows how these edge values of θ are collocated. The right panel of Fig. 9 shows how these can be used to obtain electric fields at the edges. The above two equations show that the order of accuracy of the fluxes and electric fields can be locally lowered, as needed, to the point where the first-order scheme always guarantees PCP behavior.

We now describe an iterative update strategy that does just that. We iterate over the whole mesh, starting the iteration process with $\theta_{i,j,k} = 1$ for all the zones. This $\theta_{i,j,k}$ will be sequentially lowered for any zone that is troubled, but realize that it will only be lowered for the few zones that are troubled. For each iteration, Eqs. (2) to (5) can be modified to become

36 $$\begin{aligned} \partial_{t} {\mathbf{u}}_{i,j,k}^{\theta } =& - \frac{1}{\Delta x}\left( {{\mathbf{f}}_{i + 1/2,j,k}^{\theta } - {\mathbf{f}}_{i - 1/2,j,k}^{\theta } } \right) - \frac{1}{\Delta y}\left( {{\mathbf{g}}_{i,j + 1/2,k}^{\theta } - {\mathbf{g}}_{i,j - 1/2,k}^{\theta } } \right) - \frac{1}{\Delta z}\left( {{\mathbf{h}}_{i,j,k + 1/2}^{\theta } - {\mathbf{h}}_{i,j,k - 1/2}^{\theta } } \right) \\ & \, \, { + }\frac{1}{\Delta t}\left( {1 - \theta_{i,j,k} } \right){\mathbf{s}}_{i,j,k}^{\mathrm{LO}}; \end{aligned}$$

37 $$\partial_{t} \overline{B}_{x; \, i + 1/2,j,k}^{\theta } { = } - \frac{1}{\Delta y\Delta z}\left( {\Delta z\overline{E}_{z; \, i + 1/2,j + 1/2,k}^{\theta } - \Delta z\overline{E}_{z; \, i + 1/2,j - 1/2,k}^{\theta } + \Delta y\overline{E}_{y; \, i + 1/2,j,k - 1/2}^{\theta } - \Delta y\overline{E}_{y; \, i + 1/2,j,k + 1/2}^{\theta } } \right);$$

38 $$\partial_{t} \overline{B}_{y; \, i,j - 1/2,k}^{\theta } { = } - \frac{1}{\Delta x\Delta z}\left( {\Delta x\overline{E}_{x; \, i,j - 1/2,k + 1/2}^{\theta } - \Delta x\overline{E}_{x; \, i,j - 1/2,k - 1/2}^{\theta } + \Delta z\overline{E}_{z; \, i - 1/2,j - 1/2,k}^{\theta } - \Delta z\overline{E}_{z; \, i + 1/2,j - 1/2,k}^{\theta } } \right);$$

39 $$\partial_{t} \overline{B}_{z; \, i,j,k + 1/2}^{\theta } { = } - \frac{1}{\Delta x\Delta y}\left( {\Delta x\overline{E}_{x; \, i,j - 1/2,k + 1/2}^{\theta } - \Delta x\overline{E}_{x; \, i,j + 1/2,k + 1/2}^{\theta } + \Delta y\overline{E}_{y; \, i + 1/2,j,k + 1/2}^{\theta } - \Delta y\overline{E}_{y; \, i - 1/2,j,k + 1/2}^{\theta } } \right).$$

Figure 7 shows us schematically how our update strategy allows us to access any accuracy of update for the electric fields, going from the highest order accuracy to the lowest order accuracy. In this figure and the text that follows, the high-order components are superscripted with “HO”; low-order components are superscripted with “LO”. Using these, the forward Euler approximants can be constructed for any SSP-RK time-stepping strategy. If a zone in those forward Euler approximants is not within the PCP domain, its local $\theta_{i,j,k}$ will be lowered even further in each successive iteration till it becomes PCP. Realize that this procedure is fully explicit, i.e., it does not require any fluxes or electric fields that are obtained through an implicit process. Realize too that this process is guaranteed to ensure that all zones are brought into the PCP domain.

## Accuracy Analysis for Divergence Involution Constrained Hyperbolic PDE Systems

We focus on three PDE systems which have an involution constraint involving one or more vector fields. The first PDE system is drawn from CED which has two involution constraints associated with two vector fields. The vector fields are the electric flux density and the magnetic flux density. These two flux densities evolve in response to the curl of the magnetic field intensity and the electric field intensity, respectively. CED is based on Maxwell’s equations which are fundamental equations of nature and cannot be derived from any other equation. The constitutive relations that relate the field intensities to the flux densities are related to the permittivity and permeability of the material being considered. The second PDE system is drawn from classical MHD and the third PDE system is drawn from RMHD. The latter two PDE systems have an involution constraint involving the divergence of the magnetic field. The magnetic field evolves in response to the curl of an electric field for the latter two PDE systems. The MHD and RMHD equations can be derived from a combination of the equations of fluid dynamics and Maxwell’s equations. The electric field can, therefore, be written constitutively in terms of a cross product between the velocity field and the magnetic field.

For the time-update, we used a third-order SSP-RK scheme from Shu and Osher [77] and a five-stage fourth-order SSP-RK scheme from Spiteri and Ruuth [80]. For the fifth-, seventh-, and ninth-order schemes, we had to reduce the timestep as the mesh was refined. The base-level grid for all of the accuracy tests was run with a CFL of 0.3. For the spatially third-order accurate scheme, we used a third-order SSP-RK scheme, and for the fifth-, seventh-, and ninth-order accurate schemes, we used the fourth-order SSP-RK time stepping. Consequently, for the fifth-, seventh-, and ninth-order schemes, we reduced the time-step size as the mesh was refined, so that the temporal error remained dominated by the spatial error. When a spatially fifth-order scheme is used with a temporally fourth-order accurate time-stepping strategy, every doubling of the mesh requires a reduction in the timestep that goes as Δ*t* → Δ*t* (1/2)^5/4^. Similarly, when a spatially seventh-order scheme is used with a temporally fourth-order time-stepping strategy, every doubling of the mesh requires a reduction in the timestep that goes as Δ*t* →Δ*t* (1/2)^7/4^. The same strategy can be generalized to the ninth order.

### Accuracy Analysis for Computational Electrodynamics (CED)

In this sub-section, we perform an accuracy analysis for the CED system. The equations of CED can be written as two evolutionary curl-type equations for the electric displacement (extended Ampere’s law) and the magnetic induction (Faraday’s law)

40 $$\frac{{\partial {\mathbf{D}}}}{\partial t} - \nabla \times {\mathbf{H}} = - {\mathbf{J}}{ ; }\quad \, \frac{{\partial {\mathbf{B}}}}{\partial t} + \nabla \times {\mathbf{E}} = 0.$$

The electric displacement and the magnetic induction vector fields also satisfy the following two non-evolutionary constraint equations given by

41 $$\nabla \cdot {\mathbf{D}} = \rho_{E} { ; }\quad \, \nabla \cdot {\mathbf{B}} = 0.$$

Here, $\rho_{E}$ is the electric charge density and “**J**” is the current density. In material media, the electric displacement vector is also related to the electric field vector, and the magnetic induction vector is related to the magnetic field vector. The constitutive relations are given by

42 $${\mathbf{D}} = \varepsilon {\mathbf{E}};\quad \, {\mathbf{B}} = {\mathbf{\mu H}},$$

where, in general, ε is a symmetric 3 × 3 permittivity tensor and ${{\boldsymbol{\upmu}}}$ is a symmetric 3 × 3 magnetic permeability tensor that depends on material properties.

We perform an accuracy study for the above system using a 3D plane-polarized electromagnetic wave that has been formulated in Balsara et al. [37]. This test problem consists of a plane-polarized electromagnetic wave propagating in a vacuum along the diagonal of a 3D Cartesian mesh spanning $\left[ { - 0.5,0.5} \right]^{3}$. Periodic boundary conditions are enforced. The detailed setup is given in Balsara et al. [17], and therefore, we do not provide the details here. Table 1 provides the accuracy analysis for the $B_{y}$ variable. We see that all the presented schemes reach their design accuracies efficiently.

**Table 1:** Accuracy of the 3D electromagnetic wave problem for the CED system using the divergence-preserving AFD-WENO schemes presented here; the $B_{y}$ variable is shown

Order 2	*L*_1_ error	*L*_1_ accuracy	*L*_inf_ error	*L*_inf_ accuracy
16^3^	7.376 22E−02		1.354 89E−01	
32^3^	2.024 38E−02	1.87	4.217 36E−02	1.68
64^3^	6.182 36E−03	1.71	1.445 76E−02	1.54
128^3^	1.681 61E−03	1.88	5.871 10E−03	1.30
Order 3				
16^3^	2.278 28E−02		3.545 11E−02	
32^3^	3.023 72E−03	2.91	4.727 80E−03	2.91
64^3^	3.815 55E−04	2.99	5.993 18E−04	2.98
128^3^	4.780 18E−05	3.00	7.507 24E−05	3.00
Order 5				
16^3^	9.703 35E−04		1.536 15E−03	
32^3^	3.175 19E−05	4.93	4.964 51E−05	4.95
64^3^	1.000 29E−06	4.99	1.572 39E−06	4.98
128^3^	3.136 01E−08	5.00	4.926 23E−08	5.00
Order 7				
16^3^	4.055 52E−05		6.423 64E−05	
32^3^	2.719 03E−07	7.22	4.264 64E−07	7.23
48^3^	1.603 55E−08	6.98	2.515 01E−08	6.98
64^3^	2.144 20E−09	6.99	3.370 40E−09	6.99
Order 9				
16^3^	1.882 89E−05		2.943 54E−05	
32^3^	3.375 18E−09	12.45	5.278 92E−09	12.45
48^3^	6.332 00E−11	9.81	9.944 03E−11	9.80
64^3^	3.483 35E−12	10.08	5.506 82E−12	10.06

### Accuracy Analysis for Magnetohydrodynamics (MHD)

In this sub-section, we consider the 2D MHD system to study the accuracy of the presented schemes. The MHD system for an ideal fluid can be written in conservation form as

43 $$\frac{\partial }{\partial t}\left( \begin{gathered} \rho \\ \rho v_{x} \\ \rho v_{y} \\ \rho v_{z} \\ \varepsilon \\ B_{x} \\ B_{y} \\ B_{z} \\ \end{gathered} \right) + \frac{\partial }{\partial x}\left( \begin{gathered} \rho v_{x} \\ \rho v_{x}^{2} + p + {\mathbf{B}}^{2} /8\uppi - B_{x}^{2} /4\uppi \\ \rho v_{x} v_{y} - B_{x} B_{y} /4\uppi \\ \rho v_{x} v_{z} - B_{x} B_{z} /4\uppi \\ \left( {\varepsilon + p + {\mathbf{B}}^{2} /8\uppi } \right)v_{x} - B_{x} \left( {{\mathbf{v}} \cdot {\mathbf{B}}} \right)/4\uppi \\ 0 \\ \left( {v_{x} B_{y} - v_{y} B_{x} } \right) \\ - \left( {v_{z} B_{x} - v_{x} B_{z} } \right) \\ \end{gathered} \right) + \frac{\partial }{\partial y}\left( \begin{gathered} \rho v_{y} \\ \rho v_{y} v_{x} - B_{y} B_{x} /4\uppi \\ \rho v_{y}^{2} + p + {\mathbf{B}}^{2} /8\uppi - B_{y}^{2} /4\uppi \\ \rho v_{y} v_{z} - B_{y} B_{z} /4\uppi \\ \left( {\varepsilon + p + {\mathbf{B}}^{2} /8\uppi } \right)v_{y} - B_{y} \left( {{\mathbf{v}} \cdot {\mathbf{B}}} \right)/4\uppi \\ - \left( {v_{x} B_{y} - v_{y} B_{x} } \right) \\ 0 \\ \left( {v_{y} B_{z} - v_{z} B_{y} } \right) \\ \end{gathered} \right) = 0,$$

where $\varepsilon = \rho {\mathbf{v}}^{2} /2 + p/(\gamma - 1) + {\mathbf{B}}^{2} /8\uppi$ is the total energy density. The density is denoted by ρ; the pressure is written as “*p*”; the velocity components are given by $v_{x}$, $v_{y}$, $v_{z}$; the magnetic field components by $B_{x}$, $B_{y}$, $B_{z}$. “γ” denotes the ratio of specific heats. The usual challenge in MHD simulations has been that when the velocities or magnetic fields become too large, the pressure can become zero or negative.

In Balsara [10], we presented a genuinely 2D vortex problem for the above MHD system. The problem consists of a smoothly varying and dynamically stable vortex that moves diagonally. In Balsara [10], the explicit expressions for the setup have been provided; therefore, we do not describe the setup details here. In Table 2 , we show the accuracy analysis for the $B_{y}$ variable. To minimize the effect of small jumps in the velocity field at the periodic boundaries, we double the computational domain and stopping time for the fifth-, seventh-, and ninth-order schemes. We observe that the presented schemes are able to reach their design accuracy.

**Table 2:** Accuracy of the 2D MHD vortex problem for the divergence-preserving AFD-WENO schemes presented here; the $B_{y}$ variable is shown

Order 2	*L*_1_ error	*L*_1_ accuracy	*L*_inf_ error	*L*_inf_ accuracy
32^2^	1.382 48E−02		2.147 86E−01	
64^2^	3.828 61E−03	1.85	6.569 15E−02	1.71
128^2^	9.499 95E−04	2.01	1.742 73E−02	1.91
256^2^	2.611 12E−04	1.86	4.821 06E−03	1.85
Order 3				
32^2^	5.396 16E−03		7.647 76E−02	
64^2^	8.636 01E−04	2.64	1.149 15E−02	2.73
128^2^	1.119 66E−04	2.95	1.425 16E−03	3.01
256^2^	1.411 20E−05	2.99	1.772 98E−04	3.01
Order 5				
32^2^	3.696 66E−03		2.160 03E−01	
64^2^	4.528 50E−04	3.03	2.659 82E−02	3.02
128^2^	1.789 24E−05	4.66	1.126 14E−03	4.56
256^2^	5.762 84E−07	4.96	3.616 06E−05	4.96
Order 7				
32^2^	1.978 98E−03		1.092 11E−01	
64^2^	7.348 85E−05	4.75	4.461 31E−03	4.61
128^2^	7.198 14E−07	6.67	4.648 95E−05	6.58
256^2^	5.892 00E−09	6.93	3.761 16E−07	6.95
Order 9				
32^2^	2.125 89E−03		1.296 15E−01	
64^2^	1.383 12E−05	7.26	8.223 83E−04	7.30
128^2^	3.666 05E−08	8.56	2.449 37E−06	8.39
256^2^	7.700 28E−11	8.90	5.069 64E−09	8.92

### Accuracy Analysis for Relativistic Magnetohydrodynamics (RMHD)

In this sub-section, we consider the 2D RMHD system to study the accuracy of the presented schemes. The RMHD system in two dimensions can be written in conservation form as

44 $$\frac{\partial }{\partial t}\left( \begin{gathered} \rho W \\ m_{x} \\ m_{y} \\ m_{z} \\ \varepsilon \\ B_{x} \\ B_{y} \\ B_{z} \\ \end{gathered} \right) + \frac{\partial }{\partial x}\left( \begin{gathered} \rho Wv_{x} \\ m_{x} v_{x} + p + \frac{{|b|^{2} }}{2} - B_{x} b_{x} /W \\ m_{y} v_{x} - B_{x} b_{y} /W \\ m_{z} v_{x} - B_{x} b_{z} /W \\ m_{x} \\ 0 \\ \left( {v_{x} B_{y} - v_{y} B_{x} } \right) \\ - \left( {v_{z} B_{x} - v_{x} B_{z} } \right) \\ \end{gathered} \right) + \frac{\partial }{\partial y}\left( \begin{gathered} \rho Wv_{y} \\ m_{x} v_{y} - B_{y} b_{x} /W \\ m_{y} v_{y} + p + \frac{{|b|^{2} }}{2} - B_{y} b_{y} /W \\ m_{z} v_{y} - B_{y} b_{z} /W \\ m_{y} \\ - \left( {v_{x} B_{y} - v_{y} B_{x} } \right) \\ 0 \\ \left( {v_{y} B_{z} - v_{z} B_{y} } \right) \\ \end{gathered} \right) = 0.$$

Here, ρ is the density, W is the Lorentz factor, the set $\left\{ {\varepsilon ,m_{x} ,m_{y} ,m_{z} } \right\}$ forms the four-momentum density, ${\mathbf{v}} = \left( {v_{x} ,v_{y} ,v_{z} } \right)^{{\mathrm{T}}}$ is the velocity vector, p is the pressure, and $B_{x} ,B_{y} ,B_{z}$ are the components of the magnetic field. The speed of light is assumed to be unity; therefore, the Lorentz factor is defined by $W = 1/\sqrt {1 - {\mathbf{v}}^{2} }$. The conserved quantities $m_{i}$ and ε are given by

45 $$m_{i} = \left( {\rho hW^{2} + {\mathbf{B}}^{2} } \right)v_{i} - ({\mathbf{v}} \cdot {\mathbf{B}})B_{i}; \; \; \varepsilon = \rho hW^{2} - p + \frac{{{\mathbf{B}}^{2} }}{2} + \frac{{{\mathbf{v}}^{2} {\mathbf{B}}^{2} - ({\mathbf{v}} \cdot {\mathbf{B}})^{2} }}{2},$$

where $h = 1 + \frac{\gamma }{\gamma - 1}\frac{p}{\rho }$ is the specific enthalpy and γ denotes the polytropic index. In the above, Latin indices range from 1 to 3. The components and magnitude of the covariant magnetic field are defined as

46 $$b_{\mu } = W\left( {{\mathbf{v}} \cdot {\mathbf{B}},\frac{{B_{i} }}{{W^{2} }} + \left( {{\mathbf{v}} \cdot {\mathbf{B}}} \right)v_{i} } \right); \; \; |b|^{2} = \frac{{{\mathbf{B}}^{2} }}{{W^{2} }} + \left( {{\mathbf{v}} \cdot {\mathbf{B}}} \right)^{2} ,$$

where the Greek indices range from 0 to 3. Cai et al. [43] have presented a provably convergent and robust Newton-Raphson method to extract the primitives from the conservative variables. We use the same strategy here. We define the vector of conservative variables as ${\mathbf{U}} = \left( {\rho W,m_{x} ,m_{y} ,m_{z} ,\varepsilon ,B_{x} ,B_{y} ,B_{z} } \right)^{{\mathrm{T}}}$. Balsara and Kim [28] suggested that a better way to reconstruct the state variables (given in vector ${\mathbf{U}}$) is to reconstruct in the special vector of primitive variables given by ${\mathbf{V}} = \left( {\rho ,v_{x} W,v_{y} W,v_{z} W,p,B_{x} ,B_{y} ,B_{z} } \right)^{{\mathcal{\mathrm{T}}}}$. We adopt the same idea and reconstruct ${\mathbf{V}}$ to obtain the reconstructed primitives and then use the change of variable matrix $\frac{{\partial {\mathbf{U}}}}{{\partial {\mathbf{V}}}}$ to obtain the derivative terms of the form $\frac{{\partial {\mathbf{U}}}}{\partial x}$ using the formula $\frac{{\partial {\mathbf{U}}}}{\partial x} \cong \frac{{\partial {\mathbf{U}}}}{{\partial {\mathbf{V}}}}\frac{{\partial {\mathbf{V}}}}{\partial x}$.

Balsara and Kim [28] have presented a genuinely 2D vortex problem for the RMHD system. The problem consists of a smoothly varying and dynamically stable vortex that moves diagonally in the square computational domain. The detailed derivation and setup expressions are given in Balsara and Kim [28]; therefore, we do not describe the setup details here. In Table 3, we show the accuracy analysis for the $B_{y}$ variable. To minimize the effect of small jumps in the velocity field at the periodic boundaries, we double the computational domain and stopping time for the seventh- and ninth-order schemes. From Table 3, we see that the presented schemes are able to reach their design accuracy for the RMHD system.

**Table 3:** Accuracy of the 2D RMHD vortex problem for the divergence-preserving AFD-WENO schemes presented here; the $B_{y}$ variable is shown

Order 3	*L*_1_ error	*L*_1_ accuracy	*L*_inf_ error	*L*_inf_ accuracy
64^2^	4.042 60E−03		1.283 01E−01	
128^2^	8.499 00E−04	2.25	3.814 57E−02	1.75
256^2^	1.238 60E−04	2.78	7.303 76E−03	2.38
512^2^	1.631 54E−05	2.92	1.047 66E−03	2.80
Order 5				
64^2^	7.014 18E−04		3.059 50E−02	
128^2^	5.302 57E−05	3.73	3.340 89E−03	3.19
256^2^	2.116 97E−06	4.65	1.886 30E−04	4.15
512^2^	9.579 48E−08	4.47	6.465 21E−06	4.87
Order 7				
64^2^	1.796 95E−03		2.303 25E−01	
128^2^	2.575 14E−04	2.80	3.108 41E−02	2.89
256^2^	3.684 27E−06	6.13	9.957 26E−04	4.96
512^2^	4.752 28E−08	6.28	1.659 06E−05	5.91
Order 9				
64^2^	2.821 70E−03		2.262 98E−01	
128^2^	3.105 40E−04	3.18	3.409 35E−02	2.73
256^2^	1.559 05E−06	7.64	3.144 28E−04	6.76
512^2^	3.714 25E−09	8.71	1.527 84E−06	7.69

## Test Problems for Hyperbolic PDE Systems with a Divergence-Based Involution Constraint

We present several 2D test problems for the CED, MHD, and RMHD systems. For each of the problems, we take a CFL number of 0.4 unless stated otherwise.

### Test Problems Involving CED

**Fig. 10:**
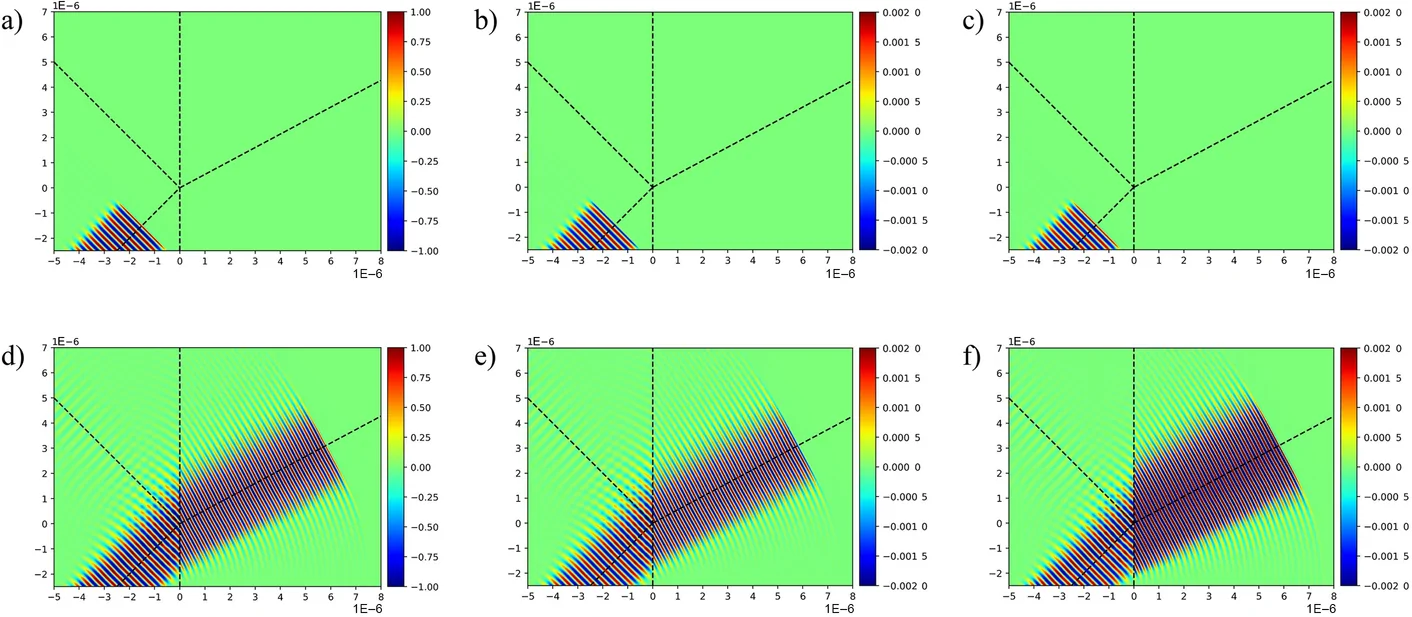
CED: refraction of a compact electromagnetic beam by a dielectric slab. **a–c** *B*_*z*_, *D*_*x*_, and *D*_*y*_ at the initial time; **d–f** show the same at a final time of 4 × 10^−14^ s. The surface of the dielectric slab is identified by the dashed vertical black line. The oblique dashed black lines demarcate the angle of incidence, the angle of refraction, and the angle of reflection

In this sub-section, we present a set of two test problems for the CED system. The first problem shows the refraction of a compact electromagnetic beam by a dielectric slab. The problem was described in detail in Balsara et al. [37]; therefore, we do not repeat the description here. The problem is set up on a rectangular xy-domain that spans $[ - 5,8] \times [ - 2.5,7]\; \upmu \mathrm{m}$. For the simulation shown, we use a fifth-order accurate scheme using a 1 300 × 900 zone mesh. The permittivity increases in a tapered fashion from $\varepsilon_{0}$ for x<0 to $2.25 \, \varepsilon_{0}$ for x>0. A compact electromagnetic beam is incident on the slab at an angle of incidence given by $45^\circ$. Figures 10a–c show, respectively, $B_{z}$, $D_{x}$, and $D_{y}$ at the initial time t=0. Figures 10d–f show the same profiles at the final time of $4.0 \times 10^{ - 14}$ s. The surface of the dielectric slab is shown by a vertical dashed black line. The inclined dashed black lines show the angles of incidence, refraction, and reflection, and these black lines are over-plotted on the field components to guide the eye. According to Snell’s law, the angle of refraction should be $28.12^\circ$, since the refractive index of the dielectric slab is 1.5. We see that the code obtains the correct angle of refraction. We also observe that some of the radiation is reflected from the surface of the slab. The presence of a reflected wave is consistent with the Fresnel conditions for transmission and reflection of radiation at dielectric interfaces.

**Fig. 11:**
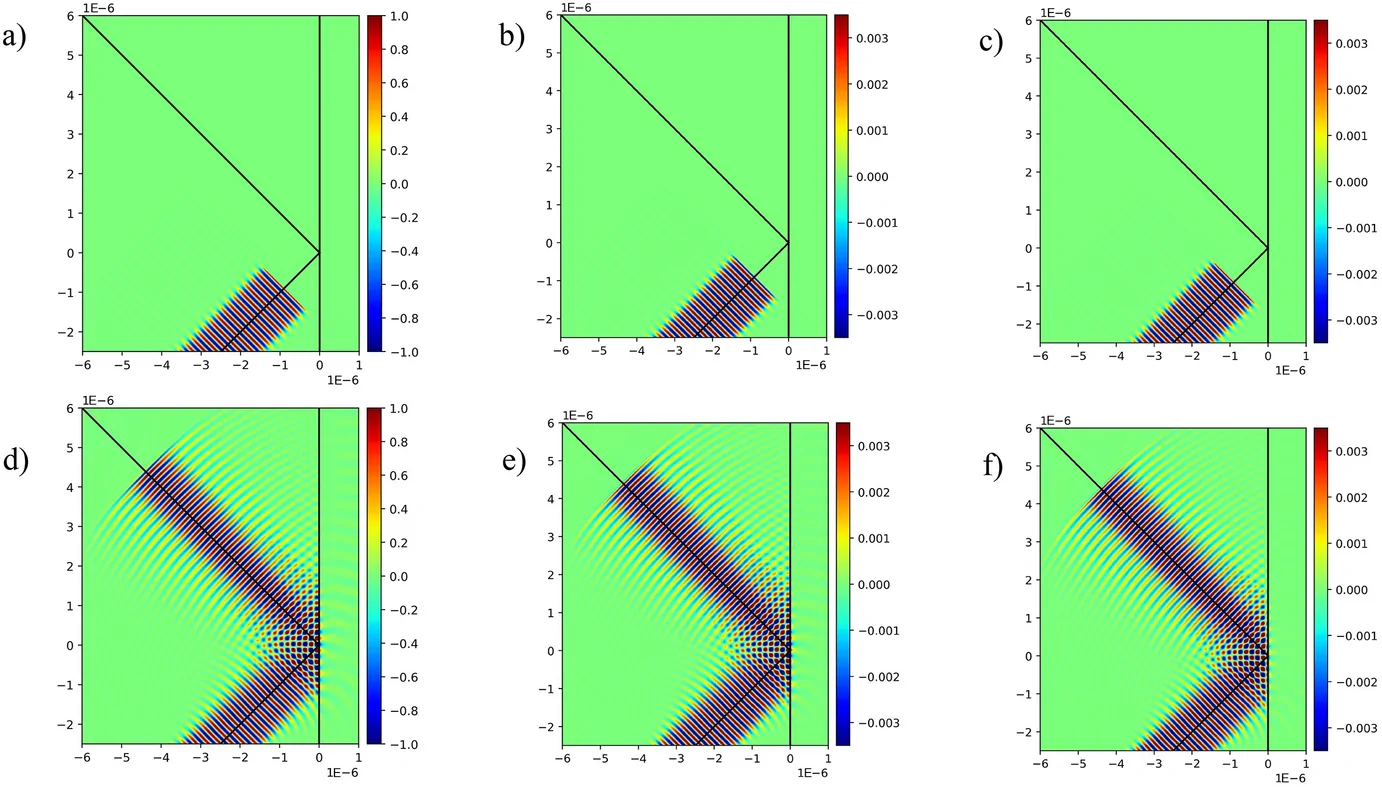
CED: total internal reflection of a compact electromagnetic beam by a dielectric slab. **a–c** *B*_*z*_, *D*_*x*_, and *D*_*y*_ at the initial time; **d–f** show the same at a final time of 5 × 10^−14^ s. The surface of the dielectric slab is identified by the vertical black line. The oblique black lines demarcate the angle of incidence and the angle of total internal reflection

The second problem shows the total internal reflection of a compact electromagnetic beam by a dielectric slab. This problem was also described in detail in Balsara et al. [37]; therefore, we do not repeat the description here. This problem is set up on a rectangular xy-domain that spans $[ - 6,1] \times [ - 2.5,6]\; \upmu \mathrm{m}$. We use a seventh-order accurate scheme on a 700 × 850 zone mesh. Here, ε is chosen such that it has a value of $4.0 \, \varepsilon_{0}$ for x⩽0 and tapers rapidly to the ambient value of $\varepsilon_{0}$ for x>0. The value of permittivity for x<0 implies a refractive index of 2 for the dielectric slab. For this mesh, the taper width that is applied to the variation in the permittivity is 0.25 times a zone width. The wavelength in the dielectric medium corresponds to about 30 zones. For such a slab, the critical angle for total internal reflection is $30^{\circ}$. The angle of the incident radiation is $45^{\circ}$, with the result that the incident radiation will undergo total internal reflection. Figures 11a–c show, respectively, $B_{z}$, $D_{x}$, and $D_{y}$ at the time t=0. The same profiles at a final time of $t = 5 \times 10^{ - 14}$ are shown in Figs. 11d–f. The surface of the dielectric slab is shown by a vertical black line. The inclined black lines for the incident and reflected rays are over-plotted on the field components to guide our eye. We see from the figures that the radiation undergoes total internal reflection, as expected. The results are consistent with those reported in Balsara and Kim [28], showing the occurrence of total internal reflection.

### Test Problems Involving MHD

In this sub-section, we present various 2D test problems for the MHD system. We begin by examining the Orszag-Tang problem that was first introduced in Orszag and Tang [70]. The Orszag-Tang problem is widely used as a test model for the emergence of MHD turbulence. The problem is initialized on a square domain that spans [0,2] × [0,2]. The ghost cells are filled with the periodic boundaries. The adiabatic constant is set as γ=5/3. The density is initialized to $\gamma^{2}$ all over the domain. The pressure is uniformly set to γ. The velocity field is set up as follows:

$${\mathbf{v}} = - \sin (\uppi y)\hat{x} + \sin (\uppi x)\hat{y}.$$

The magnetic field is initialized in a divergence-free manner using the following vector potential:

$$A_{z} = - \frac{{\sqrt {4\uppi } }}{2\uppi }\left( {\cos (2\uppi x) + 2\cos (\uppi y)} \right).$$

**Fig. 12:**
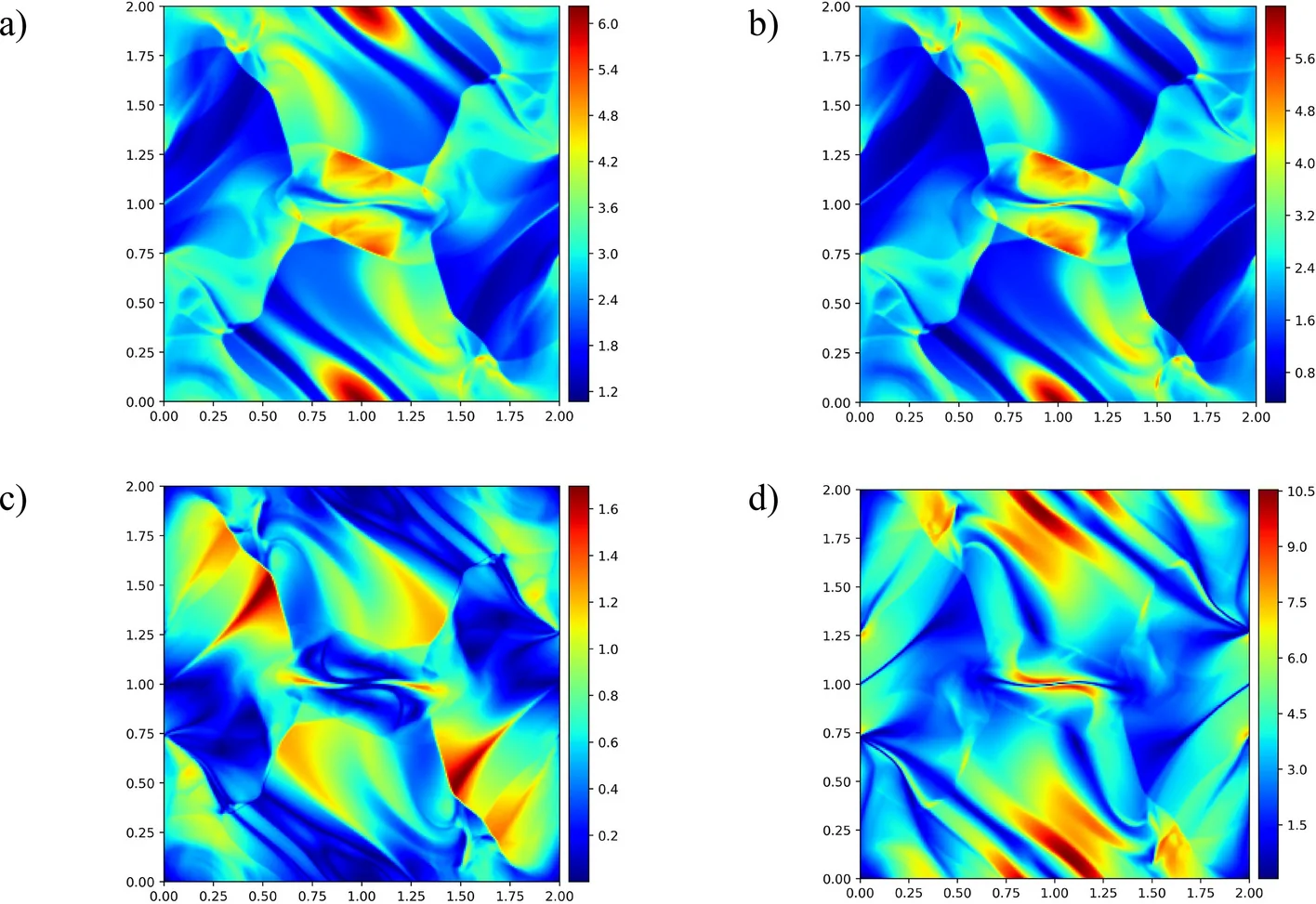
MHD: Orszag-Tang problem; **a** density, **b** pressure, **c** magnitude of the velocity, and **d** magnitude of the magnetic field vector using the ninth-order accurate scheme

We run simulations until a final time of *t* = 1 on a grid of 256 × 256 zones. We use the ninth-order scheme and show results for the density variable, pressure variable, magnitude of the velocity vector, and magnitude of the magnetic field vector in Fig. 12. We observe a close resemblance between our obtained results and those presented in Balsara [10]. The third-, fifth-, and seventh-order schemes also perform well on this problem; therefore, they are not shown here.

**Fig. 13:**
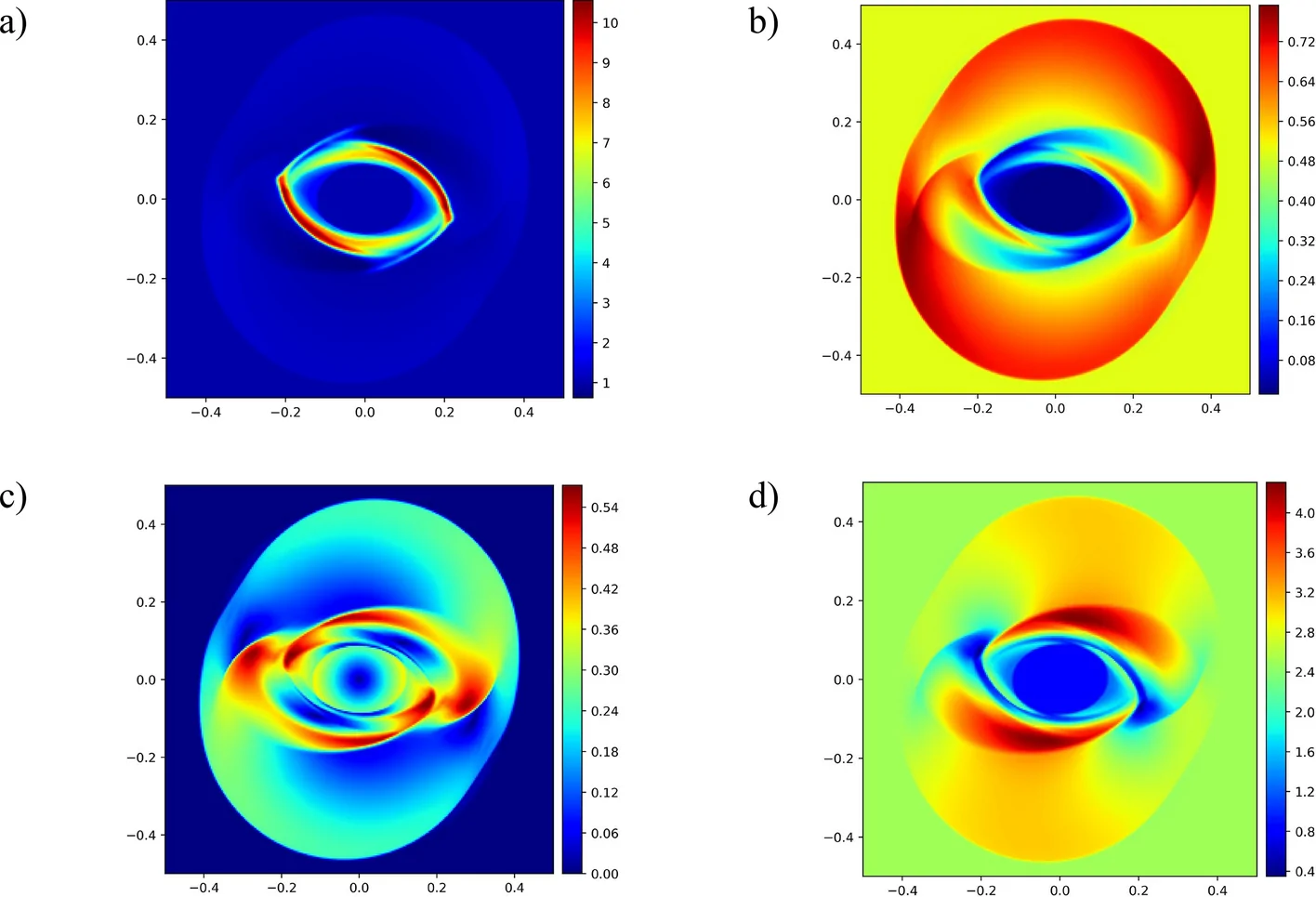
MHD: rotor problem; **a** density, **b** pressure, **c** magnitude of the velocity, and **d** magnitude of the magnetic field vector using the seventh-order accurate scheme

The second test problem is the 2D rotor problem that was first presented in Balsara and Spicer [36]; see also Balsara [10]. The computational domain spans the domain [−0.5, 0.5] × [−0.5, 0.5]. A dense and rapidly spinning cylinder is set up in the center of an initially stationary, light ambient fluid. The ambient fluid is initially at rest. A uniform magnetic field initially threads the two fluids. Its value is set to 2.5 units, and it initially points in the *x*-direction. The total pressure in the fluid is set to unity, i.e., *p* = 1. The density in the ambient fluid is uniformly set to unity, while the constant density in the rotor is 10 units out to a radius of 0.1. A linear taper is applied to the density between a radius of 0.1 and 0.13, so that the density in the rotor decreases linearly to the value of the density in the ambient fluid. Six zones are used for the taper to join the density of the two fluids. That number should be kept fixed if the resolution is increased or decreased. The initial angular velocity of the rotor is uniform out to a radius of 0.1. At this radius, the toroidal velocity has a value of one unit. The toroidal velocity decreases linearly from one unit to zero between a radius of 0.1 and 0.13, so that it joins the velocity of the ambient fluid at a radius of 0.13. The simulations were run until a final time of *t* = 0.29 using a mesh consisting of 256 × 256 zones. We use the seventh-order accurate scheme for this test problem. Figure 13 shows the results for the density variable, pressure variable, magnitude of the velocity vector, and magnitude of the magnetic field vector. We see that the results in Fig. 13 closely match the profiles presented in Balsara and Spicer [36] and Balsara [10]. The third-, fifth-, and ninth-order schemes also perform well on this problem; therefore, they are not shown here.

**Fig. 14:**
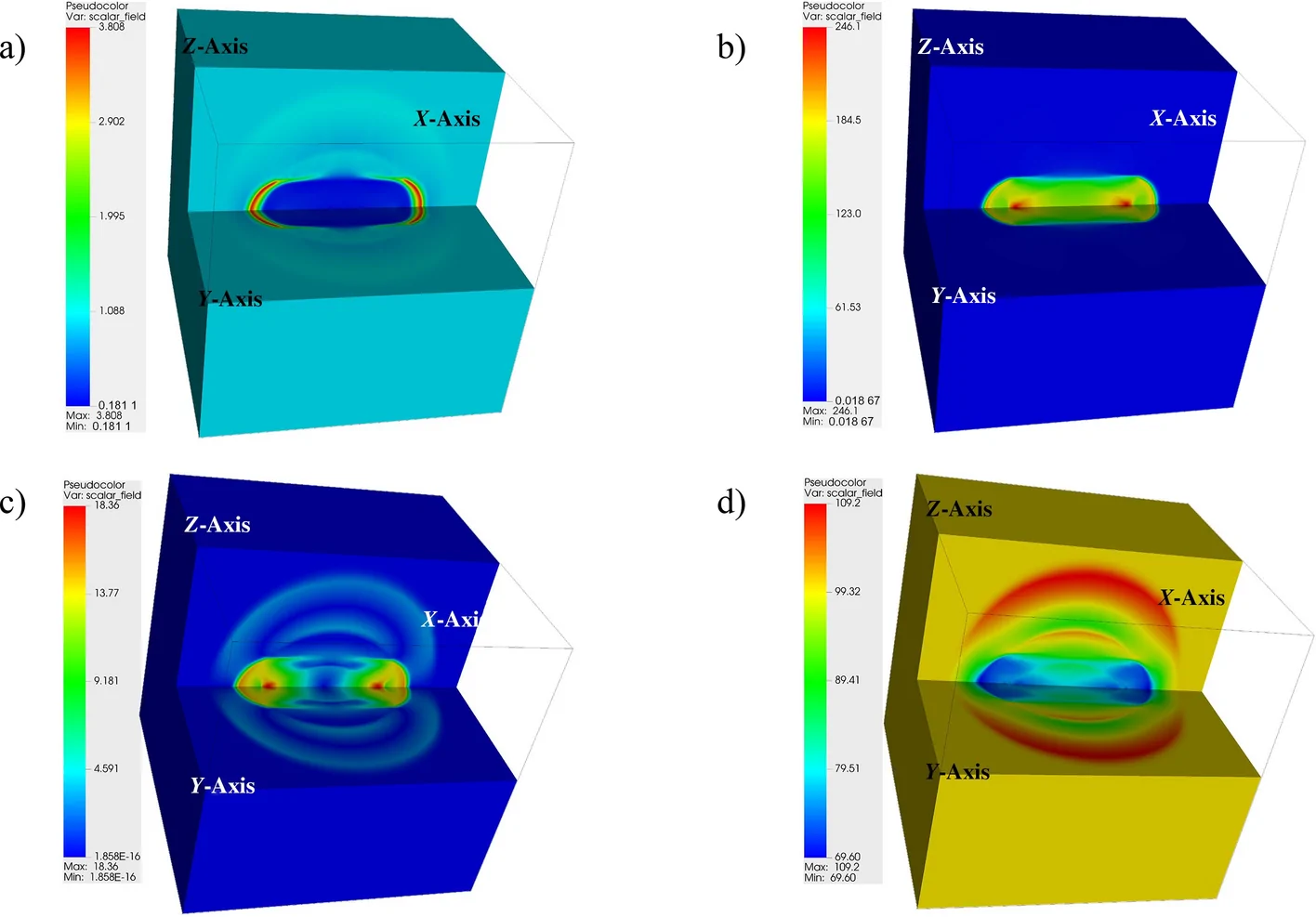
MHD: blast problem-I (BLAST-I); **a** density, **b** pressure, **c** magnitude of the velocity, and **d** magnitude of the magnetic field vector using the fifth-order accurate scheme. Here, we show the 3D variant of this problem

**Fig. 15:**
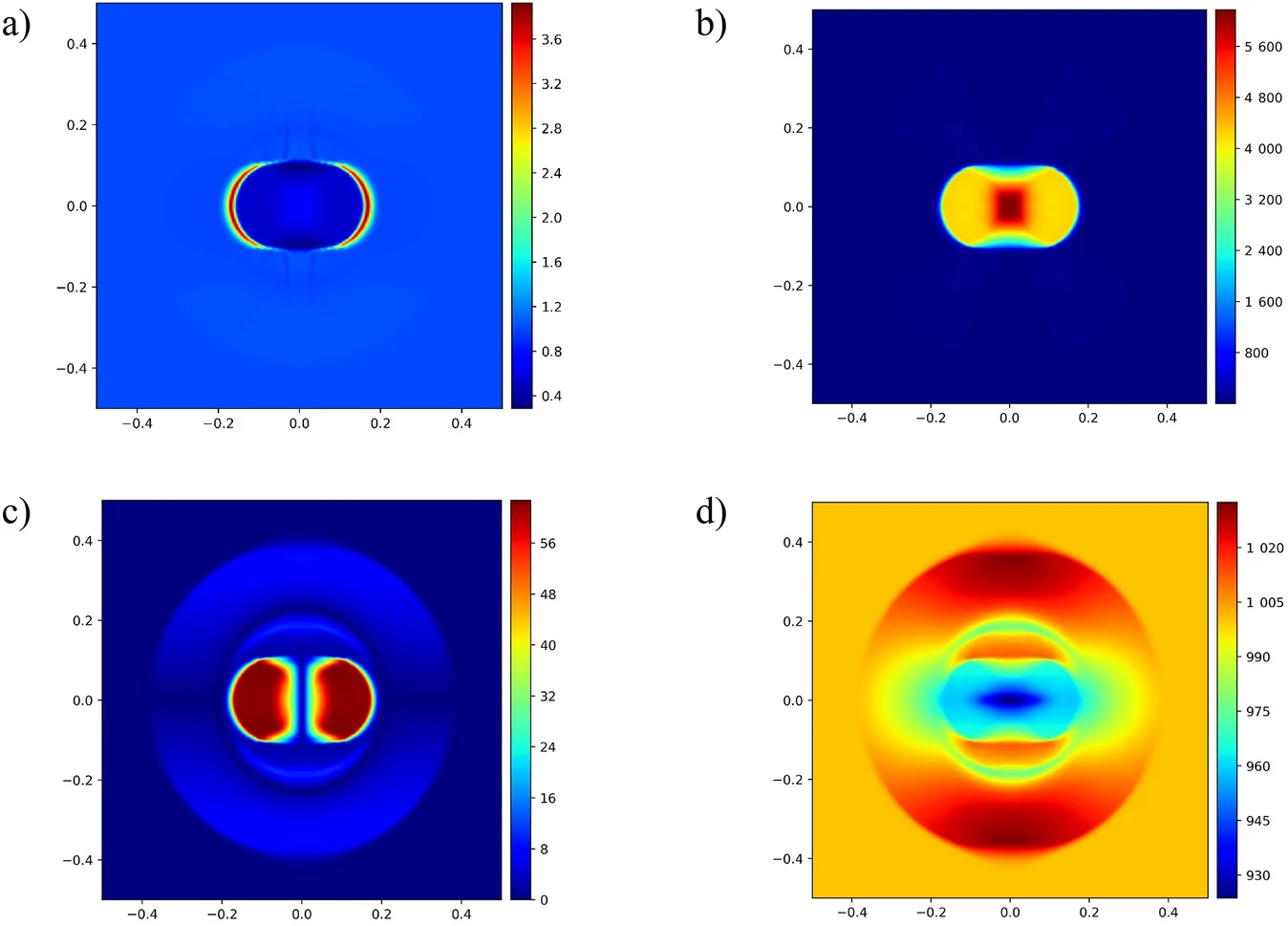
MHD: blast problem-II (BLAST-II); **a** density, **b** pressure, **c** magnitude of the velocity, and **d** magnitude of the magnetic field vector using the fifth-order accurate scheme

Next, we consider two variations of the blast problem (BLAST-I and BLAST-II). The first type is described in Balsara and Spicer [36], while the second case is sourced from Wu and Shu [84]. Both types are examples of stringent problems for the MHD system, because they correspond to very low values of plasma *β*. As a result, the problems require a PCP variant of the presented schemes (see Bhoriya et al. [39] and Sect. 5 for the detailed strategy). The first problem is run on a 3D grid with a domain $\left[ { - 0.5,0.5} \right] \times \left[ { - 0.5,0.5} \right] \times \left[ { - 0.5,0.5} \right]$ using a 300^3^ zone mesh. The second problem is configured on a unit square grid that consists of 400 × 400 zones and spans the domain [−0.5, 0.5] × [−0.5, 0.5]. Initially, the density for both types is uniformly set to unity and the velocity is set to zero. The two problems differ in terms of magnetic and pressure strengths. For the first problem type (BLAST-I), the pressure is uniformly set to 0.1 except within a central spherical region of radius 0.1, where it is elevated to 1 000. Additionally, a magnetic field with a magnitude of 100 is initialized along the *x*-direction, which makes the problem challenging for numerical schemes. While this problem was originally presented in two dimensions in Balsara and Spicer [36], here, we present the 3D variant of the same problem. We simulate the problem until a final time of *t* = 0.01 using the fifth-order scheme and show results for the density profile, pressure profile, magnitude of the velocity vector, and magnitude of the magnetic field vector in Fig. 14. There is a good similarity between the obtained results, and those presented in Balsara and Spicer [36]. For the second problem type (BLAST-II), the pressure within a central circle of radius 0.1 is elevated to 10 000, making the initial jump larger in the pressure variable. Additionally, a much stronger magnetic field with a magnitude of 1 000 is initialized along the *x*-direction which makes the problem extremely challenging for numerical schemes. Figure 15 displays the numerical results at time *t* = 0.001 obtained by the fifth-order scheme. The obtained results are consistent with those reported in Wu and Shu [84], highlighting the effectiveness of the PCP property of the presented schemes.

Next, we consider the astrophysical jet problem (Mach 800) from Balsara [14]. Following Wu and Shu [84], a magnetic field is added to this problem to simulate the MHD jet flows. The presence of a magnetic field makes this test more extreme. As a result, the problems require a PCP variant of the presented schemes (see Bhoriya et al. [39] and Sect. 5 for the detailed strategy). We consider three variations of the jet problem based on the strength of the magnetic field ($B_{y}$-component). All variants of the problem are configured on a 2D grid consisting of 400 × 600 zones and span the domain [−0.5, 0.5] × [0.0, 1.5]. Initially, the domain is filled with a static medium with uniform density of 0.1γ and uniform pressure of unity (p=1), where the adiabatic index γ is set to 1.4. The magnetic field is given by $\left( {B_{x} ,B_{y} ,B_{z} } \right) =$ $\left( {0,B_{a} ,0} \right)$. At the bottom boundary (y=0), a dense jet is injected through the inlet part |x|<0.05 with the states $\left( {\rho ,\;v_{x} ,\;v_{y} ,v_{z} ,p,B_{x} ,B_{y} ,B_{z} } \right) =$ $\left( {\gamma ,\;0,{\mkern 1mu} \;800,\;0,1,\;0,B_{a} ,0} \right)$. Outflow boundaries are employed at all the other boundaries. We consider the below three test cases (based on the value of $B_{a}$).

Jet-I Moderately magnetized case: $B_{a} = \sqrt {200}$ (corresponding plasma-beta, $\beta_{a} = 10^{ - 2}$).

Jet-II Strongly magnetized case: $B_{a} = \sqrt {2\;000}$ (corresponding $\beta_{a} = 10^{ - 3}$).

Jet-III Extremely strongly magnetized case: $B_{a} = \sqrt {20\;000}$ (corresponding $\beta_{a} = 10^{ - 4}$).

**Fig. 16:**
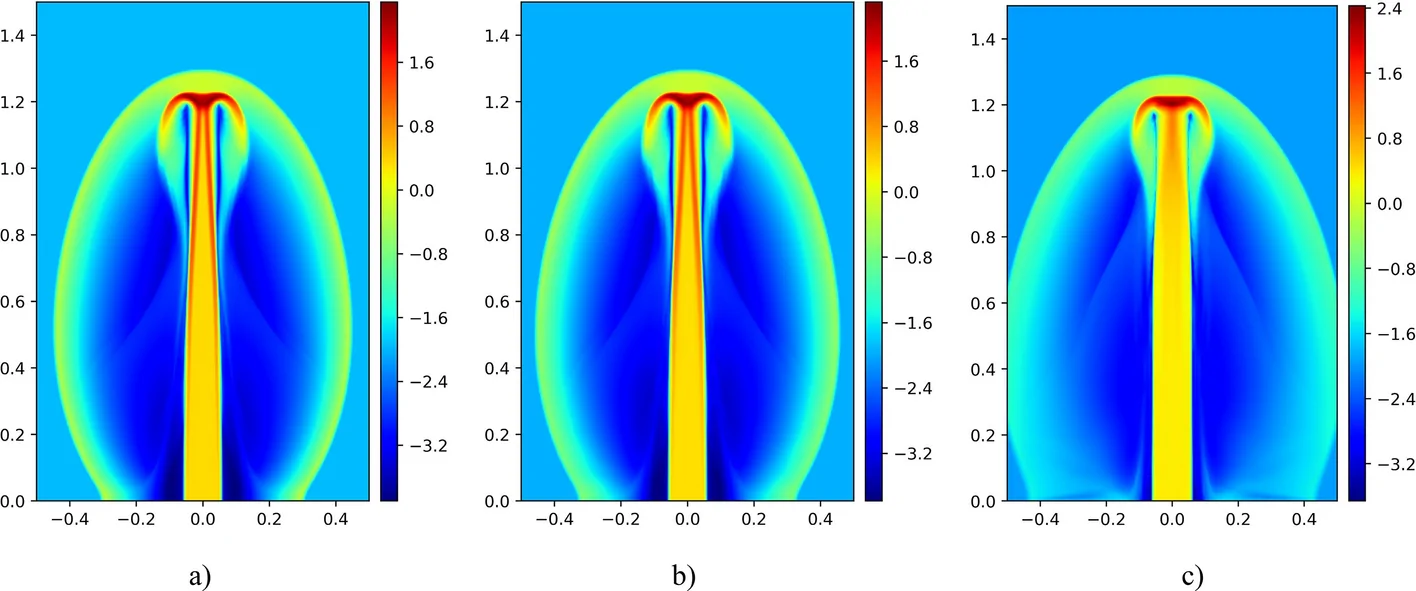
MHD: astrophysical jet problem (Jet-I, II, and III); **a–c** resulting density profiles on logarithmic scales for the Jet-I, Jet-II, and Jet-III problems, respectively. Seventh-order accurate schemes are used

**Fig. 17:**
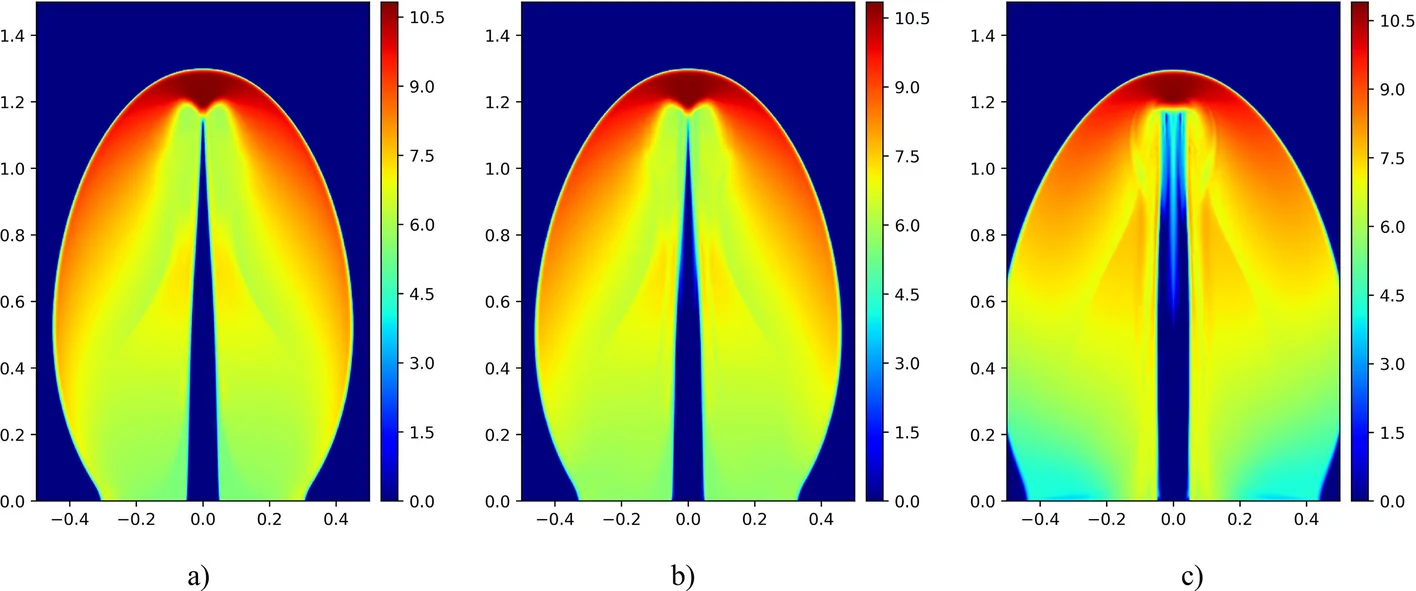
MHD: astrophysical jet problem (Jet-I, II, and III); **a–c** resulting pressure profiles on logarithmic scales for the Jet-I, Jet-II, and Jet-III problems, respectively. Seventh-order accurate schemes is used

All three variants were run to a stopping time of *t* = 0.002. Figures 16a–c show the resulting density profiles on logarithmic scales for the Jet-I, Jet-II, and Jet-III problems, respectively. Figures 17a–c show the resulting pressure profiles on logarithmic scales for the Jet-I, Jet-II, and Jet-III problems, respectively. A seventh-order accurate scheme is used for all the runs. From Figs. 16 and 17, we observe that the cocoon heads in the beam are well captured for these extreme cases, hence showing the robust performance of the proposed methods.

### Test Problems Involving RMHD

In this sub-section, we present various 2D test problems for the RMHD system. We begin by examining the blast problem for the RMHD system. The non-relativistic version of this test problem was first presented in Balsara and Spicer [36] and was extended to RMHD system by Komissarov [65]. Several variants of this test problem have been presented by Mignone and Bodo [69] using a slightly modified version of the moderately magnetized case in Komissarov [65]. In this sub-section, we use the setup given in Mignone and Bodo [69]; see also Balsara and Kim [28].

**Fig. 18:**
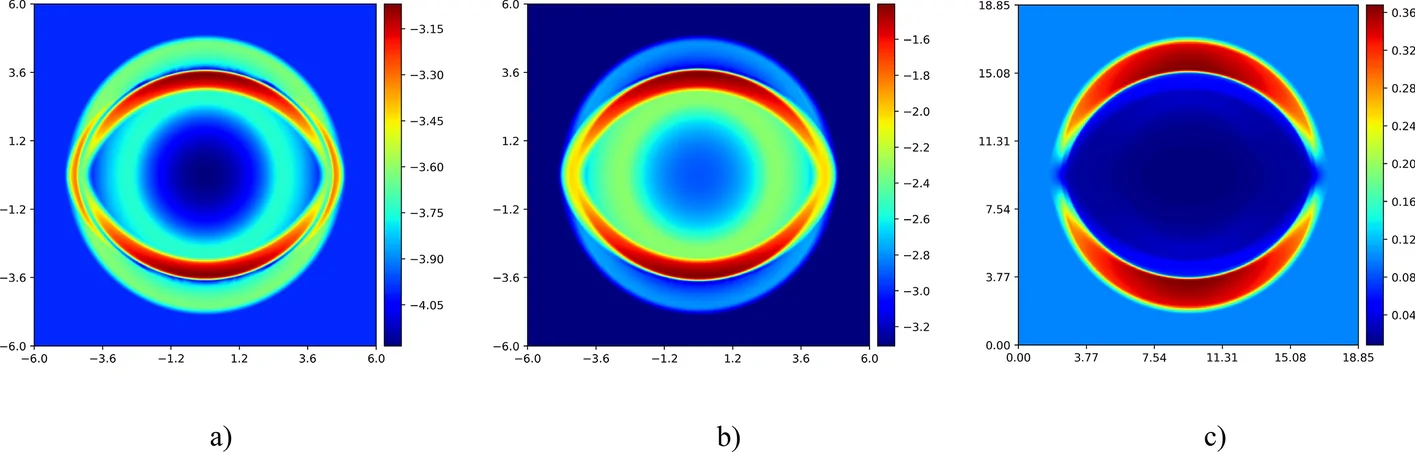
RMHD: blast problem; **a** logarithm of density, **b** logarithm of pressure, and **c** magnetic pressure using the fifth-order accurate scheme

The blast test problem is set up on a 2D square domain that spans [-6,6]×[-6,6]. Within a radius of 0.8, the explosion zone has a density of $10^{ - 2}$ and a pressure of 1. Outside a radius of 1 unit, the ambient medium has a density of $10^{ - 4}$ and a pressure of $5 \times 10^{ - 4}$. A linear taper is applied to the density and pressure from a radius of 0.8 to 1. Accordingly, both the density and pressure linearly decrease with increasing radius in that range of radii. The magnetic field is initialized in the *x*-direction and has a magnitude of 0.1. A polytropic index of γ=4/3 is used in this problem. The simulation is run to a final time of 4 using a fifth-order accurate scheme on a grid consisting of 512×512 zones. Obtained results are presented in Fig. 18. Figure 18a shows the logarithm of the density, Fig. 18b shows the logarithm of the pressure, and Fig. 18c shows the magnetic pressure at time *t* = 4. We see a close resemblance of the obtained results with the results presented in Mignone and Bodo [69] and Balsara and Kim [28].

Next, we consider the Orszag-Tang problem that was first introduced in Orszag and Tang [70] for the non-relativistic MHD and later was extended to RMHD in van der Holst et al. [59]; also see Wu and Shu [85]. Our setup is the same as in Wu and Shu [85]. The problem is initialized on a square domain that spans $[0,2\uppi ] \times [0,2\uppi ]$. The ghost cells are filled with the periodic boundaries. The adiabatic constant is set as γ=4/3. The density is initialized to 1 all over the domain. The pressure is uniformly set to 10. The velocity field is set up as follows:

$${\mathbf{v}} = - A\sin (y)\hat{x} + A\sin (x)\hat{y},$$

where $A=0.99/\sqrt{2}$. The magnetic field is initialized in a divergence-free manner using the following vector potential:

$$A_{z} = - \left( {\frac{\cos (2x)}{2} + \cos (y)} \right).$$

**Fig. 19:**
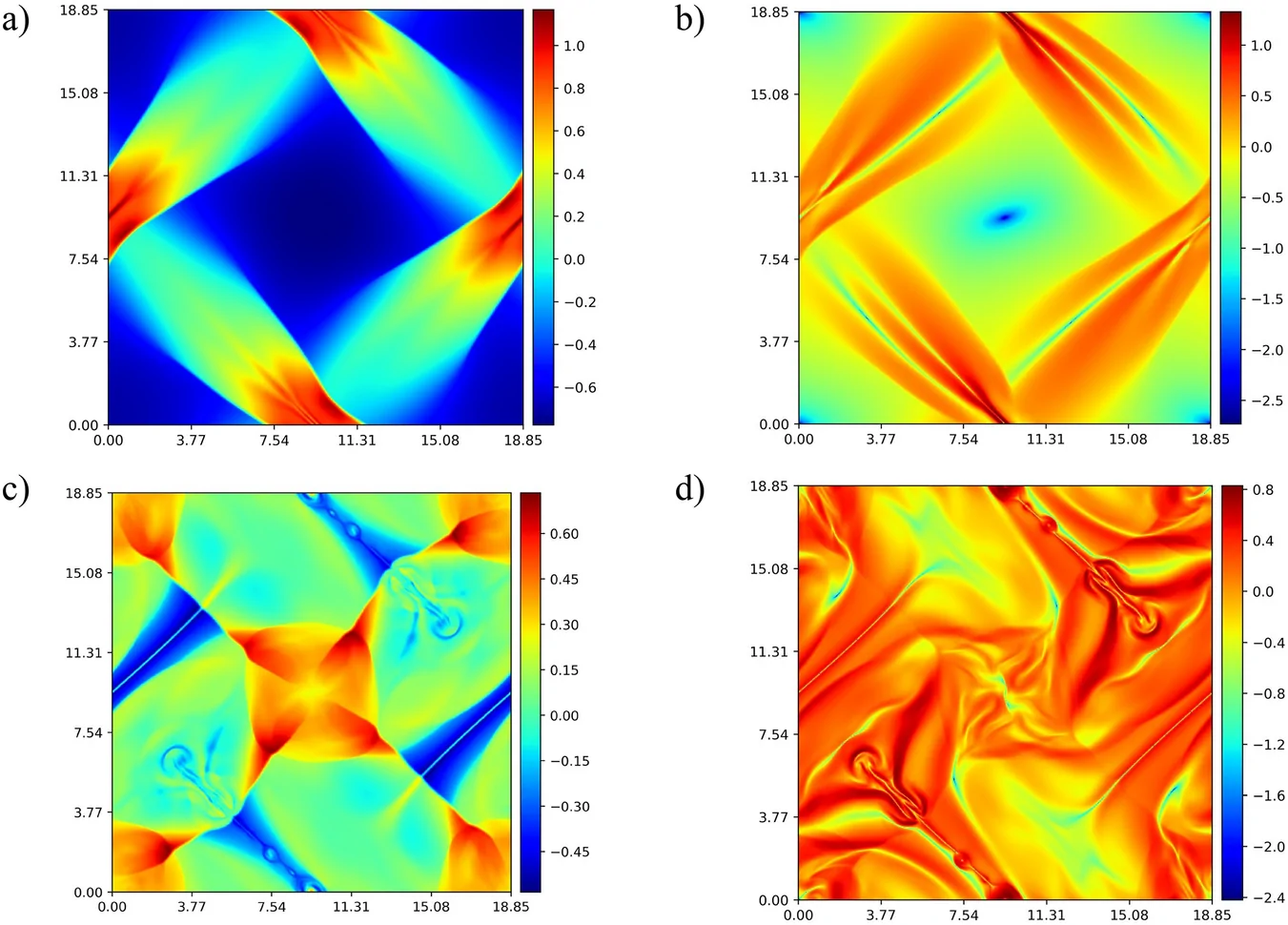
RMHD: Orszag-Tang problem; **a**, **b** logarithm of density and logarithm of magnetic pressure at time *t* = 2.818 127, and **c**, **d** logarithm of density and logarithm of magnetic pressure at time* t* = 6.855 8. The seventh-order accurate scheme is used

We run two simulations, one until a final time of *t* = 2.818 127 and another until a time of *t* = 6.855 8. We use a grid of 512 × 512 zones for both the runs. We use the seventh-order scheme and show the obtained results in Fig. 19. Figures 19a–b show the logarithm of the density and the logarithm of the magnetic pressure at time *t* = 2.818 127, and Figs. 19c–d show the logarithm of the density and the logarithm of the magnetic pressure at time *t* = 6.855 8. For both the runs, we observe a close resemblance between our obtained results and those presented in Wu and Shu [85]. The third-, fifth-, and ninth-order schemes also perform well on this problem; therefore, they are not shown here.

Next, we consider the Shock-cloud interaction problem from Wu and Shu [85]. The problem describes the dynamics of a high-density cloud when it is hit by a strong shock wave. The problem is initialized on a 2D domain that spans [-0.2,1.2]×[0,1]. We use the inflow boundary conditions at the left boundary and outflow boundary conditions at the remaining boundaries. The inflow boundary states are the same as the left shock states. A right moving shock at *x* = 0.05 is initialized using the following states:

$$\left( {\rho ,{\mathbf{v}},{\mathbf{B}},p} \right) = \left\{ \begin{gathered} \left( {3.868\;59,0.68, \, 0, \, 0, \, 0, \, 0.849\;81, \, - \, 0.849\;81, \, 1.251\;15} \right)\;\;\;{\text{if }}x < 0.05, \hfill \\ \left( {1, \, 0, \, 0, \, 0, \, 0, \, 0.161\;06, \, 0.161\;06, \, 0.05} \right){\text{ otherwise}}. \hfill \\ \end{gathered} \right.$$

**Fig. 20:**
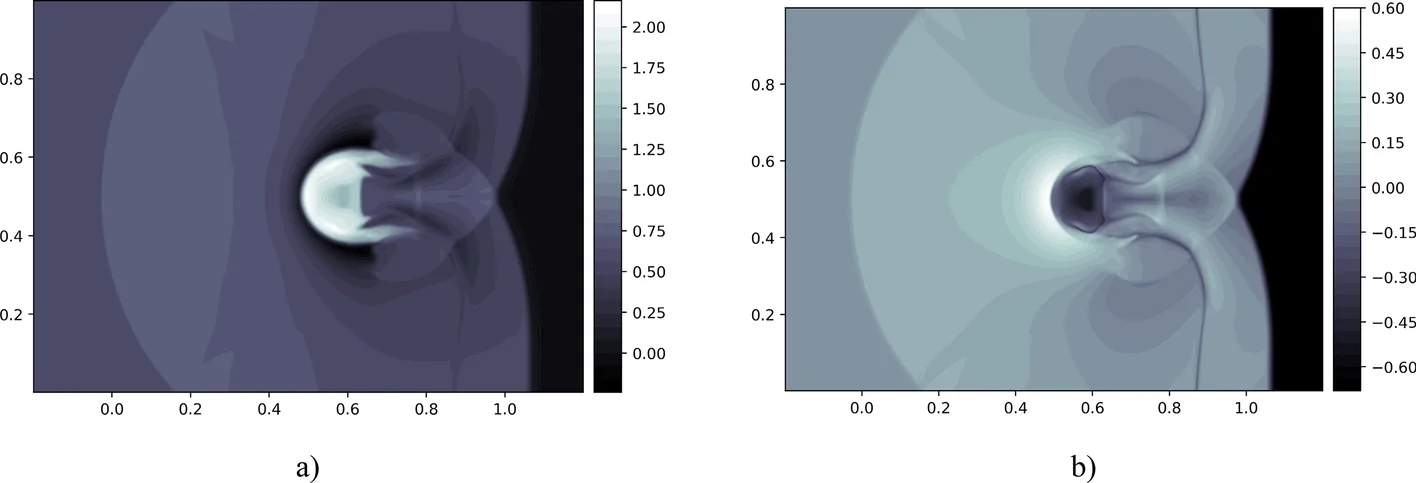
RMHD: shock-cloud interaction problem; **a** logarithm of density and **b** logarithm of magnetic pressure using the ninth-order accurate scheme

A cloud centered at (0.25, 0.5) with radius 0.15 is initialized in the domain. The cloud has the same states as the ambient fluid except for a higher density of 30 units. The adiabatic constant is set as γ=5/3. The simulation is run to a final time of 1.2 using a ninth-order accurate scheme on a grid that consists of 560×400 zones. Results are presented in Fig. 20. Figure 20a shows the obtained logarithm of the density and Fig. 20b shows the logarithm of the magnetic pressure at time *t* = 1.2. We see a close resemblance of the obtained results with the results presented in Wu and Shu [85]. The third-, fifth-, and seventh-order schemes also perform well on this problem; therefore, they are not shown here.

### Speed Comparisons Between FD and FV WENO Schemes

It is always interesting to ask whether there is a substantial speed advantage in the FD-WENO schemes (like the one presented here) and the FV-WENO schemes. An example of such an FV-WENO scheme would be the one for MHD in Balsara et al. [21]. Since that is a recently published work, we take that as a point of reference and catalog the speed differences within the context of numerical MHD. When considering speed, it is also important to recognize that FD methods do not suffer much from the curse of dimensionality, whereas FV methods do suffer a lot from the curse of dimensionality. As a result, a 2D FD scheme may have a certain speed advantage over a 2D FV scheme; however, a 3D FD scheme might have an even greater speed advantage over a 3D FV scheme. Even so, a fair discussion should not just stop there. We have to realize that an FV method may use a tensor product array of calls to the Riemann solver at the 2D boundary of a 3D zone; however, it is possible to use methods from van der Vegt and van der Ven [82, 83] to reduce the number of calls to the Riemann solver, thereby leading to some efficiencies. Analogous FD methods do not have the ability to exploit those efficiencies. Similarly, FV methods can make efficient use of ADER methods (Dumbser et al. [53, 54], Balsara et al. [30, 32]) to improve the efficiency of the timestepping. FD methods, because they do not have a volumetric reconstruction, cannot indeed draw on ADER approaches for timestepping. In particular, Balsara et al. [30] found that ADER methods offer a factor of two speed improvement relative to SSP-RK methods.

We mentioned all these factors in the previous paragraph, because every direct comparison between speeds of FD and FV codes of the same order of accuracy is always very nuanced. First, as explained before, it depends on whether the problem is 2D or 3D. Second, it depends on whether efficient tricks were used for flux evaluation or not. Third, it depends on whether ADER timestepping was used or not. Fourth, it is important to appreciate that the stencil size increases very dramatically with order and dimensionality for FV-WENO schemes, whereas this is not the case for FD-WENO schemes. The comparison we present here is between an FD-WENO scheme and an FV-WENO scheme that does not use the efficiencies of van der Vegt and van der Ven [82, 83] to reduce the number of calls to the Riemann solver. Furthermore, to keep the comparison fair, we use the same SSP-RK timestepping for both FD and FV schemes, but the FV scheme could have used ADER methods to improve its speed by at least a factor of two. We present both 2D speed comparisons and 3D speed comparisons, so that the effects of dimensionality can be factored in. Table 4 shows the results. We see that FD-WENO schemes do not suffer much from the curse of dimensionality as we go from 2D to 3D. We also see that in two dimensions, the FD methods have a 7- to 14-fold speed advantage over FV methods and that advantage becomes more pronounced at higher orders. In three dimensions, the FD methods have a 5- to 12-fold speed advantage over FV methods and, as before, that advantage becomes more pronounced at higher order. We also see that as we go from 2D to 3D, FD methods do not have as pronounced a degradation in speed as FV methods. In making Table 4, we used identical algorithmic elements between FD and FV methods just to keep the comparison fair. However, it should also be noted, in a spirit of fairness, that FV methods do have access to tricks, as shown in the previous paragraph, that would enable them to close the gap.

**Table 4:** It was made on a single core of an Intel(R) Xeon(R) Gold 6248R CPU @ 3.00 GHz with a GNU Fortran compiler (gcc v9.4). It shows the speeds, as measured in zones updated per second, by FD-WENO schemes and FV-WENO schemes at 3rd and 5th orders. 2D problems used meshes with 400^2^ zones, 3D problems used meshes with 128^3^ zones

Dimension (zones)	Order	FD-WENO zones/s	FV-WENO zones/s	Speed ratio
2D (400 × 400)	3rd	40 000	6 038	6.6
	5th	18 824	1 280	14.7
3D (128 × 128 × 128)	3rd	22 075	4 671	4.7
	5th	16 777	1 391	12.1

## Conclusions

AFD-WENO schemes have seen increasing prominence in recent years (Balsara et al. [19, 20]), because they can handle hyperbolic systems in conservation form as well as hyperbolic systems with non-conservative products. Moreover, they do so while preserving conservation when parts or all of the PDE system are in conservation form. They have also been extended to retain a PCP property, Bhoriya et al. [39]. Well-balancing can also be achieved within the context of AFD-WENO schemes, as shown by Xu and Shu [86]. The free stream preserving property can also be guaranteed for AFD-WENO schemes, as shown by Jiang et al. [64]. It is, therefore, natural to ask for AFD-WENO schemes that preserve the discrete divergence for PDE systems that have an involution property that preserves the divergence. Such involution preserving PDE systems encompass some very important application areas, such as CED, MHD, and RMHD.

By studying large classes of such PDEs, we find that for every vector field whose divergence has to be preserved on the mesh, we have an update equation that looks like Eq. (1). Such an update equation forms a sub-system of the overall PDE and a divergence-free or divergence-preserving update for such a system inevitably requires a Yee-style collocation of variables of the form shown in Fig. 1. If multiple vector fields have a divergence-preserving update, as is the case for CED, then the PDE system will have multiple copies of equations that look like Eq. (1), and all of those vector fields will have collocations of the type shown in Fig. 1. This brings us to the realization that a common solution strategy should be found for all these classes of involution-constrained PDEs. In all such cases, we find that stabilization of the numerical PDE requires a 2D upwinding, as shown in Figs. 3 and 4. The associated math, which is based on the multidimensional Riemann solver, was initially developed in Balsara [12, 13, 15]. In Sect. 3, we specialize the math for PDEs that have a sub-system of the type shown in Eq. (1). In the same section, Eqs. (12) and (20) give explicit formulae for such a multidimensional LLF and HLL Riemann solvers for involution-constrained PDEs that have a divergence-preserving constraint. Section 4 then provides a step-by-step plan for implementing the divergence-preserving AFD-WENO scheme. Section 5 shows the extra steps that have to be taken to achieve an efficient and time-explicit scheme that has the PCP property for divergence-preserving PDEs.

Section 6 shows the versatility of our method, because it shows that several rather different PDE systems with a divergence-based involution constraint can all be treated using the same formalism. For all these different involution-constrained PDE systems, we show that the method meets its design accuracy. Section 7 shows several stringent test problems associated with the same PDE systems. Some of these test problems are so stringent that their simulation requires the enforcement of a PCP condition. When solving such problems, we are introduced to another advantage of AFD-WENO schemes which is that it is just as convenient (and acceptable) to interpolate the primitive variables as it is to interpolate the conserved variables. This advantage is not shared by the classical FD-WENO method, where the right- and left-going fluxes can only be reconstructed in a dimension-by-dimension FV sense. Especially, for relativistic MHD, we find that only the primitive variables first documented in Balsara and Kim [28] will discriminate between flow velocities that are very close to the speed of light. (Of course, the primitive variables are indeed projected into the space of eigenvectors that are developed for primitive variables when carrying out 1D interpolation.) As a result, Sect. 5 of this paper, which describes PCP methods for involution-constrained PDEs, is very valuable when designing AFD-WENO schemes for very stringent MHD and relativistic MHD flows.

## Data Availability

All data that were used in the generation of the figures have been stored and available for later use.

## Appendix A Efficient FD 2D WENO-AO Interpolations

We describe a very efficient FD WENO-AO interpolation in two dimensions at third-, fifth-, and seventh-orders. While the math for ninth-order gives rise to very large expressions that are too long to document in a paper, the reader who understands this procedure can use a computer algebra system to easily make the extension to ninth and higher orders. We use this 2D WENO interpolation to obtain the electric field at the edge-centered locations. We use the tensor product of the following 1D Legendre polynomials, which span the interval $\left[ { - 1/2,1/2} \right]$, for the basis of the interpolated polynomial in two dimensions:

A1 $$\begin{aligned} \left\{ \begin{aligned} & L_{{0}} {(}x{\text{) = 1;}}\;L_{{1}} {(}x{) =\; }x{ ; }\; L_{{2}} {(}x{) = \;}x^{{2}} \, - \, \frac{{1}}{{{12}}}{ ; }\;L_{{3}} {(}x{) =\; }x^{{3}} \, - \, \frac{{3}}{{{20}}} \, x{ ; } \\& L_{{4}} {(}x{) =\; }x^{{4}} \, - \, \frac{{3}}{{{14}}} \, x^{{2}} { + }\frac{{3}}{{{560}}}{ ; }\; L_{5} {(}x{) =\; }x^{5} - \, \frac{5}{18}x^{3} + \, \frac{5}{336} \, x{ ; } \\& L_{6} {(}x{) =\; }x^{6} - \, \frac{15}{{44}}x^{4} + \, \frac{5}{176} \, x^{{2}} - \, \frac{5}{14\;784}{ ; } \\& L_{7} {(}x{) =\; }x^{7} - \frac{21}{{52}}x^{5} + \frac{105}{{2\;288}}x^{3} - \frac{35}{{27\;456}}x{ ;} \\& L_{8} {(}x{) =\; }x^{8} - \frac{7}{15}x^{6} + \frac{7}{104}x^{4} - \frac{7}{2\;288}x^{2} + \frac{7}{329\;472}.  \end{aligned} \right. \end{aligned}$$

We let “*r*” denote the order of accuracy of the interpolation; for example, an interpolation that is only based on $L_{{0}} {(}x{) = 1}$, $L_{{1}} {(}x{) = }\;x$, and $L_{{2}} {(}x{) = }\;x^{{2}} \, - 1/12$ corresponds to r=3.

### A.1 *r* = 3 FD-WENO Interpolation in Two Dimensions

The 2D interpolation polynomial of the third order is given by

A2 $$u(x,y) = u_{00} + u_{x} L_{1} (x) + u_{y} L_{1} (y) + u_{xx} L_{2} (x) + u_{yy} L_{2} (y) + u_{xy} L_{1} (x)L_{1} (y).$$

Figure 5 shows us five possible stencils that can each be used to evaluate the $u_{00} ,u_{x} ,u_{y} ,u_{xx} ,u_{yy},$ and $u_{xy}$ terms. The stencil $S_{1}^{{r_{3} }}$ denotes the right-up biased third-order accurate stencil, $S_{2}^{{r_{3} }}$ denotes the left-up biased third-order accurate stencil, $S_{3}^{{r_{3} }}$ denotes the left-down biased third-order accurate stencil, $S_{4}^{{r_{3} }}$ denotes the right-down biased third-order accurate stencil, and $S_{5}^{{r_{3} }}$ denotes the central third-order accurate stencil. For each stencil, we catalog expressions for the unknown terms $u_{00} ,u_{x} ,u_{y} ,u_{xx} ,u_{yy}$, and $u_{xy}$.

For the stencil $S_{1}^{{r_{3} }}$, we obtain

A3 $$\begin{aligned} \left\{ \begin{aligned} & u_{00} = \left( { - 2u_{0,1} + u_{0,2} + 26u_{0,0} - 2u_{1,0} + u_{2,0} } \right)/24, \\& u_{x} = \left( { - 3u_{0,0} + 4u_{1,0} - u_{2,0} } \right)/2, \\& u_{y} = \left( { - 3u_{0,0} + 4u_{0,1} - u_{0,2} } \right)/2, \\& u_{xx} = \left( {u_{0,0} - 2u_{1,0} + u_{2,0} } \right)/2, \\& u_{yy} = \left( {u_{0,0} - 2u_{0,1} + u_{0,2} } \right)/2, \\& u_{xy} = u_{1,1} + u_{0,0} - u_{0,1} - u_{1,0} . \end{aligned} \right. \end{aligned}$$

For the stencil $S_{2}^{{r_{3} }}$, we obtain

A4 $$\begin{aligned} \left\{ \begin{aligned} & u_{00} = \left( { - 2u_{0,1} + u_{0,2} + 26u_{0,0} - 2u_{ - 1,0} + u_{ - 2,0} } \right)/24, \\& u_{x} = \left( {3u_{0,0} - 4u_{ - 1,0} + u_{ - 2,0} } \right)/2, \\& u_{y} = \left( { - 3u_{0,0} + 4u_{0,1} - u_{0,2} } \right)/2, \\& u_{xx} = \left( {u_{0,0} - 2u_{ - 1,0} + u_{ - 2,0} } \right)/2, \\& u_{yy} = \left( {u_{0,0} - 2u_{0,1} + u_{0,2} } \right)/2, \\& u_{xy} = u_{0,1} + u_{ - 1,0} - u_{ - 1,1} - u_{0,0} . \end{aligned} \right. \end{aligned}$$

For the stencil $S_{3}^{{r_{3} }}$, we obtain

A5 $$\begin{aligned} \left\{ \begin{aligned} & u_{00} = \left( { - 2u_{0, - 1} + u_{0, - 2} + 26u_{0,0} - 2u_{ - 1,0} + u_{ - 2,0} } \right)/24, \\& u_{x} = \left( {3u_{0,0} - 4u_{ - 1,0} + u_{ - 2,0} } \right)/2, \\& u_{y} = \left( {3u_{0,0} - 4u_{0, - 1} + u_{0, - 2} } \right)/2, \\& u_{xx} = \left( {u_{0,0} - 2u_{ - 1,0} + u_{ - 2,0} } \right)/2, \\& u_{yy} = \left( {u_{0,0} - 2u_{0, - 1} + u_{0, - 2} } \right)/2, \\& u_{xy} = - u_{0, - 1} - u_{ - 1,0} + u_{ - 1, - 1} + u_{0,0} . \end{aligned} \right. \end{aligned}$$

For the stencil $S_{4}^{{r_{3} }}$, we obtain

A6 $$\begin{aligned} \left\{ \begin{aligned} & u_{00} = \left( { - 2u_{0, - 1} + u_{0, - 2} + 26u_{0,0} - 2u_{1,0} + u_{2,0} } \right)/24, \\& u_{x} = \left( { - 3u_{0,0} + 4u_{1,0} - u_{2,0} } \right)/2, \\& u_{y} = \left( {3u_{0,0} - 4u_{0, - 1} + u_{0, - 2} } \right)/2, \\& u_{xx} = \left( {u_{0,0} - 2u_{1,0} + u_{2,0} } \right)/2, \\& u_{yy} = \left( {u_{0,0} - 2u_{0, - 1} + u_{0, - 2} } \right)/2, \\& u_{xy} = u_{0, - 1} - u_{0,0} + u_{1,0} - u_{1, - 1} . \end{aligned} \right. \end{aligned}$$

For the stencil $S_{5}^{{r_{3} }}$, we obtain

A7 $$\begin{aligned} \left\{ \begin{aligned} & u_{00} = \left( {u_{0, - 1} + u_{0,1} + u_{ - 1,0} + u_{1,0} + 20u_{0,0} } \right)/24, \\& u_{x} = \left( {u_{1,0} - u_{ - 1,0} } \right)/2, \\& u_{y} = \left( {u_{0,1} - u_{0, - 1} } \right)/2, \\& u_{xx} = \left( {u_{1,0} - 2u_{0,0} + u_{ - 1,0} } \right)/2, \\& u_{yy} = \left( {u_{0,1} - 2u_{0,0} + u_{0, - 1} } \right)/2, \\& u_{xy} = u_{1,1} + u_{ - 1, - 1} - u_{ - 1,1} - u_{1, - 1} . \end{aligned} \right. \end{aligned}$$

The smoothness indicator (denoted by $\beta^{{r_{3} }}$) for the interpolated polynomial in Eq. (A1), written as a sum of perfect squares, is given by

$$\beta^{{r_{3} }} = u_{x}^{2} + u_{y}^{2} + \frac{13}{3}u_{xx}^{2} + \frac{13}{3}u_{yy}^{2} + \frac{7}{6}u_{xy}^{2} .$$

The smoothness indicator for the stencil $S_{1}^{{r_{3} }}$ is denoted by $\beta_{1}^{{r_{3} }}$, for the stencil $S_{2}^{{r_{3} }}$ is denoted by $\beta_{2}^{{r_{3} }}$, for the stencil $S_{3}^{{r_{3} }}$ is denoted by $\beta_{3}^{{r_{3} }}$, for the stencil $S_{4}^{{r_{3} }}$ is denoted by $\beta_{4}^{{r_{3} }}$, and for the stencil $S_{5}^{{r_{3} }}$ is denoted by $\beta_{5}^{{r_{3} }}$.

In designing a WENO scheme at third-order, we wish to make a non-linearly hybridized interpolation using stencils $S_{1}^{{r_{3} }} ,\;S_{2}^{{r_{3} }} \;,S_{3}^{{r_{3} }} \;,S_{4}^{{r_{3} }}$, and $S_{5}^{{r_{3} }}$. The interpolation is described by one parameter $\gamma_{\mathrm{Lo}} \in \left( {0,1} \right)$. We set $\gamma_{\mathrm{Lo}} = 0.85$. The linear weights for the stencils $S_{1}^{{r_{3} }} ,\;S_{2}^{{r_{3} }} \;,S_{3}^{{r_{3} }} \;,S_{4}^{{r_{3} }}$ are given by

A8 $$\gamma_{1}^{{r_{3} }} = \left( {1 - \gamma_{\mathrm{Lo}} } \right)/4{ ; }\; \gamma_{2}^{{r_{3} }} = \left( {1 - \gamma_{\mathrm{Lo}} } \right)/4{ ; }\; \gamma_{3}^{{r_{3} }} = \left( {1 - \gamma_{\mathrm{Lo}} } \right)/4{ ; }\; \gamma_{4}^{{r_{3} }} = \left( {1 - \gamma_{\mathrm{Lo}} } \right)/4.$$

The linear weight for the stencil $S_{5}^{{r_{3} }}$ is given by $\, \gamma_{5}^{{r_{3} }} = \gamma_{\mathrm{Lo}}$. Notice that for the linear weights, we have $\gamma_{1}^{{r_{3} }} + \gamma_{2}^{{r_{3} }} + \gamma_{3}^{{r_{3} }} + \gamma_{4}^{{r_{3} }} + \gamma_{5}^{{r_{3} }} = 1$. We see that when the central stencil $S_{5}^{{r_{3} }}$ is smooth, we want most of our interpolation to come from the central stencil, because it is the most stable choice when the solution is smooth. However, when a suitable comparison of the smoothness indicators shows that the central stencil is non-smooth, we want most (or all) of our interpolation to be weighted toward either the Right-Up biased stencil, the Left-Up biased stencil, the Left-Down biased stencil, or the Right-Down biased stencil, based on the smoothest of the interpolated polynomials.

To avoid loss of accuracy at critical points (Borges et al. [41]), we use the smoothness indicators to define

A9 $$\tau = \frac{1}{4}\left( {\left| {\beta_{5}^{{r_{3} }} - \beta_{1}^{{r_{3} }} } \right| + \left| {\beta_{5}^{{r_{3} }} - \beta_{2}^{{r_{3} }} } \right| + \left| {\beta_{5}^{{r_{3} }} - \beta_{3}^{{r_{3} }} } \right| + \left| {\beta_{5}^{{r_{3} }} - \beta_{4}^{{r_{3} }} } \right|} \right).$$

The unnormalized non-linear weights are given by

A10 $$\begin{aligned} \left\{ \begin{aligned} & w_{1}^{{r_{3} }} = \gamma_{1}^{{r_{3} }} \left( {1 + {{\tau^{2} } \mathord{\left/ {\vphantom {{\tau^{2} } {\left( {\beta_{1}^{{r_{3} }} + \varepsilon } \right)^{2} }}} \right. \kern-0pt} {\left( {\beta_{1}^{{r_{3} }} + \varepsilon } \right)^{2} }}} \right){ ; }\;w_{2}^{{r_{3} }} = \gamma_{2}^{{r_{3} }} \left( {1 + {{\tau^{2} } \mathord{\left/ {\vphantom {{\tau^{2} } {\left( {\beta_{2}^{{r_{3} }} + \varepsilon } \right)^{2} }}} \right. \kern-0pt} {\left( {\beta_{2}^{{r_{3} }} + \varepsilon } \right)^{2} }}} \right){ ; } \\& w_{3}^{{r_{3} }} = \gamma_{3}^{{r_{3} }} \left( {1 + {{\tau^{2} } \mathord{\left/ {\vphantom {{\tau^{2} } {\left( {\beta_{3}^{{r_{3} }} + \varepsilon } \right)^{2} }}} \right. \kern-0pt} {\left( {\beta_{3}^{{r_{3} }} + \varepsilon } \right)^{2} }}} \right){ ; }\;w_{4}^{{r_{3} }} = \gamma_{4}^{{r_{3} }} \left( {1 + {{\tau^{2} } \mathord{\left/ {\vphantom {{\tau^{2} } {\left( {\beta_{4}^{{r_{3} }} + \varepsilon } \right)^{2} }}} \right. \kern-0pt} {\left( {\beta_{4}^{{r_{3} }} + \varepsilon } \right)^{2} }}} \right){ ; } \\& w_{5}^{{r_{3} }} = \gamma_{5}^{{r_{3} }} \left( {1 + {{\tau^{2} } \mathord{\left/ {\vphantom {{\tau^{2} } {\left( {\beta_{5}^{{r_{3} }} + \varepsilon } \right)^{2} }}} \right. \kern-0pt} {\left( {\beta_{5}^{{r_{3} }} + \varepsilon } \right)^{2} }}} \right), \end{aligned} \right. \end{aligned}$$

where $\varepsilon = 10^{ - 12}$ is a small number to avoid the divisions by zero. The normalization of the non-linear weights is given by

A11 $$\begin{aligned} \left\{ \begin{aligned}& \overline{w}_{1}^{{r_{3} }} = {{w_{1}^{{r_{3} }} } \mathord{\left/ {\vphantom {{w_{1}^{{r_{3} }} } {\left( {w_{1}^{{r_{3} }} + w_{2}^{{r_{3} }} + w_{3}^{{r_{3} }} + w_{4}^{{r_{3} }} + w_{5}^{{r_{3} }} } \right)}}} \right. \kern-0pt} {\left( {w_{1}^{{r_{3} }} + w_{2}^{{r_{3} }} + w_{3}^{{r_{3} }} + w_{4}^{{r_{3} }} + w_{5}^{{r_{3} }} } \right)}}{ ; }\; \overline{w}_{2}^{{r_{3} }} = {{w_{2}^{{r_{3} }} } \mathord{\left/ {\vphantom {{w_{2}^{{r_{3} }} } {\left( {w_{1}^{{r_{3} }} + w_{2}^{{r_{3} }} + w_{3}^{{r_{3} }} + w_{4}^{{r_{3} }} + w_{5}^{{r_{3} }} } \right)}}} \right. \kern-0pt} {\left( {w_{1}^{{r_{3} }} + w_{2}^{{r_{3} }} + w_{3}^{{r_{3} }} + w_{4}^{{r_{3} }} + w_{5}^{{r_{3} }} } \right)}}{ ;} \\& \overline{w}_{3}^{{r_{3} }} = {{w_{3}^{{r_{3} }} } \mathord{\left/ {\vphantom {{w_{3}^{{r_{3} }} } {\left( {w_{1}^{{r_{3} }} + w_{2}^{{r_{3} }} + w_{3}^{{r_{3} }} + w_{4}^{{r_{3} }} + w_{5}^{{r_{3} }} } \right)}}} \right. \kern-0pt} {\left( {w_{1}^{{r_{3} }} + w_{2}^{{r_{3} }} + w_{3}^{{r_{3} }} + w_{4}^{{r_{3} }} + w_{5}^{{r_{3} }} } \right)}}{ ; }\; \overline{w}_{4}^{{r_{3} }} = {{w_{4}^{{r_{3} }} } \mathord{\left/ {\vphantom {{w_{4}^{{r_{3} }} } {\left( {w_{1}^{{r_{3} }} + w_{2}^{{r_{3} }} + w_{3}^{{r_{3} }} + w_{4}^{{r_{3} }} + w_{5}^{{r_{3} }} } \right)}}} \right. \kern-0pt} {\left( {w_{1}^{{r_{3} }} + w_{2}^{{r_{3} }} + w_{3}^{{r_{3} }} + w_{4}^{{r_{3} }} + w_{5}^{{r_{3} }} } \right)}}{ ;} \\& \overline{w}_{5}^{{r_{3} }} = {{w_{5}^{{r_{3} }} } \mathord{\left/ {\vphantom {{w_{5}^{{r_{3} }} } {\left( {w_{1}^{{r_{3} }} + w_{2}^{{r_{3} }} + w_{3}^{{r_{3} }} + w_{4}^{{r_{3} }} + w_{5}^{{r_{3} }} } \right)}}} \right. \kern-0pt} {\left( {w_{1}^{{r_{3} }} + w_{2}^{{r_{3} }} + w_{3}^{{r_{3} }} + w_{4}^{{r_{3} }} + w_{5}^{{r_{3} }} } \right)}}. \end{aligned} \right. \end{aligned}$$

Let $P_{i}^{{r_{3} }} {(}x,y{)}$ be the interpolated polynomial corresponding to the stencil $S_{i}^{{r_{3} }}$. The non-linearly hybridized third-order accurate WENO interpolation is given by

A12 $$P^{(3)} \left( {x,y} \right)\;{ = }\; \overline{w}_{1}^{{r_{3} }} \, P_{1}^{{r_{3} }} \left( {x,y} \right) \, + \, \overline{w}_{2}^{{r_{3} }} \, P_{2}^{{r_{3} }} \left( {x,y} \right) \, + \, \overline{w}_{3}^{{r_{3} }} \, P_{3}^{{r_{3} }} \left( {x,y} \right) \, + \, \overline{w}_{4}^{{r_{3} }} \, P_{4}^{{r_{3} }} \left( {x,y} \right) \, + \, \overline{w}_{5}^{{r_{3} }} \, P_{5}^{{r_{3} }} \left( {x,y} \right) \, {.}$$

This completes our description of the third-order 2D WENO interpolation.

### A.2 WENO-AO(5,3) Interpolation in Two Dimensions

The 2D WENO-AO(5,3) interpolation consists of a non-linear hybridization between a large, centered, fifth-order stencil denoted by $S_{1}^{r5}$ and the five smaller stencils described in Appendix A.1. The larger central fifth-order accurate stencil is shown in Fig. 6a. The fifth-order accurate 2D polynomial is given by

A13 $$\begin{aligned} u(x,y) =\; & u_{00} + u_{x} L_{1} (x) + u_{y} L_{1} (y) + u_{xx} L_{2} (x) + u_{yy} L_{2} (y) + u_{xy} L_{1} (x)L_{1} (y) \\& + u_{xxx} L_{3} (x){ + }u_{yyy} L_{3} (y) + u_{xxy} L_{2} (x)L_{1} (y){ + }u_{xyy} L_{1} (x)L_{2} (y) \\& + u_{xxxx} L_{4} (x) + u_{yyyy} L_{4} (y) + u_{xxxy} L_{3} (x)L_{1} (y) + u_{xyyy} L_{1} (x)L_{3} (y) + u_{xxyy} L_{2} (x)L_{2} (y). \end{aligned}$$

The large central fifth-order accurate stencil gives

$$\begin{gathered} u_{00} = (4\;636u_{0,0} + 288u_{0, - 1} - 17u_{0, - 2} + \, 288u_{0,1} - 17u_{0,2} + 288u_{ - 1,0} + 10u_{ - 1, - 1} + 10u_{ - 1,1} \hfill \\ \quad \quad \; \; - 17u_{ - 2,0} + 288u_{1,0} + 10u_{1, - 1} + 10u_{1,1} - 17u_{2,0} )/5\;760, \hfill \\ u_{x} \; = ( - 144u_{ - 1,0} - 5u_{ - 1, - 1} - 5u_{ - 1,1} + 17u_{ - 2,0} + 144u_{1,0} { + }5u_{1, - 1} + 5u_{1,1} - 17u_{2,0} )/240, \hfill \\ u_{y} \; = ( - 144u_{0, - 1} - 5u_{ - 1, - 1} - 5u_{1, - 1} + 17u_{0, - 2} + 144u_{0,1} { + }5u_{ - 1,1} + 5u_{1,1} - 17u_{0,2} )/240, \hfill \\ u_{xx} = ( - 374u_{0,0} - 14u_{0, - 1} - 14u_{0,1} + 198u_{ - 1,0} + 7u_{ - 1, - 1} + 7u_{ - 1,1} - 11u_{ - 2,0} + 198u_{1,0} \hfill \\ \quad \quad \; \; + 7u_{1, - 1} + 7u_{1,1} - 11u_{2,0} )/336, \hfill \\ u_{yy} = ( - 374u_{0,0} - 14u_{ - 1,0} - 14u_{1,0} + 198u_{0, - 1} + 7u_{ - 1, - 1} + 7u_{1, - 1} - 11u_{0, - 2} + 198u_{0,1} \hfill \\ \quad \quad \; \; + 7u_{ - 1,1} + 7u_{1,1} - 11u_{0,2} )/336, \hfill \\ u_{xy} = (188u_{ - 1, - 1} - 17u_{ - 1, - 2} - 188u_{ - 1,1} + 17u_{ - 1,2} - 17u_{ - 2,1} + 17u_{ - 2,1} - 188u_{1, - 1} + 17u_{1, - 2} \hfill \\ \quad \quad \; \; + \, 188u_{1,1} - 17u_{1,2} + 17u_{2, - 1} - 17u_{2,1} )/480, \hfill \\ u_{xxx} = (2u_{ - 1,0} - u_{ - 2,0} - 2u_{1,0} + u_{2,0} )/12, \hfill \\ u_{yyy} = (2u_{0, - 1} - u_{0, - 2} - 2u_{0,1} + u_{0,2} )/12, \hfill \\ u_{xxy} = (2u_{0, - 1} - 2u_{0,1} - u_{ - 1, - 1} + u_{ - 1,1} - u_{1, - 1} + u_{1,1} )/4, \hfill \\ u_{xyy} = (2u_{ - 1,0} - u_{ - 1, - 1} - u_{ - 1,1} - 2u_{1,0} + u_{1, - 1} + u_{1,1} )/4, \hfill \\ u_{xxxx} = (6u_{0,0} - 4u_{ - 1,0} + u_{ - 2,0} - 4u_{1,0} + u_{2,0} )/24, \hfill \\ u_{yyyy} = (6u_{0,0} - 4u_{0, - 1} + u_{0, - 2} - 4u_{0,1} + u_{0,2} )/24, \hfill \\ u_{xxxy} = ( - 2u_{ - 1, - 1} + 2u_{ - 1,1} + u_{ - 2, - 1} - u_{ - 2,1} + 2u_{1, - 1} - 2u_{1,1} - u_{2, - 1} + u_{2,1} )/24, \hfill \\ u_{xyyy} = ( - 2u_{ - 1, - 1} + u_{ - 1, - 2} + 2u_{ - 1,1} - u_{ - 1,2} + 2u_{1, - 1} - u_{1, - 2} - 2u_{1,1} + u_{1,2} )/24, \hfill \\ u_{xxyy} = (4u_{0,0} - 2u_{0, - 1} - 2u_{0,1} - 2u_{ - 1,0} + u_{ - 1, - 1} + u_{ - 1,1} - 2u_{1,0} + u_{1, - 1} + u_{1,1} )/4. \hfill \\ \end{gathered}$$

The smoothness indicator (denoted by $\beta^{{r_{5} }}$) for the interpolated polynomial in Eq. (A13), written as a sum of perfect squares, is given by

$$\begin{aligned} \beta^{{r_{5} }} =& \left( {u_{x} + \frac{{u_{xxx} }}{10}} \right)^{2} + \frac{13}{3}\left( {u_{xx} + \frac{123}{{455}}u_{xxxx} } \right)^{2} + \frac{781}{{20}}u_{xxx}^{2} + \frac{1\;421\;461}{{2\;275}}u_{xxxx}^{2} \\& { + }\left( {u_{y} + \frac{{u_{yyy} }}{10}} \right)^{2} + \frac{13}{3}\left( {u_{yy} + \frac{123}{{455}}u_{yyyy} } \right)^{2} + \frac{781}{{20}}u_{yyy}^{2} + \frac{1\;421\;461}{{2\;275}}u_{yyyy}^{2} \\& { + }\frac{7}{6}\left( {u_{xy} + \frac{13}{{140}}u_{xxxy} + \frac{13}{{140}}u_{xyyy} } \right)^{2} + \frac{47}{{10}}u_{xxy}^{2} + \frac{47}{{10}}u_{xyy}^{2} \\& { + }\frac{88\;841}{{2\;100}}u_{xxxy}^{2} { + }\frac{88\;841}{{2\;100}}u_{xyyy}^{2} + \frac{1}{16\;800}\left( {u_{xxxy} - u_{xyyy} } \right)^{2} + \frac{5\;083}{{270}}u_{xxyy}^{2} . \end{aligned}$$

In designing a WENO scheme at fifth order, we wish to make a non-linearly hybridized interpolation using the third-order stencils $S_{1}^{{r_{3} }} ,\;S_{2}^{{r_{3} }} \;,S_{3}^{{r_{3} }} \;,S_{4}^{{r_{3} }}$, $S_{5}^{{r_{3} }}$, and a fifth-order stencil $S_{1}^{r5}$. The interpolation is described by two parameters $\gamma_{\mathrm{Lo}} ,\gamma_{\mathrm{Hi}} \in \left( {0,1} \right)$. We set $\gamma_{\mathrm{Lo}} = \gamma_{\mathrm{Hi}} = 0.85$. The linear weights for the stencils $S_{1}^{{r_{3} }} ,\;S_{2}^{{r_{3} }} \;,S_{3}^{{r_{3} }} \;,S_{4}^{{r_{3} }}$ are given by

$$\begin{gathered} \gamma_{1}^{{r_{3} }} = \left( {1 - \gamma_{\mathrm{Hi}} } \right)\left( {1 - \gamma_{\mathrm{Lo}} } \right)/4{ ; }\; \gamma_{2}^{{r_{3} }} = \left( {1 - \gamma_{\mathrm{Hi}} } \right)\left( {1 - \gamma_{\mathrm{Lo}} } \right)/4{ ; } \hfill \\ \gamma_{3}^{{r_{3} }} = \left( {1 - \gamma_{\mathrm{Hi}} } \right)\left( {1 - \gamma_{\mathrm{Lo}} } \right)/4{ ; }\; \gamma_{4}^{{r_{3} }} = \left( {1 - \gamma_{\mathrm{Hi}} } \right)\left( {1 - \gamma_{\mathrm{Lo}} } \right)/4. \hfill \\ \end{gathered}$$

The linear weight for the stencil $S_{5}^{{r_{3} }}$ is given by $\, \gamma_{5}^{{r_{3} }} = \left( {1 - \gamma_{\mathrm{Hi}} } \right)\gamma_{\mathrm{Lo}}$. The linear weight for the stencil $S_{1}^{{r_{5} }}$ is given by $\, \gamma_{1}^{{r_{5} }} = \gamma_{\mathrm{Hi}}$. Notice that for the linear weights, we have $\gamma_{1}^{{r_{3} }} + \gamma_{2}^{{r_{3} }} + \gamma_{3}^{{r_{3} }} + \gamma_{4}^{{r_{3} }} + \gamma_{5}^{{r_{3} }} + \gamma_{1}^{{r_{5} }} = 1$. We see that when the fifth-order central stencil $S_{1}^{{r_{5} }}$ is smooth, we want most of our interpolation to come from the central stencil, because it is the most stable choice when the solution is smooth. However, when a suitable comparison of the smoothness indicators shows that the fifth-order central stencil is non-smooth, we want most (or all) of our interpolation to be weighted toward the third-order stencils. We denote the fifth-order central smoothness indicator by $\beta_{1}^{{r_{5} }}$.

To avoid loss of accuracy at critical points (Borges et al. [41]), we use the smoothness indicators to define $\tau = \frac{1}{5}\left( {\left| {\beta_{1}^{{r_{5} }} - \beta_{1}^{{r_{3} }} } \right| + \left| {\beta_{1}^{{r_{5} }} - \beta_{2}^{{r_{3} }} } \right| + \left| {\beta_{1}^{{r_{5} }} - \beta_{3}^{{r_{3} }} } \right| + \left| {\beta_{1}^{{r_{5} }} - \beta_{4}^{{r_{3} }} } \right| + \left| {\beta_{1}^{{r_{5} }} - \beta_{5}^{{r_{3} }} } \right|} \right).$

The unnormalized non-linear weights are given by

$$\begin{gathered} w_{1}^{{r_{3} }} = \gamma_{1}^{{r_{3} }} \left( {1 + {{\tau^{2} } \mathord{\left/ {\vphantom {{\tau^{2} } {\left( {\beta_{1}^{{r_{3} }} + \varepsilon } \right)^{2} }}} \right. \kern-0pt} {\left( {\beta_{1}^{{r_{3} }} + \varepsilon } \right)^{2} }}} \right){,}\; w_{2}^{{r_{3} }} = \gamma_{2}^{{r_{3} }} \left( {1 + {{\tau^{2} } \mathord{\left/ {\vphantom {{\tau^{2} } {\left( {\beta_{2}^{{r_{3} }} + \varepsilon } \right)^{2} }}} \right. \kern-0pt} {\left( {\beta_{2}^{{r_{3} }} + \varepsilon } \right)^{2} }}} \right){,} \hfill \\ w_{3}^{{r_{3} }} = \gamma_{3}^{{r_{3} }} \left( {1 + {{\tau^{2} } \mathord{\left/ {\vphantom {{\tau^{2} } {\left( {\beta_{3}^{{r_{3} }} + \varepsilon } \right)^{2} }}} \right. \kern-0pt} {\left( {\beta_{3}^{{r_{3} }} + \varepsilon } \right)^{2} }}} \right){,}\; w_{4}^{{r_{3} }} = \gamma_{4}^{{r_{3} }} \left( {1 + {{\tau^{2} } \mathord{\left/ {\vphantom {{\tau^{2} } {\left( {\beta_{4}^{{r_{3} }} + \varepsilon } \right)^{2} }}} \right. \kern-0pt} {\left( {\beta_{4}^{{r_{3} }} + \varepsilon } \right)^{2} }}} \right){,} \hfill \\ w_{5}^{{r_{3} }} = \gamma_{5}^{{r_{3} }} \left( {1 + {{\tau^{2} } \mathord{\left/ {\vphantom {{\tau^{2} } {\left( {\beta_{5}^{{r_{3} }} + \varepsilon } \right)^{2} }}} \right. \kern-0pt} {\left( {\beta_{5}^{{r_{3} }} + \varepsilon } \right)^{2} }}} \right){,}\;w_{1}^{{r_{5} }} = \gamma_{1}^{{r_{5} }} \left( {1 + {{\tau^{2} } \mathord{\left/ {\vphantom {{\tau^{2} } {\left( {\beta_{1}^{{r_{5} }} + \varepsilon } \right)^{2} }}} \right. \kern-0pt} {\left( {\beta_{1}^{{r_{5} }} + \varepsilon } \right)^{2} }}} \right), \hfill \\ \end{gathered}$$

where $\varepsilon = 10^{ - 12}$ is a small number to avoid the divisions by zero. The normalization of the non-linear weights is given by

$$\begin{gathered} \overline{w}_{1}^{{r_{3} }} = {{w_{1}^{{r_{3} }} } \mathord{\left/ {\vphantom {{w_{1}^{{r_{3} }} } {\left( {w_{1}^{{r_{3} }} + w_{2}^{{r_{3} }} + w_{3}^{{r_{3} }} + w_{4}^{{r_{3} }} + w_{5}^{{r_{3} }} + w_{1}^{{r_{5} }} } \right)}}} \right. \kern-0pt} {\left( {w_{1}^{{r_{3} }} + w_{2}^{{r_{3} }} + w_{3}^{{r_{3} }} + w_{4}^{{r_{3} }} + w_{5}^{{r_{3} }} + w_{1}^{{r_{5} }} } \right)}}{ ; }\; \overline{w}_{2}^{{r_{3} }} = {{w_{2}^{{r_{3} }} } \mathord{\left/ {\vphantom {{w_{2}^{{r_{3} }} } {\left( {w_{1}^{{r_{3} }} + w_{2}^{{r_{3} }} + w_{3}^{{r_{3} }} + w_{4}^{{r_{3} }} + w_{5}^{{r_{3} }} + w_{1}^{{r_{5} }} } \right)}}} \right. \kern-0pt} {\left( {w_{1}^{{r_{3} }} + w_{2}^{{r_{3} }} + w_{3}^{{r_{3} }} + w_{4}^{{r_{3} }} + w_{5}^{{r_{3} }} + w_{1}^{{r_{5} }} } \right)}}{ ; } \hfill \\ \overline{w}_{3}^{{r_{3} }} = {{w_{3}^{{r_{3} }} } \mathord{\left/ {\vphantom {{w_{3}^{{r_{3} }} } {\left( {w_{1}^{{r_{3} }} + w_{2}^{{r_{3} }} + w_{3}^{{r_{3} }} + w_{4}^{{r_{3} }} + w_{5}^{{r_{3} }} + w_{1}^{{r_{5} }} } \right)}}} \right. \kern-0pt} {\left( {w_{1}^{{r_{3} }} + w_{2}^{{r_{3} }} + w_{3}^{{r_{3} }} + w_{4}^{{r_{3} }} + w_{5}^{{r_{3} }} + w_{1}^{{r_{5} }} } \right)}}{ ; }\; \overline{w}_{4}^{{r_{3} }} = {{w_{4}^{{r_{3} }} } \mathord{\left/ {\vphantom {{w_{4}^{{r_{3} }} } {\left( {w_{1}^{{r_{3} }} + w_{2}^{{r_{3} }} + w_{3}^{{r_{3} }} + w_{4}^{{r_{3} }} + w_{5}^{{r_{3} }} + w_{1}^{{r_{5} }} } \right)}}} \right. \kern-0pt} {\left( {w_{1}^{{r_{3} }} + w_{2}^{{r_{3} }} + w_{3}^{{r_{3} }} + w_{4}^{{r_{3} }} + w_{5}^{{r_{3} }} + w_{1}^{{r_{5} }} } \right)}}{ ; } \hfill \\ \overline{w}_{5}^{{r_{3} }} = {{w_{5}^{{r_{3} }} } \mathord{\left/ {\vphantom {{w_{5}^{{r_{3} }} } {\left( {w_{1}^{{r_{3} }} + w_{2}^{{r_{3} }} + w_{3}^{{r_{3} }} + w_{4}^{{r_{3} }} + w_{5}^{{r_{3} }} + w_{1}^{{r_{5} }} } \right){ ; }\; \overline{w}_{1}^{{r_{5} }} = {{w_{1}^{{r_{5} }} } \mathord{\left/ {\vphantom {{w_{1}^{{r_{5} }} } {\left( {w_{1}^{{r_{3} }} + w_{2}^{{r_{3} }} + w_{3}^{{r_{3} }} + w_{4}^{{r_{3} }} + w_{5}^{{r_{3} }} + w_{1}^{{r_{5} }} } \right)}}} \right. \kern-0pt} {\left( {w_{1}^{{r_{3} }} + w_{2}^{{r_{3} }} + w_{3}^{{r_{3} }} + w_{4}^{{r_{3} }} + w_{5}^{{r_{3} }} + w_{1}^{{r_{5} }} } \right)}}}}} \right. \kern-0pt} {\left( {w_{1}^{{r_{3} }} + w_{2}^{{r_{3} }} + w_{3}^{{r_{3} }} + w_{4}^{{r_{3} }} + w_{5}^{{r_{3} }} + w_{1}^{{r_{5}}}} \right){ ; }\; \overline{w}_{1}^{{r_{5} }} = {{w_{1}^{{r_{5} }} } \mathord{\left/ {\vphantom {{w_{1}^{{r_{5} }} } {\left( {w_{1}^{{r_{3} }} + w_{2}^{{r_{3} }} + w_{3}^{{r_{3} }} + w_{4}^{{r_{3} }} + w_{5}^{{r_{3} }} + w_{1}^{{r_{5} }} } \right)}}} \right. \kern-0pt} {\left( {w_{1}^{{r_{3} }} + w_{2}^{{r_{3} }} + w_{3}^{{r_{3} }} + w_{4}^{{r_{3} }} + w_{5}^{{r_{3} }} + w_{1}^{{r_{5} }} } \right)}}}}. \hfill \\ \end{gathered}$$

Let $P_{i}^{{r_{3} }} {(}x,y{)}$ be the interpolated polynomial corresponding to the stencil $S_{i}^{{r_{3} }}$ and $P_{1}^{{r_{5} }} {(}x,y{)}$ be the interpolated polynomial corresponding to the stencil $S_{1}^{{r_{5} }}$. We denote the interpolated polynomial for WENO-AO(5,3) as $P^{{\mathrm{AO(5,3)}}} \left( {x,y} \right)$. Our task in this paragraph is to describe the construction of the order-preserving, non-linearly hybridized, fifth-order polynomial $P^{{\mathrm{AO(5,3)}}} \left( {x,y} \right)$. The non-linear weights should be combined in such a way that when all the smoothness indicators seem to have almost similar values, then only the higher order scheme is obtained. The non-linearly hybridized fifth-order accurate interpolation is given by

$$\begin{aligned} P^{{\mathrm{AO(5,3)}}} \left( x \right) =\; & \frac{{\overline{w}_{1}^{{r_{5} }} }}{{\gamma_{1}^{{r_{5} }} }}\left( \begin{aligned} &P_{1}^{{r_{5} }} \left( {x,y} \right) \, - \, \gamma_{1}^{{r_{3} }} \, P_{1}^{{r_{3} }} \left( {x,y} \right) - \, \gamma_{2}^{{r_{3} }} \, P_{2}^{{r_{3} }} \left( {x,y} \right) - \, \gamma_{3}^{{r_{3} }} \, P_{3}^{{r_{3} }} \left( {x,y} \right) \\& - \, \gamma_{4}^{{r_{3} }} \, P_{4}^{{r_{3} }} \left( {x,y} \right) - \, \gamma_{5}^{{r_{3} }} \, P_{5}^{{r_{3} }} \left( {x,y} \right) \, \hfill \\ \end{aligned} \right) \\& \, + \, \overline{w}_{1}^{{r_{3} }} \, P_{1}^{{r_{3} }} \left( {x,y} \right) \, + \, \overline{w}_{2}^{{r_{3} }} \, P_{2}^{{r_{3} }} \left( {x,y} \right) \, + \, \overline{w}_{3}^{{r_{3} }} \, P_{3}^{{r_{3} }} \left( {x,y} \right) \, + \, \overline{w}_{4}^{{r_{3} }} \, P_{4}^{{r_{3} }} \left( {x,y} \right) \, + \, \overline{w}_{5}^{{r_{3} }} \, P_{5}^{{r_{3} }} \left( {x,y} \right). \end{aligned}$$

This completes our description of the fifth-order 2D WENO-AO(5,3) interpolation.

### A.3 WENO-AO(7,5,3) Interpolation in Two Dimensions

The 2D WENO-AO(7,5,3) interpolation consists of a non-linear hybridization between a large, centered, seventh-order stencil denoted by $S_{1}^{r7}$, an intermediate central fifth-order stencil denoted by $S_{1}^{r5}$, and the five smaller stencils described in Appendix A.1. The larger central seventh-order accurate stencil is shown in Fig. 6b and the intermediate fifth-order central stencil is shown in Fig. 6a. The seventh-order accurate 2D polynomial is given by

A14 $$\begin{aligned} u(x,y) =\; & u_{00} + u_{x} L_{1} (x) + u_{y} L_{1} (y) + u_{xx} L_{2} (x) + u_{yy} L_{2} (y) + u_{xy} L_{1} (x)L_{1} (y) \\& + u_{xxx} L_{3} (x) + \, u_{yyy} L_{3} (y) + u_{xxy} L_{2} (x)L_{1} (y) + \, u_{xyy} L_{1} (x)L_{2} (y) \\& + u_{xxxx} L_{4} (x) + u_{yyyy} L_{4} (y) + u_{xxxy} L_{3} (x)L_{1} (y) + u_{xyyy} L_{1} (x)L_{3} (y) + u_{xxyy} L_{2} (x)L_{2} (y) \\& + u_{xxxxx} L_{5} (x) + u_{yyyyy} L_{5} (y) + u_{xxxxy} L_{4} (x)L_{1} (y) + u_{xyyyy} L_{1} (x)L_{4} (y) \\& + u_{xxxyy} L_{3} (x)L_{2} (y) + u_{xxyyy} L_{2} (x)L_{3} (y) \\& + u_{xxxxxx} L_{6} (x) + u_{yyyyyy} L_{6} (y) + u_{xxxxxy} L_{5} (x)L_{1} (y) + u_{xyyyyy} L_{1} (x)L_{5} (y) \\& + u_{xxxxyy} L_{4} (x)L_{2} (y) + u_{xxyyyy} L_{2} (x)L_{4} (y) + u_{xxxyyy} L_{3} (x)L_{3} (y). \end{aligned}$$

The large central stencil gives

$$\begin{aligned} \left\{ \begin{aligned} & u_{00} = (767\;024u_{0,0} + 52\;223u_{0, - 1} - 4\;820u_{0, - 2} + 367u_{0, - 3} + 52\;223u_{0,1} - 4\;820u_{0,2} + 367u_{0,3} \\& \quad \quad \; \; + 52\;223u_{ - 1,0} + 2\;632u_{ - 1, - 1} - 119u_{ - 1, - 2} + 2\;632u_{ - 1,1} - 119u_{ - 1,2} - 4\;820u_{ - 2,0} - 119u_{ - 2, - 1} \\& \quad \quad \; \; - 119u_{ - 2,1} + 367u_{ - 3,0} + 52\;223u_{1,0} + 2\;632u_{1, - 1} - 119u_{1, - 2} + 2\;632u_{1,1} - 119u_{1,2} - 4\;820u_{2,0} \\& \quad \quad \; \; - 119u_{2, - 1} - 119u_{2,1} + 367u_{3,0} )/967\;680, \\& u_{x} = ( - 52\;223u_{ - 1,0} - 2\;632u_{ - 1, - 1} + 119u_{ - 1, - 2} - 2\;632u_{ - 1,1} + 119u_{ - 1,2} + 9\;640u_{ - 2,0} + 238u_{ - 2, - 1} \\& \quad \quad \; \; + 238u_{ - 2,1} - 1\;101u_{ - 3,0} + 52\;223u_{1,0} + 2\;632u_{1, - 1} - 119u_{1, - 2} + 2\;632u_{1,1} - 119u_{1,2} - 9\;640u_{2,0} \\& \quad \quad \; \; - 238u_{2, - 1} - 238u_{2,1} + 1\;101u_{3,0} )/80\;640, \\& u_{y} = ( - 52\;223u_{0, - 1} + 9\;640u_{0, - 2} - 1\;101u_{0, - 3} + 52\;223u_{0,1} - 9\;640u_{0,2} + 1\;101u_{0,3} - 2\;632u_{ - 1, - 1} \\& \quad \quad \; \; + 238u_{ - 1, - 2} + 2\;632u_{ - 1,1} - 238u_{ - 1,2} + 119u_{ - 2, - 1} - 119u_{ - 2,1} - 2\;632u_{1, - 1} + 238u_{1, - 2} \\& \quad \quad \; \; + 2\;632u_{1,1} - 238u_{1,2} + 119u_{2, - 1} - 119u_{2,1} )/80\;640, \end{aligned} \right. \end{aligned}$$

$$\begin{aligned} \left\{ \begin{aligned} & u_{xx} = ( - 93\;672u_{0,0} - 4\;972u_{0, - 1} + 238u_{0, - 2} - 4\;972u_{0,1} + 238u_{0,2} + 50\;921u_{ - 1,0} + 2\;596u_{ - 1, - 1} \\& \quad \quad \; \; - 119u_{ - 1, - 2} + 2\;596u_{ - 1,1} - 119u_{ - 1,2} - 4\;418u_{ - 2,0} - 110u_{ - 2, - 1} - 110u_{ - 2,1} + 333u_{ - 3,0} \\& \quad \quad \; \; + 50\;921u_{1,0} + 2\;596u_{1, - 1} - 119u_{1, - 2} + 2\;596u_{1,1} - 119u_{1,2} - 4\;418u_{2,0} - 110u_{2, - 1} - 110u_{2,1} \\& \quad \quad \; \; + 333u_{3,0} )/80\;640, \\& u_{yy} = ( - 93\;672u_{0,0} + 50\;921u_{0, - 1} - 4\;418u_{0, - 2} + 333u_{0, - 3} + 50\;921u_{0,1} - 4\;418u_{0,2} + 333u_{0,3} \\& \quad \quad \; \; - 4\;972u_{ - 1,0} + 2\;596u_{ - 1, - 1} - 110u_{ - 1, - 2} + 2\;596u_{ - 1,1} - 110u_{ - 1,2} + 238u_{ - 2,0} - 119u_{ - 2, - 1} \\& \quad \quad \; \; - 119u_{ - 2,1} - 4\;972u_{1,0} + 2\;596u_{1, - 1} - 110u_{1, - 2} + 2\;596u_{1,1} - 110u_{1,2} + 238u_{2,0} - 119u_{2, - 1} \\& \quad \quad \; \; - 119u_{2,1} )/80\;640, \\& u_{xy} = (387\;074u_{ - 1, - 1} - 58\;672u_{ - 1, - 2} + 5\;505u_{ - 1, - 3} - 387\;074u_{ - 1,1} + 58\;672u_{ - 1,2} - 5\;505u_{ - 1,3} \\& \quad \quad \; \; - 58\;672u_{ - 2, - 1} + 4\;046u_{ - 2, - 2} + 58\;672u_{ - 2,1} - 4\;046u_{ - 2,2} + 5\;505u_{ - 3, - 1} - 5\;505u_{ - 3,1} \\& \quad \quad \; \; - 387\;074u_{1, - 1} + 58\;672u_{1, - 2} - 5\;505u_{1, - 3} + 387\;074u_{1,1} - 58\;672u_{1,2} + 5\;505u_{1,3} \\& \quad \quad \; \; + 58\;672u_{2, - 1} - 4\;046u_{2, - 2} - 58\;672u_{2,1} + 4\;046u_{2,2} - 5\;505u_{3, - 1} + 5\;505u_{3,1} )/806\;400, \end{aligned} \right. \end{aligned}$$

$$\begin{aligned} \left\{ \begin{aligned} & u_{xxx} = (217u_{ - 1,0} + 6u_{ - 1, - 1} + 6u_{ - 1,1} - 134u_{ - 2,0} - 3u_{ - 2, - 1} - 3u_{ - 2,1} + 17u_{ - 3,0} - 217u_{1,0} - 6u_{1, - 1} \\& \quad \quad \; \; - 6u_{1,1} + 134u_{2,0} + 3u_{2, - 1} + 3u_{2,1} - 17u_{3,0} )/864, \\& u_{yyy} = (217u_{0, - 1} - 134u_{0, - 2} + 17u_{0, - 3} - 217u_{0,1} + 134u_{0,2} - 17u_{0,3} + 6u_{ - 1, - 1} - 3u_{ - 1, - 2} - 6u_{ - 1,1} \\& \quad \quad \; \; + 3u_{ - 1,2} + 6u_{1, - 1} - 3u_{1, - 2} - 6u_{1,1} + 3u_{1,2} )/864, \\& u_{xxy} = (2\;486u_{0, - 1} - 238u_{0, - 2} - 2\;486u_{0,1} + 238u_{0,2} - 1\;298u_{ - 1, - 1} + 119u_{ - 1, - 2} + 1\;298u_{ - 1,1} \\& \quad \quad \; \; - 119u_{ - 1,2} + 55u_{ - 2, - 1} - 55u_{ - 2,1} - 1\;298u_{1, - 1} + 119u_{1, - 2} + 1\;298u_{1,1} - 119u_{1,2} + 55u_{2, - 1} \\& \quad \quad \; \; - 55u_{2,1} )/3\;360, \\& u_{xyy} = (2\;486u_{ - 1,0} - 1\;298u_{ - 1, - 1} + 55u_{ - 1, - 2} - 1\;298u_{ - 1,1} + 55u_{ - 1,2} - 238u_{ - 2,0} + 119u_{ - 2, - 1} \\& \quad \quad \; \; + 119u_{ - 2,1} - 2\;486u_{1,0} + 1\;298u_{1, - 1} - 55u_{1, - 2} + 1\;298u_{1,1} - 55u_{1,2} + 238u_{2,0} - 119u_{2, - 1} \\& \quad \quad \; \; - 119u_{2,1} )/3\;360, \end{aligned} \right. \end{aligned}$$

$$\begin{aligned} \left\{ \begin{aligned} & u_{xxxx} = (2\;272u_{0,0} + 66u_{0, - 1} + 66u_{0,1} - 1\;583u_{ - 1,0} - 44u_{ - 1, - 1} - 44u_{ - 1,1} + 488u_{ - 2,0} + 11u_{ - 2, - 1} \\& \quad \quad \quad \; \; + 11u_{ - 2,1} - 41u_{ - 3,0} - 1\;583u_{1,0} - 44u_{1, - 1} - 44u_{1,1} + 488u_{2,0} + 11u_{2, - 1} + 11u_{2,1} - 41u_{3,0} )/6\;336, \\& u_{yyyy} = (2\;272u_{0,0} - 1\;583u_{0, - 1} + 488u_{0, - 2} - 41u_{0, - 3} - 1\;583u_{0,1} + 488u_{0,2} - 41u_{0,3} + 66u_{ - 1,0} \\& \quad \quad \quad \; \; - 44u_{ - 1, - 1} + 11u_{ - 1, - 2} - 44u_{ - 1,1} + 11u_{ - 1,2} + 66u_{1,0} - 44u_{1, - 1} + 11u_{1, - 2} - 44u_{1,1} + 11u_{1,2} )/6\;336, \\& u_{xxxy} = ( - 1\;349u_{ - 1, - 1} + 102u_{ - 1, - 2} + 1\;349u_{ - 1,1} - 102u_{ - 1,2} + 802u_{ - 2, - 1} - 51u_{ - 2, - 2} - 802u_{ - 2,1} \\& \quad \quad \quad \; \; + 51u_{ - 2,2} - 85u_{ - 3, - 1} + 85u_{ - 3,1} + 1\;349u_{1, - 1} - 102u_{1, - 2} - 1\;349u_{1,1} + 102u_{1,2} - 802u_{2, - 1} \\& \quad \quad \quad \; \; + 51u_{2, - 2} + 802u_{2,1} - 51u_{2,2} + 85u_{3, - 1} - 85u_{3,1} )/8\;640, \\& u_{xyyy} = ( - 1\;349u_{ - 1, - 1} + 802u_{ - 1, - 2} - 85u_{ - 1, - 3} + 1\;349u_{ - 1,1} - 802u_{ - 1,2} + 85u_{ - 1,3} + 102u_{ - 2, - 1} - 51u_{ - 2, - 2} \\& \quad \quad \quad \; \; - 102u_{ - 2,1} + 51u_{ - 2,2} + 1\;349u_{1, - 1} - 802u_{1, - 2} + 85u_{1, - 3} - 1\;349u_{1,1} + 802u_{1,2} - 85u_{1,3} - 102u_{2, - 1} \\& \quad \quad \quad \; \; + 51u_{2, - 2} + 102u_{2,1} - 51u_{2,2} )/8\;640, \\& u_{xxyy} = (936u_{0,0} - 490u_{0, - 1} + 22u_{0, - 2} - 490u_{0,1} + 22u_{0,2} - 490u_{ - 1,0} + 256u_{ - 1, - 1} - 11u_{ - 1, - 2} + 256u_{ - 1,1} \\& \quad \quad \quad \; \; - 11u_{ - 1,2} + 22u_{ - 2,0} - 11u_{ - 2, - 1} - 11u_{ - 2,1} - 490u_{1,0} + 256u_{1, - 1} - 11u_{1, - 2} + 256u_{1,1} - 11u_{1,2} + 22u_{2,0} \\& \quad \quad \quad \; \; - 11u_{2, - 1} - 11u_{2,1} )/672, \end{aligned} \right. \end{aligned}$$

$$\begin{aligned} \left\{ \begin{aligned} & u_{xxxxx} = ( - 5u_{ - 1,0} + 4u_{ - 2,0} - u_{ - 3,0} + 5u_{1,0} - 4u_{2,0} + u_{3,0} )/240, \\& u_{yyyyy} = ( - 5u_{0, - 1} + 4u_{0, - 2} - u_{0, - 3} + 5u_{0,1} - 4u_{0,2} + u_{0,3} )/240, \\& u_{xxxxy} = ( - 6u_{0, - 1} + 6u_{0,1} + 4u_{ - 1, - 1} - 4u_{ - 1,1} - u_{ - 2, - 1} + u_{ - 2,1} + 4u_{1, - 1} - 4u_{1,1} - u_{2, - 1} + u_{2,1} )/48, \\& u_{xyyyy} = ( - 6u_{ - 1,0} + 4u_{ - 1, - 1} - u_{ - 1, - 2} + 4u_{ - 1,1} - u_{ - 1,2} + 6u_{1,0} - 4u_{1, - 1} + u_{1, - 2} - 4u_{1,1} + u_{1,2} )/48, \\& u_{xxxyy} = ( - 4u_{ - 1,0} + 2u_{ - 1, - 1} + 2u_{ - 1,1} + 2u_{ - 2,0} - u_{ - 2, - 1} - u_{ - 2,1} + 4u_{1,0} - 2u_{1, - 1} - 2u_{1,1} - 2u_{2,0} + u_{2, - 1} + u_{2,1} )/24, \\& u_{xxyyy} = ( - 4u_{0, - 1} + 2u_{0, - 2} + 4u_{0,1} - 2u_{0,2} + 2u_{ - 1, - 1} - u_{ - 1, - 2} - 2u_{ - 1,1} + u_{ - 1,2} + 2u_{1, - 1} - u_{1, - 2} - 2u_{1,1} + u_{1,2} )/24, \end{aligned} \right. \end{aligned}$$

$$\begin{aligned} \left\{ \begin{aligned} & u_{xxxxxx} = ( - 20u_{0,0} + 15u_{ - 1,0} - 6u_{ - 2,0} + u_{ - 3,0} + 15u_{1,0} - 6u_{2,0} + u_{3,0} )/720, \\& u_{yyyyyy} = ( - 20u_{0,0} + 15u_{0, - 1} - 6u_{0, - 2} + u_{0, - 3} + 15u_{0,1} - 6u_{0,2} + u_{0,3} )/720, \\& u_{xxxxxy} = (5u_{ - 1, - 1} - 5u_{ - 1,1} - 4u_{ - 2, - 1} + 4u_{ - 2,1} + u_{ - 3, - 1} - u_{ - 3,1} - 5u_{1, - 1} + 5u_{1,1} + 4u_{2, - 1} - 4u_{2,1} - u_{3, - 1} + u_{3,1} )/480, \\& u_{xyyyyy} = (5u_{ - 1, - 1} - 4u_{ - 1, - 2} + u_{ - 1, - 3} - 5u_{ - 1,1} + 4u_{ - 1,2} - u_{ - 1,3} - 5u_{1, - 1} + 4u_{1, - 2} - u_{1, - 3} + 5u_{1,1} - 4u_{1,2} + u_{1,3} )/480, \\& u_{xxxxyy} = ( - 12u_{0,0} + 6u_{0, - 1} + 6u_{0,1} + 8u_{ - 1,0} - 4u_{ - 1, - 1} - 4u_{ - 1,1} - 2u_{ - 2,0} + u_{ - 2, - 1} + u_{ - 2,1} + 8u_{1,0} - 4u_{1, - 1} - 4u_{1,1} \\& \qquad \quad \quad - 2u_{2,0} + u_{2, - 1} + u_{2,1} )/48, \\& u_{xxyyyy} = ( - 12u_{0,0} + 8u_{0, - 1} - 2u_{0, - 2} + 8u_{0,1} - 2u_{0,2} + 6u_{ - 1,0} - 4u_{ - 1, - 1} + u_{ - 1, - 2} - 4u_{ - 1,1} + u_{ - 1,2} + 6u_{1,0} - 4u_{1, - 1} \\& \qquad \quad \quad + u_{1, - 2} - 4u_{1,1} + u_{1,2} )/48, \\& u_{xxxyyy} = (4u_{ - 1, - 1} - 2u_{ - 1, - 2} - 4u_{ - 1,1} + 2u_{ - 1,2} - 2u_{ - 2, - 1} + u_{ - 2, - 2} + 2u_{ - 2,1} - u_{ - 2,2} - 4u_{1, - 1} + 2u_{1, - 2} + 4u_{1,1} - 2u_{1,2} \\& \qquad \quad \quad + 2u_{2, - 1} - u_{2, - 2} - 2u_{2,1} + u_{2,2} )/144. \end{aligned} \right. \end{aligned}$$

The smoothness indicator (denoted by $\beta^{{r_{7} }}$) for the interpolated polynomial in Eq. (A14), written as a sum of perfect squares, is given by

$$\begin{aligned} \beta^{{r_{7} }} = & \; \left( {u_{x} + \frac{{u_{xxx} }}{10} + \frac{{u_{xxxxx} }}{126}} \right)^{2} + \left( {u_{y} + \frac{{u_{yyy} }}{10} + \frac{{u_{yyyyy} }}{126}} \right)^{2} \\& + \frac{13}{3}\left( {u_{xx} + \frac{123}{{455}}u_{xxxx} + \frac{85}{{2\;002}}u_{xxxxxx} } \right)^{2} + \frac{13}{3}\left( {u_{yy} + \frac{123}{{455}}u_{yyyy} + \frac{85}{{2\;002}}u_{yyyyyy} } \right)^{2} \\& + \frac{7}{6}\left( {u_{xy} + \frac{13}{{140}}u_{xxxy} + \frac{13}{{140}}u_{xyyy} + \frac{13}{{1\;764}}u_{xxxxxy} + \frac{13}{{1\;764}}u_{xyyyyy} + \frac{3}{350}u_{xxxyyy} } \right)^{2} \\& + \frac{781}{{20}}\left( {u_{xxx} + \frac{26\;045}{{49\;203}}u_{xxxxx} } \right)^{2} + \frac{781}{{20}}\left( {u_{yyy} + \frac{26\;045}{{49\;203}}u_{yyyyy} } \right)^{2} \\& + \frac{47}{{10}}\left( {u_{xxy} + \frac{533}{{1\;974}}u_{xxxxy} + \frac{781}{{8\;460}}u_{xxyyy} } \right)^{2} + \frac{47}{{10}}\left( {u_{xyy} + \frac{533}{{1\;974}}u_{xyyyy} + \frac{781}{{8\;460}}u_{xxxyy} } \right)^{2} \\& + \frac{1\;421\;461}{{2\;275}}\left( {u_{xxxx} + \frac{81\;596\;225}{{93\;816\;426}}u_{xxxxxx} } \right)^{2} + \frac{1\;421\;461}{{2\;275}}\left( {u_{yyyy} + \frac{81\;596\;225}{{93\;816\;426}}u_{yyyyyy} } \right)^{2} \\& + \frac{88\;841}{{2\;100}}\left( {u_{xxxy} + \frac{2\;100}{{177\;682}}\left( {\frac{4\;740\;203}{{105\;840}}u_{xxxxxy} + \frac{109\;343}{{14\;000}}u_{xxxyyy} - \frac{1}{105\;840}u_{xyyyyy} } \right)} \right)^{2} \\& + \frac{88\;841}{{2\;100}}\left( {u_{xyyy} + \frac{2\;100}{{177\;682}}\left( {\frac{4\;740\;203}{{105\;840}}u_{xyyyyy} + \frac{109\;343}{{14\;000}}u_{xxxyyy} - \frac{1}{105\;840}u_{xxxxxy} } \right)} \right)^{2} \\& + \frac{1}{{{16\;800}}}\left( {u_{xxxy} - u_{xyyy} } \right)^{2} + \frac{{{5\;083}}}{{{270}}}\left( {u_{xxyy} + \frac{{{270}}}{{{10\;166}}}\left( {\frac{{{32\;021}}}{{{3\;150}}}u_{xxxxyy} + \frac{{{32\;021}}}{{{3\;150}}}u_{xxyyyy} } \right)} \right)^{2} \\& + \frac{{{21\;520\;059\;541}}}{{{1\;377\;684}}}u_{xxxxx}^{2} + \frac{{{21\;520\;059\;541}}}{{{1\;377\;684}}}u_{yyyyy}^{2} \\& + \frac{{{2\;805\;965\;789}}}{{{4\;145\;400}}}\left( {u_{xxxxy} + \frac{{{287}}}{{{16\;835\;794\;734}}}u_{xxyyy} } \right)^{2} + \frac{{{2\;805\;965\;789}}}{{{4\;145\;400}}}\left( {u_{xyyyy} + \frac{{{287}}}{{{16\;835\;794\;734}}}u_{xxxyy} } \right)^{2} \\& + \frac{{{13\;478\;684\;678\;301\;203}}}{{{79\;549\;130\;118\;150}}}u_{xxxyy}^{2} + \frac{{{13\;478\;684\;678\;301\;203}}}{{{79\;549\;130\;118\;150}}}u_{xxyyy}^{2} \\& + \frac{{{15\;510\;384\;942\;580\;921}}}{{{27\;582\;029\;244}}}u_{xxxxxx}^{2} + \frac{{{15\;510\;384\;942\;580\;921}}}{{{27\;582\;029\;244}}}u_{yyyyyy}^{2} \\& + \frac{{{150\;025\;516\;743\;043}}}{{{8\;865\;621\;072}}}\left( {u_{xxxxxy} + \frac{{{278\;487}}}{{{1\;200\;204\;133\;944\;344}}}u_{xxxyyy} } \right)^{2} \\& + \frac{{{150\;025\;516\;743\;043}}}{{{8\;865\;621\;072}}}\left( {u_{xyyyyy} + \frac{{{278\;487}}}{{{1\;200\;204\;133\;944\;344}}}u_{xxxyyy} } \right)^{2} \\& + \frac{{{1\;461\;613}}}{{{315\;938\;496\;384}}}\left( {u_{xxxxxy} + u_{xyyyyy} } \right)^{2} + \frac{{{2\;278\;884\;634\;589}}}{{{840\;601\;125}}}u_{xxxxyy}^{2} + \frac{{{2\;278\;884\;634\;589}}}{{{840\;601\;125}}}u_{xxyyyy}^{2} \\& + \frac{{{1\;681}}}{{{747\;201\;000}}}\left( {u_{xxxxyy} - u_{xxyyyy} } \right)^{2} + \frac{{{1\;821\;183\;508\;634\;515\;119\;164\;258\;353}}}{{{1\;194\;226\;157\;193\;994\;011\;404\;800}}}u_{xxxyyy}^{2} . \end{aligned}$$

In designing a WENO scheme at seventh order, we wish to make a non-linearly hybridized interpolation using the third-order stencils $S_{1}^{{r_{3} }} ,\;S_{2}^{{r_{3} }} \;,S_{3}^{{r_{3} }} \;,S_{4}^{{r_{3} }}$, $S_{5}^{{r_{3} }}$, an intermediate central fifth-order stencil $S_{1}^{{r_{5} }}$, and a large central seventh-order stencil $S_{1}^{{r_{7} }}$. We would like to have a scheme that produces a smoother transition from seventh to fifth order and then from fifth to third order. The interpolation is described by three parameters $\gamma_{\mathrm{Lo}} ,\gamma_{\mathrm{Avg}} ,\gamma_{\mathrm{Hi}} \in \left( {0,1} \right)$. We set $\gamma_{\mathrm{Lo}} = \gamma_{\mathrm{Avg}} = \gamma_{\mathrm{Hi}} = 0.85$. The linear weights for the stencils $S_{1}^{{r_{3} }} ,\;S_{2}^{{r_{3} }} \;,S_{3}^{{r_{3} }} \;,S_{4}^{{r_{3} }}$ are given by

$$\begin{gathered} \gamma_{1}^{{r_{3} }} = \left( {1 - \gamma_{\mathrm{Hi}} } \right)\left( {1 - \gamma_{\mathrm{Avg}} } \right)\left( {1 - \gamma_{\mathrm{Lo}} } \right)/4{ ; }\;\gamma_{2}^{{r_{3} }} = \left( {1 - \gamma_\text{{Hi}} } \right)\left( {1 - \gamma_{\mathrm{Avg}} } \right)\left( {1 - \gamma_{\mathrm{Lo}} } \right)/4{ ; } \hfill \\ \gamma_{3}^{{r_{3} }} = \left( {1 - \gamma_{\mathrm{Hi}} } \right)\left( {1 - \gamma_{\mathrm{Avg}} } \right)\left( {1 - \gamma_{\mathrm{Lo}} } \right)/4{ ; }\; \gamma_{4}^{{r_{3} }} = \left( {1 - \gamma_{\mathrm{Hi}} } \right)\left( {1 - \gamma_{\mathrm{Avg}} } \right)\left( {1 - \gamma_{\mathrm{Lo}} } \right)/4. \hfill \\ \end{gathered}$$

The linear weight for the stencil $S_{5}^{{r_{3} }}$ is given by $\, \gamma_{5}^{{r_{3} }} = \left( {1 - \gamma_{\mathrm{Hi}} } \right)\left( {1 - \gamma_{\mathrm{Avg}} } \right)\gamma_{\mathrm{Lo}}$. The linear weight for the stencil $S_{1}^{{r_{5} }}$ is given by $\, \gamma_{1}^{{r_{5} }} = \left( {1 - \gamma_{\mathrm{Hi}} } \right)\gamma_{\mathrm{Avg}}$. The linear weight for the stencil $S_{1}^{{r_{7} }}$ is given by $\, \gamma_{1}^{{r_{7} }} = \gamma_{\mathrm{Hi}}$. Notice that for the linear weights, we have $\gamma_{1}^{{r_{3} }} + \gamma_{2}^{{r_{3} }} + \gamma_{3}^{{r_{3} }} + \gamma_{4}^{{r_{3} }} + \gamma_{5}^{{r_{3} }} + \gamma_{1}^{{r_{5} }} + \gamma_{1}^{{r_{7} }} = 1$. We denote the seventh-order central smoothness indicator by $\beta_{1}^{{r_{7} }}$.

To avoid loss of accuracy at critical points (Borges et al. [41]), we use the smoothness indicators to define

$$\tau = \frac{1}{6}\left( {\left| {\beta_{1}^{{r_{7} }} - \beta_{1}^{{r_{3} }} } \right| + \left| {\beta_{1}^{{r_{7} }} - \beta_{2}^{{r_{3} }} } \right| + \left| {\beta_{1}^{{r_{7} }} - \beta_{3}^{{r_{3} }} } \right| + \left| {\beta_{1}^{{r_{7} }} - \beta_{4}^{{r_{3} }} } \right| + \left| {\beta_{1}^{{r_{7} }} - \beta_{5}^{{r_{3} }} } \right| + \left| {\beta_{1}^{{r_{7} }} - \beta_{1}^{{r_{5} }} } \right|} \right)$$.

The unnormalized non-linear weights are given by

$$\begin{gathered} w_{1}^{{r_{3} }} = \gamma_{1}^{{r_{3} }} \left( {1 + {{\tau^{3} } \mathord{\left/ {\vphantom {{\tau^{3} } {\left( {\beta_{1}^{{r_{3} }} + \varepsilon } \right)^{2} }}} \right. \kern-0pt} {\left( {\beta_{1}^{{r_{3} }} + \varepsilon } \right)^{2} }}} \right){ ; }\;w_{2}^{{r_{3} }} = \gamma_{2}^{{r_{3} }} \left( {1 + {{\tau^{3} } \mathord{\left/ {\vphantom {{\tau^{3} } {\left( {\beta_{2}^{{r_{3} }} + \varepsilon } \right)^{2} }}} \right. \kern-0pt} {\left( {\beta_{2}^{{r_{3} }} + \varepsilon } \right)^{2} }}} \right){ ; } \hfill \\ w_{3}^{{r_{3} }} = \gamma_{3}^{{r_{3} }} \left( {1 + {{\tau^{3} } \mathord{\left/ {\vphantom {{\tau^{3} } {\left( {\beta_{3}^{{r_{3} }} + \varepsilon } \right)^{2} }}} \right. \kern-0pt} {\left( {\beta_{3}^{{r_{3} }} + \varepsilon } \right)^{2} }}} \right){ ; }\;w_{4}^{{r_{3} }} = \gamma_{4}^{{r_{3} }} \left( {1 + {{\tau^{3} } \mathord{\left/ {\vphantom {{\tau^{3} } {\left( {\beta_{4}^{{r_{3} }} + \varepsilon } \right)^{2} }}} \right. \kern-0pt} {\left( {\beta_{4}^{{r_{3} }} + \varepsilon } \right)^{2} }}} \right){ ; } \hfill \\ w_{5}^{{r_{3} }} = \gamma_{5}^{{r_{3} }} \left( {1 + {{\tau^{3} } \mathord{\left/ {\vphantom {{\tau^{3} } {\left( {\beta_{5}^{{r_{3} }} + \varepsilon } \right)^{2} }}} \right. \kern-0pt} {\left( {\beta_{5}^{{r_{3} }} + \varepsilon } \right)^{2} }}} \right){ ; }\;w_{1}^{{r_{5} }} = \gamma_{1}^{{r_{5} }} \left( {1 + {{\tau^{3} } \mathord{\left/ {\vphantom {{\tau^{3} } {\left( {\beta_{1}^{{r_{5} }} + \varepsilon } \right)^{2} }}} \right. \kern-0pt} {\left( {\beta_{1}^{{r_{5} }} + \varepsilon } \right)^{2} }}} \right){ ; } \hfill \\ w_{1}^{{r_{7} }} = \gamma_{1}^{{r_{7} }} \left( {1 + {{\tau^{3} } \mathord{\left/ {\vphantom {{\tau^{3} } {\left( {\beta_{1}^{{r_{7} }} + \varepsilon } \right)^{2} }}} \right. \kern-0pt} {\left( {\beta_{1}^{{r_{7} }} + \varepsilon } \right)^{2} }}} \right){, } \hfill \\ \end{gathered}$$

where $\varepsilon = 10^{ - 12}$ is a small number to avoid the divisions by zero. The normalization of the non-linear weights is given by

$$\begin{gathered} \overline{w}_{1}^{{r_{3} }} = {{w_{1}^{{r_{3} }} } \mathord{\left/ {\vphantom {{w_{1}^{{r_{3} }} } {\left( {w_{1}^{{r_{3} }} + w_{2}^{{r_{3} }} + w_{3}^{{r_{3} }} + w_{4}^{{r_{3} }} + w_{5}^{{r_{3} }} + w_{1}^{{r_{5} }} + w_{1}^{{r_{7} }} } \right)}}} \right. \kern-0pt} {\left( {w_{1}^{{r_{3} }} + w_{2}^{{r_{3} }} + w_{3}^{{r_{3} }} + w_{4}^{{r_{3} }} + w_{5}^{{r_{3} }} + w_{1}^{{r_{5} }} + w_{1}^{{r_{7} }} } \right)}}{ ; }\overline{w}_{2}^{{r_{3} }} = {{w_{2}^{{r_{3} }} } \mathord{\left/ {\vphantom {{w_{2}^{{r_{3} }} } {\left( {w_{1}^{{r_{3} }} + w_{2}^{{r_{3} }} + w_{3}^{{r_{3} }} + w_{4}^{{r_{3} }} + w_{5}^{{r_{3} }} + w_{1}^{{r_{5} }} + w_{1}^{{r_{7} }} } \right)}}} \right. \kern-0pt} {\left( {w_{1}^{{r_{3} }} + w_{2}^{{r_{3} }} + w_{3}^{{r_{3} }} + w_{4}^{{r_{3} }} + w_{5}^{{r_{3} }} + w_{1}^{{r_{5} }} + w_{1}^{{r_{7} }} } \right)}}{ ;} \hfill \\ \overline{w}_{3}^{{r_{3} }} = {{w_{3}^{{r_{3} }} } \mathord{\left/ {\vphantom {{w_{3}^{{r_{3} }} } {\left( {w_{1}^{{r_{3} }} + w_{2}^{{r_{3} }} + w_{3}^{{r_{3} }} + w_{4}^{{r_{3} }} + w_{5}^{{r_{3} }} + w_{1}^{{r_{5} }} + w_{1}^{{r_{7} }} } \right)}}} \right. \kern-0pt} {\left( {w_{1}^{{r_{3} }} + w_{2}^{{r_{3} }} + w_{3}^{{r_{3} }} + w_{4}^{{r_{3} }} + w_{5}^{{r_{3} }} + w_{1}^{{r_{5} }} + w_{1}^{{r_{7} }} } \right)}}{ ; }\overline{w}_{4}^{{r_{3} }} = {{w_{4}^{{r_{3} }} } \mathord{\left/ {\vphantom {{w_{4}^{{r_{3} }} } {\left( {w_{1}^{{r_{3} }} + w_{2}^{{r_{3} }} + w_{3}^{{r_{3} }} + w_{4}^{{r_{3} }} + w_{5}^{{r_{3} }} + w_{1}^{{r_{5} }} + w_{1}^{{r_{7} }} } \right)}}} \right. \kern-0pt} {\left( {w_{1}^{{r_{3} }} + w_{2}^{{r_{3} }} + w_{3}^{{r_{3} }} + w_{4}^{{r_{3} }} + w_{5}^{{r_{3} }} + w_{1}^{{r_{5} }} + w_{1}^{{r_{7} }} } \right)}}{ ;} \hfill \\ \overline{w}_{5}^{{r_{3} }} = {{w_{5}^{{r_{3} }} } \mathord{\left/ {\vphantom {{w_{5}^{{r_{3} }} } {\left( {w_{1}^{{r_{3} }} + w_{2}^{{r_{3} }} + w_{3}^{{r_{3} }} + w_{4}^{{r_{3} }} + w_{5}^{{r_{3} }} + w_{1}^{{r_{5} }} + w_{1}^{{r_{7} }} } \right){ ; }\overline{w}_{1}^{{r_{5} }} = {{w_{1}^{{r_{5} }} } \mathord{\left/ {\vphantom {{w_{1}^{{r_{5} }} } {\left( {w_{1}^{{r_{3} }} + w_{2}^{{r_{3} }} + w_{3}^{{r_{3} }} + w_{4}^{{r_{3} }} + w_{5}^{{r_{3} }} + w_{1}^{{r_{5} }} + w_{1}^{{r_{7} }} } \right)}}} \right. \kern-0pt} {\left( {w_{1}^{{r_{3} }} + w_{2}^{{r_{3} }} + w_{3}^{{r_{3} }} + w_{4}^{{r_{3} }} + w_{5}^{{r_{3} }} + w_{1}^{{r_{5} }} + w_{1}^{{r_{7} }} } \right)}}}}} \right. \kern-0pt} {\left( {w_{1}^{{r_{3} }} + w_{2}^{{r_{3} }} + w_{3}^{{r_{3} }} + w_{4}^{{r_{3} }} + w_{5}^{{r_{3} }} + w_{1}^{{r_{5} }} + w_{1}^{{r_{7} }} } \right){ ; }\overline{w}_{1}^{{r_{5} }} = {{w_{1}^{{r_{5} }} } \mathord{\left/ {\vphantom {{w_{1}^{{r_{5} }} } {\left( {w_{1}^{{r_{3} }} + w_{2}^{{r_{3} }} + w_{3}^{{r_{3} }} + w_{4}^{{r_{3} }} + w_{5}^{{r_{3} }} + w_{1}^{{r_{5} }} + w_{1}^{{r_{7} }} } \right)}}} \right. \kern-0pt} {\left( {w_{1}^{{r_{3} }} + w_{2}^{{r_{3} }} + w_{3}^{{r_{3} }} + w_{4}^{{r_{3} }} + w_{5}^{{r_{3} }} + w_{1}^{{r_{5} }} + w_{1}^{{r_{7} }} } \right)}}}}{ ;} \hfill \\ \overline{w}_{1}^{{r_{7} }} = {{w_{1}^{{r_{7} }} } \mathord{\left/ {\vphantom {{w_{1}^{{r_{7} }} } {\left( {w_{1}^{{r_{3} }} + w_{2}^{{r_{3} }} + w_{3}^{{r_{3} }} + w_{4}^{{r_{3} }} + w_{5}^{{r_{3} }} + w_{1}^{{r_{5} }} + w_{1}^{{r_{7} }} } \right)}}} \right. \kern-0pt} {\left( {w_{1}^{{r_{3} }} + w_{2}^{{r_{3} }} + w_{3}^{{r_{3} }} + w_{4}^{{r_{3} }} + w_{5}^{{r_{3} }} + w_{1}^{{r_{5} }} + w_{1}^{{r_{7} }} } \right)}}. \hfill \\ \end{gathered}$$ Let $P_{i}^{{r_{3} }} {(}x,y{)}$ be the interpolated polynomial corresponding to the stencil $S_{i}^{{r_{3} }}$, $P_{1}^{{r_{5} }} {(}x,y{)}$ be the interpolated polynomial corresponding to the stencil $S_{1}^{{r_{5} }}$, and $P_{1}^{{r_{7} }} {(}x,y{)}$ be the interpolated polynomial corresponding to the stencil $S_{1}^{{r_{7} }}$. We denote the interpolated polynomial for WENO-AO(7,5,3) as $P^{{\mathrm{AO(7,5,3)}}} \left( {x,y} \right)$. Our task in this paragraph is to describe the construction of the order-preserving, non-linearly hybridized, seventh-order polynomial $P^{{\mathrm{AO(7,5,3)}}} \left( {x,y} \right)$. The non-linear weights should be combined in such a way that when all the smoothness indicators seem to have almost similar values, then only the higher order scheme is obtained. The non-linearly hybridized seventh-order accurate interpolation is given by

$$\begin{aligned} P^{{\mathrm{AO(7,5,3)}}} \left( x \right)\;{ = }& \; \frac{{\overline{w}_{1}^{{r_{7} }} }}{{\gamma_{1}^{{r_{7} }} }}\left( \begin{aligned} & P_{1}^{{r_{7} }} \left( {x,y} \right) - \gamma_{1}^{{r_{5} }} P_{1}^{{r_{5} }} \left( {x,y} \right) - \, \gamma_{1}^{{r_{3} }} \, P_{1}^{{r_{3} }} \left( {x,y} \right) - \, \gamma_{2}^{{r_{3} }} \, P_{2}^{{r_{3} }} \left( {x,y} \right) \\& - \gamma_{3}^{{r_{3} }} \, P_{3}^{{r_{3} }} \left( {x,y} \right) - \, \gamma_{4}^{{r_{3} }} \, P_{4}^{{r_{3} }} \left( {x,y} \right) - \, \gamma_{5}^{{r_{3} }} \, P_{5}^{{r_{3} }} \left( {x,y} \right) \end{aligned} \right) \\& + \, \overline{w}_{1}^{{r_{5} }} \, P_{1}^{{r_{5} }} \left( {x,y} \right) \, + \, \overline{w}_{1}^{{r_{3} }} \, P_{1}^{{r_{3} }} \left( {x,y} \right) \, + \, \overline{w}_{2}^{{r_{3} }} \, P_{2}^{{r_{3} }} \left( {x,y} \right) \, + \, \overline{w}_{3}^{{r_{3} }} \, P_{3}^{{r_{3} }} \left( {x,y} \right) \, \\& + \, \overline{w}_{4}^{{r_{3} }} \, P_{4}^{{r_{3} }} \left( {x,y} \right) \, + \, \overline{w}_{5}^{{r_{3} }} \, P_{5}^{{r_{3} }} \left( {x,y} \right). \end{aligned}$$

This completes our description of the seventh-order 2D WENO-AO(7,5,3) interpolation.

### A.4 WENO-AO(9,5,3) Interpolation in Two Dimensions

We will not describe all elements of a ninth-order scheme, because the expressions become much too large. However, we provide the most important ingredients that most readers will find hard to discover on their own. This includes the polynomial in which the interpolation is done and the smoothness indicator expressed as a sum of perfect squares. The 2D WENO-AO(9,5,3) interpolation consists of a non-linear hybridization between a large, centered, ninth-order stencil denoted by $S_{1}^{r9}$, an intermediate central fifth-order stencil denoted by $S_{1}^{r5}$ and the five smaller stencils described in Appendix A.1. The larger central ninth-order accurate stencil is shown in Fig. 6c and the intermediate fifth-order central stencil is shown in Fig. 6a. The ninth-order accurate 2D polynomial is given by

A15 $$\begin{aligned} u(x,y) =& \; u_{00} + u_{x} L_{1} (x) + u_{y} L_{1} (y) + u_{xx} L_{2} (x) + u_{yy} L_{2} (y) + u_{xy} L_{1} (x)L_{1} (y) \\& \, + u_{xxx} L_{3} (x) + \, u_{yyy} L_{3} (y) + u_{xxy} L_{2} (x)L_{1} (y) + \, u_{xyy} L_{1} (x)L_{2} (y) \\& \, + u_{xxxx} L_{4} (x) + u_{yyyy} L_{4} (y) + u_{xxxy} L_{3} (x)L_{1} (y) + u_{xyyy} L_{1} (x)L_{3} (y) + u_{xxyy} L_{2} (x)L_{2} (y) \\& \, + u_{xxxxx} L_{5} (x) + u_{yyyyy} L_{5} (y) + u_{xxxxy} L_{4} (x)L_{1} (y) + u_{xyyyy} L_{1} (x)L_{4} (y) \\& \, + u_{xxxyy} L_{3} (x)L_{2} (y) + u_{xxyyy} L_{2} (x)L_{3} (y) \\& \, + u_{xxxxxx} L_{6} (x) + u_{yyyyyy} L_{6} (y) + u_{xxxxxy} L_{5} (x)L_{1} (y) + u_{xyyyyy} L_{1} (x)L_{5} (y) \\& \, + u_{xxxxyy} L_{4} (x)L_{2} (y) + u_{xxyyyy} L_{2} (x)L_{4} (y) + u_{xxxyyy} L_{3} (x)L_{3} (y) \\& \, + u_{xxxxxxx} L_{7} (x) + u_{yyyyyyy} L_{7} (y) + u_{xxxxxxy} L_{6} (x)L_{1} (y) + u_{xyyyyyy} L_{1} (x)L_{6} (y) \\& \, + u_{xxxxxyy} L_{5} (x)L_{2} (y) + u_{xxyyyyy} L_{2} (x)L_{5} (y) + u_{xxxxyyy} L_{4} (x)L_{3} (y) + u_{xxxyyyy} L_{3} (x)L_{4} (y) \\& \, + u_{xxxxxxxx} L_{8} (x) + u_{yyyyyyyy} L_{8} (y) + u_{xxxxxxxy} L_{7} (x)L_{1} (y) + u_{xyyyyyyy} L_{1} (x)L_{7} (y) \\& \, + u_{xxxxxxyy} L_{6} (x)L_{2} (y) + u_{xxyyyyyy} L_{2} (x)L_{6} (y) + u_{xxxxxyyy} L_{5} (x)L_{3} (y) + u_{xxxyyyyy} L_{3} (x)L_{5} (y) \\& \, + u_{xxxxyyyy} L_{4} (x)L_{4} (y). \end{aligned}$$

For the above ninth-order polynomial, the smoothness indicator expressed as a sum of perfect squares is given by

$$\begin{gathered} \beta^{{r_{9} }} = \left( {u_{x} + \frac{{u_{xxx} }}{10} + \frac{{u_{xxxxx} }}{126} + \frac{{u_{xxxxxxx} }}{{{1\;716}}}} \right)^{2} + \left( {u_{y} + \frac{{u_{yyy} }}{10} + \frac{{u_{yyyyy} }}{126} + \frac{{u_{yyyyyyy} }}{{{1\;716}}}} \right)^{2} \hfill \\ \quad \quad \quad + \frac{13}{3}\left( {u_{xx} + \frac{123}{{455}}u_{xxxx} + \frac{85}{{2\;002}}u_{xxxxxx} + \frac{{{29}}}{{{5\;577}}}u_{xxxxxxxx} } \right)^{2} + \frac{13}{3}\left( {u_{yy} + \frac{123}{{455}}u_{yyyy} + \frac{85}{{2\;002}}u_{yyyyyy} + \frac{{{29}}}{{{5\;577}}}u_{yyyyyyyy} } \right)^{2} \hfill \\ \quad \quad \quad + \frac{7}{6}\left( \begin{gathered} u_{xy} + \frac{13}{{140}}u_{xxxy} + \frac{13}{{140}}u_{xyyy} + \frac{13}{{1\;764}}u_{xxxxxy} + \frac{13}{{1\;764}}u_{xyyyyy} + \frac{3}{350}u_{xxxyyy} \hfill \\ + \frac{3}{7}\left( {\frac{{u_{xxxxxxxy} }}{{{792}}} + \frac{{u_{xyyyyyyy} }}{{{792}}} + \frac{{u_{xxxxxyyy} }}{630} + \frac{{u_{xxxyyyyy} }}{630}} \right) \hfill \\ \end{gathered} \right)^{2} \hfill \\ \quad \quad \quad + \frac{781}{{20}}\left( {u_{xxx} + \frac{26\;045}{{49\;203}}u_{xxxxx} + \frac{{{8\;395}}}{{{60\;918}}}u_{xxxxxxx} } \right)^{2} + \frac{781}{{20}}\left( {u_{yyy} + \frac{26\;045}{{49\;203}}u_{yyyyy} + \frac{{{8\;395}}}{{{60\;918}}}u_{yyyyyyy} } \right)^{2} \hfill \\ \quad \quad \quad + \frac{47}{{10}}\left( {u_{xxy} + \frac{533}{{1\;974}}u_{xxxxy} + \frac{781}{{8\;460}}u_{xxyyy} + \frac{{{10}}}{{{94}}}\left( {\frac{{{1\;105}}}{{{2\;772}}}u_{xxxxxxy} + \frac{{{41}}}{{{175}}}u_{xxxxyyy} + \frac{{{781}}}{{{11\;340}}}u_{xxyyyyy} } \right)} \right)^{2} \hfill \\ \quad \quad \quad + \frac{47}{{10}}\left( {u_{xyy} + \frac{533}{{1\;974}}u_{xyyyy} + \frac{781}{{8\;460}}u_{xxxyy} + \frac{{{10}}}{{{94}}}\left( {\frac{{{1\;105}}}{{{2\;772}}}u_{xyyyyyy} + \frac{{{41}}}{{{175}}}u_{xxxyyyy} + \frac{{{781}}}{{{11\;340}}}u_{xxxxxyy} } \right)} \right)^{2} \hfill \\ \quad \quad \quad + \frac{1\;421\;461}{{2\;275}}\left( {u_{xxxx} + \frac{{{2\;275}}}{{{2\;842\;922}}}\left( {\frac{{{3\;263\;849}}}{{{3\;003}}}u_{xxxxxx} + \frac{{{35\;339\;362}}}{{{83\;655}}}u_{xxxxxxxx} } \right)} \right)^{2} \hfill \\ \quad \quad \quad + \frac{1\;421\;461}{{2\;275}}\left( {u_{yyyy} + \frac{{{2\;275}}}{{{2\;842\;922}}}\left( {\frac{{{3\;263\;849}}}{{{3\;003}}}u_{yyyyyy} + \frac{{{35\;339\;362}}}{{{83\;655}}}u_{yyyyyyyy} } \right)} \right)^{2} \hfill \\ \quad \quad \quad + \frac{88\;841}{{2\;100}}\left( {u_{xxxy} + \frac{2\;100}{{177\;682}}\left( \begin{gathered} \frac{4\;740\;203}{{105\;840}}u_{xxxxxy} + \frac{109\;343}{{14\;000}}u_{xxxyyy} - \frac{1}{105\;840}u_{xyyyyy} \hfill \\ + \frac{{{1\;292\;831}}}{{{110\;880}}}u_{xxxxxxxy} + \frac{{{364\;631}}}{{{88\;200}}}u_{xxxxxyyy} + \frac{{{109\;343}}}{{{176\;400}}}u_{xxxyyyyy} - \frac{1}{{{1\;441\;440}}}u_{xyyyyyyy} \hfill \\ \end{gathered} \right)} \right)^{2} \hfill \\ \quad \quad \quad + \frac{88\;841}{{2\;100}}\left( {u_{xyyy} + \frac{2\;100}{{177\;682}}\left( \begin{gathered} \frac{4\;740\;203}{{105\;840}}u_{xyyyyy} + \frac{109\;343}{{14\;000}}u_{xxxyyy} - \frac{1}{105\;840}u_{xxxxxy} \hfill \\ + \frac{{{1\;292\;831}}}{{{110\;880}}}u_{xyyyyyyy} + \frac{{{364\;631}}}{{{88\;200}}}u_{xxxyyyyy} + \frac{{{109\;343}}}{{{176\;400}}}u_{xxxxxyyy} - \frac{1}{{{1\;441\;440}}}u_{xxxxxxxy} \hfill \\ \end{gathered} \right)} \right)^{2} \hfill \\ \quad \quad \quad + \frac{1}{{{16\;800}}}\left( {u_{xxxy} - u_{xyyy} } \right)^{2} + \frac{{{5\;083}}}{{{270}}}\left( {u_{xxyy} + \frac{{{270}}}{{{10\;166}}}\left( \begin{gathered} \frac{{{32\;021}}}{{{3\;150}}}\left( {u_{xxxxyy} + u_{xxyyyy} } \right) + \frac{{{1\;207}}}{{{756}}}\left( {u_{xxxxxxyy} + u_{xxyyyyyy} } \right) \hfill \\ + \frac{{{3\;362}}}{{{1\;225}}}u_{xxxxyyyy} \hfill \\ \end{gathered} \right)} \right)^{2} \hfill \\ \end{gathered}$$

$$\begin{gathered} \quad \quad \quad + \frac{{{21\;520\;059\;541}}}{{{1\;377\;684}}}\left( {u_{xxxxx} + \frac{{{1\;377\;684}}}{{{43\;040\;119\;082}}}\left( {\frac{{{34\;399\;031\;911}}}{{{852\;852}}}u_{xxxxxxx} } \right)} \right)^{2} \hfill \\ \quad \quad \quad + \frac{{{21\;520\;059\;541}}}{{{1\;377\;684}}}\left( {u_{yyyyy} + \frac{{{1\;377\;684}}}{{{43\;040\;119\;082}}}\left( {\frac{{{34\;399\;031\;911}}}{{{852\;852}}}u_{yyyyyyy} } \right)} \right)^{2} \hfill \\ \quad \quad \quad + \frac{{{2\;805\;965\;789}}}{{{4\;145\;400}}}\left( {u_{xxxxy} + \frac{{{4\;145\;400}}}{{{5\;611\;931\;578}}}\left( \begin{gathered} \frac{{{6\;442\;838\;623}}}{{{5\;471\;928}}}u_{xxxxxxy} + \frac{{{1\;295\;061\;137}}}{{{10\;363\;500}}}u_{xxxxyyy} \hfill \\ - \frac{{{41}}}{{{1\;776\;600}}}u_{xxyyy} - \frac{{{41}}}{{{22\;385\;160}}}u_{xxyyyyy} \hfill \\ \end{gathered} \right)} \right)^{2} \hfill \\ \quad \quad \quad + \frac{{{2\;805\;965\;789}}}{{{4\;145\;400}}}\left( {u_{xyyyy} + \frac{{{4\;145\;400}}}{{{5\;611\;931\;578}}}\left( \begin{gathered} \frac{{{6\;442\;838\;623}}}{{{5\;471\;928}}}u_{xyyyyyy} + \frac{{{1\;295\;061\;137}}}{{{10\;363\;500}}}u_{xxxyyyy} \hfill \\ - \frac{{{41}}}{{{1\;776\;600}}}u_{xxxyy} - \frac{{{41}}}{{{22\;385\;160}}}u_{xxxxxyy} \hfill \\ \end{gathered} \right)} \right)^{2} \hfill \\ \quad \quad \quad + \frac{{{13\;478\;684\;678\;301\;203}}}{{{79\;549\;130\;118\;150}}}\left( {u_{xxxyy} + \frac{{{39\;774\;565\;059\;075}}}{{{13\;478\;684\;678\;301\;203}}}\left( \begin{gathered} \frac{{{102\;738\;105\;636\;515\;023}}}{{{572\;753\;736\;850\;680}}}u_{xxxxxyy} \hfill \\ + \frac{{{33\;964\;215\;589\;171\;433}}}{{{371\;229\;273\;884\;700}}}u_{xxxyyyy} \hfill \\ + \frac{{{2\;302\;712\;395}}}{{{140\;006\;469\;007\;944}}}u_{xyyyyyy} \hfill \\ \end{gathered} \right)} \right)^{2} \hfill \\ \quad \quad \quad + \frac{{{13\;478\;684\;678\;301\;203}}}{{{79\;549\;130\;118\;150}}}\left( {u_{xxyyy} + \frac{{{39\;774\;565\;059\;075}}}{{{13\;478\;684\;678\;301\;203}}}\left( \begin{gathered} \frac{{{102\;738\;105\;636\;515\;023}}}{{{572\;753\;736\;850\;680}}}u_{xxyyyyy} \hfill \\ + \frac{{{33\;964\;215\;589\;171\;433}}}{{{371\;229\;273\;884\;700}}}u_{xxxxyyy} \hfill \\ + \frac{{{2\;302\;712\;395}}}{{{140\;006\;469\;007\;944}}}u_{xxxxxxy} \hfill \\ \end{gathered} \right)} \right)^{2} \hfill \\ \quad \quad \quad + \frac{{{15\;510\;384\;942\;580\;921}}}{{{27\;582\;029\;244}}}\left( {u_{xxxxxx} + \frac{{{5\;423\;630\;339\;859\;998\;294}}}{{{3\;024\;525\;063\;803\;279\;595}}}u_{xxxxxxxx} } \right)^{2} \hfill \\ \quad \quad \quad + \frac{{{15\;510\;384\;942\;580\;921}}}{{{27\;582\;029\;244}}}\left( {u_{yyyyyy} + \frac{{{5\;423\;630\;339\;859\;998\;294}}}{{{3\;024\;525\;063\;803\;279\;595}}}u_{yyyyyyyy} } \right)^{2} \hfill \\ \quad \quad \quad + \frac{{{150\;025\;516\;743\;043}}}{{{8\;865\;621\;072}}}\left( {u_{xxxxxy} + \frac{{{4\;432\;810\;536}}}{{{150\;025\;516\;743\;043}}}\left( \begin{gathered} \frac{{{8\;545\;967\;960\;535\;563}}}{{{195\;580\;973\;952}}}u_{xxxxxxxy} \hfill \\ + \frac{{{1\;206\;359\;027\;303\;963}}}{{{386\;147\;051\;136}}}u_{xxxxxyyy} \hfill \\ + \frac{{{128\;619}}}{{{79\;681\;137\;536}}}u_{xyyyyyyy} \hfill \\ - \frac{{{2\;813}}}{{{358\;206\;912}}}u_{xxxyyy} \hfill \\ - \frac{{{364\;631}}}{{{1\;579\;692\;481\;920}}}u_{xxxyyyyy} \hfill \\ \end{gathered} \right)} \right)^{2} \hfill \\ \end{gathered}$$

$$\begin{gathered} \quad \quad \quad + \frac{{{150\;025\;516\;743\;043}}}{{{8\;865\;621\;072}}}\left( {u_{xyyyyy} + \frac{{{4\;432\;810\;536}}}{{{150\;025\;516\;743\;043}}}\left( \begin{gathered} \frac{{{8\;545\;967\;960\;535\;563}}}{{{195\;580\;973\;952}}}u_{xyyyyyyy} \hfill \\ + \frac{{{1\;206\;359\;027\;303\;963}}}{{{386\;147\;051\;136}}}u_{xxxyyyyy} \hfill \\ + \frac{{{128\;619}}}{{{79\;681\;137\;536}}}u_{xxxxxxxy} \hfill \\ - \frac{{{2\;813}}}{{{358\;206\;912}}}u_{xxxyyy} \hfill \\ - \frac{{{364\;631}}}{{{1\;579\;692\;481\;920}}}u_{xxxxxyyy} \hfill \\ \end{gathered} \right)} \right)^{2} \hfill \\ \quad \quad \quad + \frac{{{1\;461\;613}}}{{{315\;938\;496\;384}}}\left( {u_{xxxxxy} + u_{xyyyyy} } \right)^{2} \hfill \\ \quad \quad \quad + \frac{{{2\;278\;884\;634\;589}}}{{{840\;601\;125}}}\left( {u_{xxxxyy} + \frac{{{840\;601\;125}}}{{{4\;557\;769\;269\;178}}}\left( \begin{gathered} \frac{{{74\;618\;124\;571}}}{{{15\;823\;080}}}u_{xxxxxxyy} + \frac{{{5\;742\;439\;135\;469}}}{{{3\;922\;805\;250}}}u_{xxxxyyyy} \hfill \\ - \frac{{{41}}}{{{58\;017\;960}}}u_{xxyyyyyy} \hfill \\ \end{gathered} \right)} \right)^{2} \hfill \\ \quad \quad \quad + \frac{{{2\;278\;884\;634\;589}}}{{{840\;601\;125}}}\left( {u_{xxyyyy} + \frac{{{840\;601\;125}}}{{{4\;557\;769\;269\;178}}}\left( \begin{gathered} \frac{{{74\;618\;124\;571}}}{{{15\;823\;080}}}u_{xxyyyyyy} + \frac{{{5\;742\;439\;135\;469}}}{{{3\;922\;805\;250}}}u_{xxxxyyyy} \hfill \\ - \frac{{{41}}}{{{58\;017\;960}}}u_{xxxxxxyy} \hfill \\ \end{gathered} \right)} \right)^{2} \hfill \\ \quad \quad \quad + \frac{{{1\;681}}}{{{747\;201\;000}}}\left( {u_{xxxxyy} - u_{xxyyyy} } \right)^{2} \hfill \\ \quad \quad \quad + \frac{{{1\;821\;183\;508\;634\;515\;119\;164\;258\;353}}}{{{1\;194\;226\;157\;193\;994\;011\;404\;800}}}\times \left( \begin{gathered} u_{xxxyyy} + \frac{{{597\;113\;078\;596\;997\;005\;702\;400}}}{{{1\;821\;183\;508\;634\;515\;119\;164\;258\;353}}} \hfill \\ \quad \quad \quad \times \left( \begin{gathered} \frac{{{34\;477\;149\;017\;196\;090\;259}}}{{{4\;346\;983\;212\;186\;138\;201\;513\;472}}}\left( {u_{xxxxxxxy} + u_{xyyyyyyy} } \right) \hfill \\ + \frac{{{850\;244\;495\;026\;926\;242\;498\;309\;371\;309}}}{{{526\;653\;735\;322\;551\;359\;029\;516\;800}}}\left( {u_{xxxxxyyy} + u_{xxxyyyyy} } \right) \hfill \\ \end{gathered} \right) \hfill \\ \end{gathered} \right)^{2} \hfill \\ \end{gathered}$$

$$\begin{gathered} \quad \quad \quad + \frac{{{12\;210\;527\;897\;166\;191\;835\;083}}}{{{443\;141\;066\;068\;272}}}u_{xxxxxxx}^{2} + \frac{{{12\;210\;527\;897\;166\;191\;835\;083}}}{{{443\;141\;066\;068\;272}}}u_{yyyyyyy}^{2} \hfill \\ \quad \quad \quad + \frac{{{729\;093\;562\;208\;721\;865\;238\;938\;774\;441}}}{{{1\;196\;808\;858\;949\;733\;940\;822\;912}}} \times \left( \begin{gathered} u_{xxxxxxy} + \frac{{{598\;404\;429\;474\;866\;970\;411\;456}}}{{{729\;093\;562\;208\;721\;865\;238\;938\;774\;441}}} \hfill \\ \quad \quad \quad \times \left( \begin{gathered} - \frac{{{1\;012\;830\;861\;343\;669}}}{{{213\;502\;365\;304\;291\;055\;520}}}u_{xxxxyyy} \hfill \\ - \frac{{{92\;906\;143\;860\;066\;025}}}{{{12\;553\;939\;079\;892\;314\;064\;576}}}u_{xxyyyyy} \hfill \\ \end{gathered} \right) \hfill \\ \end{gathered} \right)^{2} \hfill \\ \quad \quad \quad + \frac{{{729\;093\;562\;208\;721\;865\;238\;938\;774\;441}}}{{{1\;196\;808\;858\;949\;733\;940\;822\;912}}} \times \left( \begin{gathered} u_{xyyyyyy} + \frac{{{598\;404\;429\;474\;866\;970\;411\;456}}}{{{729\;093\;562\;208\;721\;865\;238\;938\;774\;441}}} \hfill \\ \quad \quad \quad \times \left( \begin{gathered} - \frac{{{1\;012\;830\;861\;343\;669}}}{{{213\;502\;365\;304\;291\;055\;520}}}u_{xxxyyyy} \hfill \\ - \frac{{{92\;906\;143\;860\;066\;025}}}{{{12\;553\;939\;079\;892\;314\;064\;576}}}u_{xxxxxyy} \hfill \\ \end{gathered} \right) \hfill \\ \end{gathered} \right)^{2} \hfill \\ \quad \quad \quad + \frac{{{10\;786\;969\;275\;341\;029\;621\;981\;757\;001\;451\;763\;059\;666}}}{{{159\;157\;479\;162\;352\;939\;572\;334\;139\;766\;598\;095}}} \hfill \\ \quad \quad \quad \times \left( \begin{gathered} u_{xxxxxyy} - \frac{{{303\;518\;589\;617\;885\;469\;324\;811\;578\;066}}}{{{26\;967\;423\;188\;352\;574\;054\;954\;392\;503\;629\;407\;649\;165}}}u_{xxxyyyy} \hfill \\ \quad \quad \quad \times \left( \begin{gathered} - \frac{{{1\;012\;830\;861\;343\;669}}}{{{213\;502\;365\;304\;291\;055\;520}}}u_{xxxyyyy} \hfill \\ - \frac{{{92\;906\;143\;860\;066\;025}}}{{{12\;553\;939\;079\;892\;314\;064\;576}}}u_{xxxxxyy} \hfill \\ \end{gathered} \right) \hfill \\ \end{gathered} \right)^{2} \hfill \\ \quad \quad \quad + \frac{{{10\;786\;969\;275\;341\;029\;621\;981\;757\;001\;451\;763\;059\;666}}}{{{159\;157\;479\;162\;352\;939\;572\;334\;139\;766\;598\;095}}} \hfill \\ \quad \quad \quad \times \left( \begin{gathered} u_{xxyyyyy} - \frac{{{303\;518\;589\;617\;885\;469\;324\;811\;578\;066}}}{{{26\;967\;423\;188\;352\;574\;054\;954\;392\;503\;629\;407\;649\;165}}}u_{xxxxyyy} \hfill \\ \quad \quad \quad \times \left( \begin{gathered} - \frac{{{1\;012\;830\;861\;343\;669}}}{{{213\;502\;365\;304\;291\;055\;520}}}u_{xxxxyyy} \hfill \\ - \frac{{{92\;906\;143\;860\;066\;025}}}{{{12\;553\;939\;079\;892\;314\;064\;576}}}u_{xxyyyyy} \hfill \\ \end{gathered} \right) \hfill \\ \end{gathered} \right)^{2} \hfill \\ \quad \quad \quad + \frac{{{2\;763\;606\;153\;533\;315\;303\;815\;020\;582\;427\;841\;513\;048\;828\;201\;719}}}{{{113\;263\;177\;391\;080\;811\;030\;808\;448\;515\;243\;512\;126\;493\;000}}}\left( {u_{xxxxyyy}^{2} + u_{xxxyyyy}^{2} } \right) \hfill \\ \quad \quad \quad + \frac{{{75\;509\;368\;098\;103\;789\;336\;083\;731\;407\;561}}}{{{42\;818\;201\;328\;263\;029\;226\;415}}}\left( {u_{xxxxxxxx}^{2} + u_{yyyyyyyy}^{2} } \right) \hfill \\ \quad \quad \quad + \frac{{{199\;737\;681\;553\;706\;220\;980\;291\;238\;577\;946\;759\;221\;272\;365}}}{{{100\;349\;412\;338\;258\;061\;005\;083\;687\;187\;257\;696\;748\;484\;264\;781\;248}}}\left( {u_{xxxxxxxy} - u_{xyyyyyyy} } \right)^{2} \hfill \\ \end{gathered}$$

$$\begin{gathered} \quad \quad \quad + \frac{{{12\;479\;128\;819\;356\;707\;044\;610\;023\;473\;398\;906\;994\;677\;171}}}{{{418\;051\;436\;002\;267\;074\;133\;874\;473\;235\;469\;960}}} \hfill \\ \quad \quad \quad \times \left( \begin{gathered} u_{xxxxxxxy} + \frac{{{209\;025\;718\;001\;133\;537\;066\;937\;236\;617\;734\;980}}}{{{12\;479\;128\;819\;356\;707\;044\;610\;023\;473\;398\;906\;994\;677\;171}}}  \hfill \\ \quad \quad \quad \times \left( \begin{gathered} - \frac{{{797\;782\;983\;056\;241\;550\;148\;217\;580\;890\;369\;413\;413\;088\;989}}}{{{147\;366\;269\;867\;371\;977\;699\;773\;246\;918\;350\;463\;756\;515\;353\;874\;560}}}u_{xxxxxyyy} \hfill \\ - \frac{{{206\;096\;134\;225\;029\;155\;035\;475\;769\;629\;598\;824\;081\;807\;593}}}{{{49\;122\;089\;955\;790\;659\;233\;257\;748\;972\;783\;487\;918\;838\;451\;291\;520}}}u_{xxxyyyyy} \hfill \\ \end{gathered} \right) \hfill \\ \end{gathered} \right)^{2} \hfill \\ \quad \quad \quad + \frac{{{12\;479\;128\;819\;356\;707\;044\;610\;023\;473\;398\;906\;994\;677\;171}}}{{{418\;051\;436\;002\;267\;074\;133\;874\;473\;235\;469\;960}}} \hfill \\ \quad \quad \quad \times \left( \begin{gathered} u_{xyyyyyyy} + \frac{{{209\;025\;718\;001\;133\;537\;066\;937\;236\;617\;734\;980}}}{{{12\;479\;128\;819\;356\;707\;044\;610\;023\;473\;398\;906\;994\;677\;171}}}  \hfill \\ \, \times \left( \begin{gathered} - \frac{{{797\;782\;983\;056\;241\;550\;148\;217\;580\;890\;369\;413\;413\;088\;989}}}{{{147\;366\;269\;867\;371\;977\;699\;773\;246\;918\;350\;463\;756\;515\;353\;874\;560}}}u_{xxxyyyyy} \hfill \\ - \frac{{{206\;096\;134\;225\;029\;155\;035\;475\;769\;629\;598\;824\;081\;807\;593}}}{{{49\;122\;089\;955\;790\;659\;233\;257\;748\;972\;783\;487\;918\;838\;451\;291\;520}}}u_{xxxxxyyy} \hfill \\ \end{gathered} \right) \hfill \\ \end{gathered} \right)^{2} \hfill \\ \quad \quad \quad + \frac{{{26\;109\;152\;643\;905}}}{{{46\;695\;768\;186\;740\;593\;536}}}\left( {u_{xxxxxxyy} + u_{xxyyyyyy} } \right)^{2} \hfill \\ \quad \quad \quad + \frac{{{71\;208\;597\;550\;871\;338\;859\;711\;633}}}{{{29\;184\;855\;116\;712\;870\;960}}}\left( {u_{xxxxxxyy} - \frac{{{26\;993\;580\;654\;975}}}{{{142\;417\;195\;101\;742\;677\;719\;423\;266}}}u_{xxxxyyyy} } \right)^{2} \hfill \\ \quad \quad \quad + \frac{{{71\;208\;597\;550\;871\;338\;859\;711\;633}}}{{{29\;184\;855\;116\;712\;870\;960}}}\left( {u_{xxyyyyyy} - \frac{{{26\;993\;580\;654\;975}}}{{{142\;417\;195\;101\;742\;677\;719\;423\;266}}}u_{xxxxyyyy} } \right)^{2} \hfill \\ \quad \quad \quad + \frac{{{7\;444\;465\;066\;772\;591\;584\;581\;493\;491\;226\;457\;438\;274\;714\;717\;234\;883\;183\;513}}}{{{12\;204\;108\;786\;784\;196\;192\;078\;089\;932\;082\;752\;522\;765\;677\;634\;633\;600}}}\left( {u_{xxxxxyyy}^{2} + u_{xxxyyyyy}^{2} } \right)^{2} \hfill \\ \quad \quad \quad{ + }\frac{{{2\;596\;966\;096\;850\;523\;682\;989\;973\;263\;145\;138\;286\;575\;659\;585\;287\;953\;574\;573\;816\;311\;975\;259\;530\;963}}}{{{8\;789\;948\;108\;197\;918\;757\;446\;442\;512\;169\;380\;858\;265\;962\;531\;076\;562\;071\;815\;232\;091\;414\;215\;814\;730\;444\;800}}} \hfill \\ \quad \quad \quad \times \left( {u_{xxxxxyyy} - u_{xxxyyyyy} } \right)^{2} \hfill \\ \quad \quad \quad{ + }\frac{{{111\;753\;476\;905\;238\;355\;219\;800\;438\;823\;702\;300\;677\;230\;800\;235\;409}}}{{{286\;255\;179\;419\;627\;411\;222\;309\;636\;200\;299\;223\;848\;468\;000}}}u_{xxxxyyyy}^{2} . \hfill \\ \end{gathered}$$

## Appendix B A WENO-Stabilized Transcription Strategy from 3D Point Values to Volume Averages

In three dimensions, we non-linearly hybridize between nine smaller third-order stencils and a larger fifth-order stencil. The third-order stencils give rise to interpolating polynomials that have the form

B1 $$\begin{aligned} u(x,y,z) = & \; u_{0} + u_{x} x + u_{y} y + u_{z} z + u_{xx} (x^{2} - 1/12) + u_{yy} (y^{2} - 1/12) + u_{zz} (z^{2} - 1/12) \\& + u_{xy} xy + u_{yz} yz + u_{xz} xz \end{aligned}$$

and a smoothness indicator of the form

B2 $$IS = u_{x}^{2} + 13u_{xx}^{2} /3 + u_{y}^{2} + 13u_{yy}^{2} /3 + u_{z}^{2} + 13u_{zz}^{2} /3 + 7u_{xy}^{2} /6 + 7u_{yz}^{2} /6 + 7u_{xz}^{2} /6 \, {.}$$

The large fifth-order stencil gives rise to an interpolating polynomial of the form

B3 $$\begin{aligned} u(x,y,z) = & \; u_{0} + u_{x} x + u_{y} y + u_{z} z + u_{xx} (x^{2} - 1/12) + u_{yy} (y^{2} - 1/12) + u_{zz} (z^{2} - 1/12) \\& + u_{xy} xy + u_{yz} yz + u_{xz} xz + u_{xxx} (x^{3} - 3x/20) + u_{yyy} (y^{3} - 3y/20) + u_{zzz} (z^{3} - 3z/20) \\& + u_{xxy} (x^{2} - 1/12)y + u_{xxz} (x^{2} - 1/12)z + u_{xyy} x(y^{2} - 1/12) + u_{yyz} (y^{2} - 1/12)z \\& + u_{xzz} x(z^{2} - 1/12) + u_{yzz} y(z^{2} - 1/12) + u_{xyz} xyz \\& + u_{xxxx} \left( {x^{4} - {{3x^{2} } \mathord{\left/ {\vphantom {{3x^{2} } {14}}} \right. \kern-0pt} {14}}\, + \,{3 \mathord{\left/ {\vphantom {3 {560}}} \right. \kern-0pt} {560}}} \right)\,\, + \,u_{yyyy} \left( {y^{4} - {{3y^{2} } \mathord{\left/ {\vphantom {{3y^{2} } {14}}} \right. \kern-0pt} {14}}\, + \,{3 \mathord{\left/ {\vphantom {3 {560}}} \right. \kern-0pt} {560}}} \right) + \,u_{zzzz} \left( {z^{4} - {{3z^{2} } \mathord{\left/ {\vphantom {{3z^{2} } {14}}} \right. \kern-0pt} {14}}\, + \,{3 \mathord{\left/ {\vphantom {3 {560}}} \right. \kern-0pt} {560}}} \right) \\& + u_{xxxy} (x^{3} - 3x/20)y + u_{xxxz} (x^{3} - 3x/20)z + u_{xyyy} x(y^{3} - 3y/20) + u_{yyyz} (y^{3} - 3y/20)z \\& + u_{xzzz} x(z^{3} - 3z/20) + u_{yzzz} y(z^{3} - 3z/20) + u_{xxyy} (x^{2} - 1/12)(y^{2} - 1/12) \\& + u_{yyzz} (y^{2} - 1/12)(z^{2} - 1/12) + u_{xxzz} (x^{2} - 1/12)(z^{2} - 1/12) + u_{xxyz} (x^{2} - 1/12)yz \\& + u_{xyyz} x(y^{2} - 1/12)z + u_{xyzz} xy(z^{2} - 1/12) \end{aligned}$$

and a smoothness indicator of the form

B4 $$\begin{gathered} IS = (u_{x} + u_{xxx} /10)^{2} + 13(u_{xx} + 123u_{xxxx} /455)^{2} /3 + 781u_{xxx}^{2} /20 + 1\;421\;461u_{xxxx}^{2} /2\;275 + (u_{y} + u_{yyy} /10)^{2} \hfill \\ \,\,\,\,\,\,\,\,\,\,\, + 13(u_{yy} + 123u_{yyyy} /455)^{2} /3 + 781u_{yyy}^{2} /20 + 1\;421\;461u_{yyyy}^{2} /2\;275 + (u_{z} + u_{zzz} /10)^{2} \hfill \\ \,\,\,\,\,\,\,\,\,\,\, + 13(u_{zz} + 123u_{zzzz} /455)^{2} /3 + 781u_{zzz}^{2} /20 + 1\;421\;461u_{zzzz}^{2} /2\;275 + 47u_{xxy}^{2} /10 + 47u_{xyy}^{2} /10 \hfill \\ \,\,\,\,\,\,\,\,\,\,\, + 47u_{xxz}^{2} /10 + 47u_{xzz}^{2} /10 + 47u_{yyz}^{2} /10 + 47u_{yzz}^{2} /10 + 61u_{xyz}^{2} /48 \hfill \\ \,\,\,\,\,\,\,\,\,\, + 7(u_{xy} + 13u_{xxxy} /140 + 13u_{xyyy} /140)^{2} /6 + 7(u_{yz} + 13u_{yyyz} /140 + 13u_{yzzz} /140)^{2} /6 \hfill \\ \,\,\,\,\,\,\,\,\,\, + 7(u_{xz} + 13u_{xxxz} /140 + 13u_{xzzz} /140)^{2} /6 + 88\;841(u_{xxxy}^{2} + u_{xyyy}^{2} )/2\;100 + (u_{xxxy} - u_{xyyy} )^{2} /16\;800 \hfill \\ \,\,\,\,\,\,\,\,\,\, + 5\;083u_{xxyy}^{2} /270 + 88\;841(u_{yyyz}^{2} + u_{yzzz}^{2} )/2\;100 + (u_{yyyz} - u_{yzzz} )^{2} /16\;800 + 5\;083u_{yyzz}^{2} /270 \hfill \\ \,\,\,\,\,\,\,\,\,\, + 88\;841(u_{xzzz}^{2} + u_{xxxz}^{2} )/2\;100 + (u_{xzzz} - u_{xxxz} )^{2} /16\;800 + 5\;083u_{xxzz}^{2} /270 + 10\;999u_{xxyz}^{2} /2\;160 \hfill \\ \,\,\,\,\,\,\,\,\,\, + 10\;999u_{xyyz}^{2} /2160 + 10\;999u_{xyzz}^{2} /2\;160 \, {.} \hfill \\ \end{gathered}$$

Please note that this is not an FV-style reconstruction but rather a pointwise interpolation. The eight smaller third-order interpolating stencils are one-sided and they can be identified on the mesh. We shall not describe all of them, but we will provide all the coefficients for one of them below.

Stencil-1 (Biased in 1st Octant, *x*, *y*, *z* > 0):

$$\begin{gathered} u_{0} = (27u_{0,0,0} - 2u_{0,0, + 1} + u_{0,0, + 2} - 2u_{0, + 1,0} + u_{0, + 2,0} - 2u_{ + 1,0,0} + u_{ + 2,0,0} )/24, \hfill \\ u_{x} = ( - 3u_{0,0,0} + 4u_{ + 1,0,0} - u_{ + 2,0,0} )/2, \hfill \\ u_{y} = ( - 3u_{0,0,0} + 4u_{0, + 1,0} - u_{0, + 2,0} )/2, \hfill \\ u_{z} = ( - 3u_{0,0,0} + 4u_{0,0, + 1} - u_{0,0, + 2} )/2, \hfill \\ u_{xx} = (u_{0,0,0} - 2u_{ + 1,0,0} + u_{ + 2,0,0} )/2, \hfill \\ u_{yy} = (u_{0,0,0} - 2u_{0, + 1,0} + u_{0, + 2,0} )/2, \hfill \\ u_{zz} = (u_{0,0,0} - 2u_{0,0, + 1} + u_{0,0, + 2} )/2, \hfill \\ u_{xy} = u_{0,0,0} - u_{0, + 1,0} - u_{ + 1,0,0} + u_{ + 1, + 1,0}, \hfill \\ u_{yz} = u_{0,0,0} - u_{0,0, + 1} - u_{0, + 1,0} + u_{0, + 1, + 1}, \hfill \\ u_{xz} = u_{0,0,0} - u_{0,0, + 1} - u_{ + 1,0,0} + u_{ + 1,0, + 1} . \hfill \\ \end{gathered}$$

These eight one-sided stencils have to be non-linearly hybridized with a smaller third-order central stencil for which we provide all the coefficients below:

$$\begin{gathered} u_{0} = (18u_{0,0,0} + u_{0,0, - 1} + u_{0,0, + 1} + u_{0, - 1,0} + u_{0, + 1,0} + u_{ - 1,0,0} + u_{ + 1,0,0} )/24, \hfill \\ u_{x} = ( - u_{ - 1,0,0} + u_{ + 1,0,0} )/2, \;u_{y} = ( - u_{0, - 1,0} + u_{0, + 1,0} )/2, \;u_{z} = ( - u_{0,0, - 1} + u_{0,0, + 1} )/2, \hfill \\ u_{xx} = ( - 2u_{0,0,0} + u_{ - 1,0,0} + u_{ + 1,0,0} )/2, \hfill \\ u_{yy} = ( - 2u_{0,0,0} + u_{0, - 1,0} + u_{0, + 1,0} )/2, \hfill \\ u_{zz} = ( - 2u_{0,0,0} + u_{0,0, - 1} + u_{0,0, + 1} )/2, \hfill \\ u_{xy} = (u_{ - 1, - 1,0} - u_{ - 1, + 1,0} - u_{ + 1, - 1,0} + u_{ + 1, + 1,0} )/4, \hfill \\ u_{yz} = (u_{0, - 1, - 1} - u_{0, - 1, + 1} - u_{0, + 1, - 1} + u_{0, + 1, + 1} )/4, \hfill \\ u_{xz} = (u_{ - 1,0, - 1} - u_{ - 1,0, + 1} - u_{ + 1,0, - 1} + u_{ + 1,0, + 1} )/4. \hfill \\ \end{gathered}$$

In addition, we will need a larger fifth-order interpolating stencil against which we have to perform non-linear hybridization vis-a-vis the nine smaller third-order stencils. For the large fifth-order stencil, we provide all the coefficients below:

$$\begin{gathered} u_{0} = (4\;134u_{0,0,0} + 268u_{0,0, - 1} - 17u_{0,0, - 2} + 268u_{0,0, + 1} - 17u_{0,0, + 2} + 268u_{0, - 1,0} + 10u_{0, - 1, - 1} + 10u_{0, - 1, + 1} \hfill \\ \quad \quad \; - 17u_{0, - 2,0} + 268u_{0, + 1,0} + 10u_{0, + 1, - 1} + 10u_{0, + 1, + 1} - 17u_{0, + 2,0} + 268u_{ - 1,0,0} + 10u_{ - 1,0, - 1} + 10u_{ - 1,0, + 1} \hfill \\ \quad \quad \; + 10u_{ - 1, - 1,0} + 10u_{ - 1, + 1,0} - 17u_{ - 2,0,0} + 268u_{ + 1,0,0} + 10u_{ + 1,0, - 1} + 10u_{ + 1,0, + 1} + 10u_{ + 1, - 1,0} + 10u_{ + 1, + 1,0} \hfill \\ \quad \quad \; - 17u_{ + 2,0,0} )/5\;760, \hfill \\ u_{x} = ( - 134u_{ - 1,0,0} - 5u_{ - 1,0, - 1} - 5u_{ - 1,0, + 1} - 5u_{ - 1, - 1,0} - 5u_{ - 1, + 1,0} + 17u_{ - 2,0,0} + 134u_{ + 1,0,0} + 5u_{ + 1,0, - 1} \hfill \\ \quad \quad \; + 5u_{ + 1,0, + 1} + 5u_{ + 1, - 1,0} + 5u_{ + 1, + 1,0} - 17u_{ + 2,0,0} )/240, \hfill \\ u_{y} = ( - 134u_{0, - 1,0} - 5u_{0, - 1, - 1} - 5u_{0, - 1, + 1} + 17u_{0, - 2,0} + 134u_{0, + 1,0} + 5u_{0, + 1, - 1} + 5u_{0, + 1, + 1} - 17u_{0, + 2,0} \hfill \\ \quad \quad \; - 5u_{ - 1, - 1,0} + 5u_{ - 1, + 1,0} - 5u_{ + 1, - 1,0} + 5u_{ + 1, + 1,0} )/240, \hfill \\ u_{z} = ( - 134u_{0,0, - 1} + 17u_{0,0, - 2} + 134u_{0,0, + 1} - 17u_{0,0, + 2} - 5u_{0, - 1, - 1} + 5u_{0, - 1, + 1} - 5u_{0, + 1, - 1} + 5u_{0, + 1, + 1} \hfill \\ \quad \quad \; - 5u_{ - 1,0, - 1} + 5u_{ - 1,0, + 1} - 5u_{ + 1,0, - 1} + 5u_{ + 1,0, + 1} )/240, \hfill \\ \end{gathered}$$

$$\begin{gathered} u_{xx} = ( - 346u_{0,0,0} - 14u_{0,0, - 1} - 14u_{0,0, + 1} - 14u_{0, - 1,0} - 14u_{0, + 1,0} + 184u_{ - 1,0,0} + 7u_{ - 1,0, - 1} + 7u_{ - 1,0, + 1} \hfill \\ \quad \quad \; \; + 7u_{ - 1, - 1,0} + 7u_{ - 1, + 1,0} - 11u_{ - 2,0,0} + 184u_{ + 1,0,0} + 7u_{ + 1,0, - 1} + 7u_{ + 1,0, + 1} + 7u_{ + 1, - 1,0} + 7u_{ + 1, + 1,0} \hfill \\ \quad \quad \; \; - 11u_{ + 2,0,0} )/336, \hfill \\ u_{yy} = ( - 346u_{0,0,0} - 14u_{0,0, - 1} - 14u_{0,0, + 1} + 184u_{0, - 1,0} + 7u_{0, - 1, - 1} + 7u_{0, - 1, + 1} - 11u_{0, - 2,0} + 184u_{0, + 1,0} \hfill \\ \quad \quad \; \; + 7u_{0, + 1, - 1} + 7u_{0, + 1, + 1} - 11u_{0, + 2,0} - 14u_{ - 1,0,0} + 7u_{ - 1, - 1,0} + 7u_{ - 1, + 1,0} - 14u_{ + 1,0,0} + 7u_{ + 1, - 1,0} \hfill \\ \quad \quad \; \; + 7u_{ + 1, + 1,0} )/336, \hfill \\ u_{zz} = ( - 346u_{0,0,0} + 184u_{0,0, - 1} - 11u_{0,0, - 2} + 184u_{0,0, + 1} - 11u_{0,0, + 2} - 14u_{0, - 1,0} + 7u_{0, - 1, - 1} + 7u_{0, - 1, + 1} \hfill \\ \quad \quad \; \; - 14u_{0, + 1,0} + 7u_{0, + 1, - 1} + 7u_{0, + 1, + 1} - 14u_{ - 1,0,0} + 7u_{ - 1,0, - 1} + 7u_{ - 1,0, + 1} - 14u_{ + 1,0,0} + 7u_{ + 1,0, - 1} \hfill \\ \quad \quad \; \; + 7u_{ + 1,0, + 1} )/336, \hfill \\ \end{gathered}$$

$$\begin{gathered} u_{xy} = (386u_{ - 1, - 1,0} - 10u_{ - 1, - 1, - 1} + 5u_{ - 1, - 1, - 2} - 10u_{ - 1, - 1, + 1} + 5u_{ - 1, - 1, + 2} - 34u_{ - 1, - 2,0} - 386u_{ - 1, + 1,0} + 10u_{ - 1, + 1, - 1} \hfill \\ \quad \quad \; \; - 5u_{ - 1, + 1, - 2} + 10u_{ - 1, + 1, + 1} - 5u_{ - 1, + 1, + 2} + 34u_{ - 1, + 2,0} - 34u_{ - 2, - 1,0} + 34u_{ - 2, + 1,0} - 386u_{ + 1, - 1,0} + 10u_{ + 1, - 1, - 1} \hfill \\ \quad \quad \; \; - 5u_{ + 1, - 1, - 2} + 10u_{ + 1, - 1, + 1} - 5u_{ + 1, - 1, + 2} + 34u_{ + 1, - 2,0} + 386u_{ + 1, + 1,0} - 10u_{ + 1, + 1, - 1} + 5u_{ + 1, + 1, - 2} - 10u_{ + 1, + 1, + 1} \hfill \\ \quad \quad \; \; + 5u_{ + 1, + 1, + 2} - 34u_{ + 1, + 2,0} + 34u_{ + 2, - 1,0} - 34u_{ + 2, + 1,0} )/960, \hfill \\ u_{yz} = (386u_{0, - 1, - 1} - 34u_{0, - 1, - 2} - 386u_{0, - 1, + 1} + 34u_{0, - 1, + 2} - 34u_{0, - 2, - 1} + 34u_{0, - 2, + 1} - 386u_{0, + 1, - 1} + 34u_{0, + 1, - 2} \hfill \\ \quad \quad \; \; + 386u_{0, + 1, + 1} - 34u_{0, + 1, + 2} + 34u_{0, + 2, - 1} - 34u_{0, + 2, + 1} - 10u_{ - 1, - 1, - 1} + 10u_{ - 1, - 1, + 1} + 10u_{ - 1, + 1, - 1} - 10u_{ - 1, + 1, + 1} \hfill \\ \quad \quad \; \; + 5u_{ - 2, - 1, - 1} - 5u_{ - 2, - 1, + 1} - 5u_{ - 2, + 1, - 1} + 5u_{ - 2, + 1, + 1} - 10u_{ + 1, - 1, - 1} + 10u_{ + 1, - 1, + 1} + 10u_{ + 1, + 1, - 1} - 10u_{ + 1, + 1, + 1} \hfill \\ \quad \quad \; \; + 5u_{ + 2, - 1, - 1} - 5u_{ + 2, - 1, + 1} - 5u_{ + 2, + 1, - 1} + 5u_{ + 2, + 1, + 1} )/960, \hfill \\ u_{xz} = (386u_{ - 1,0, - 1} - 34u_{ - 1,0, - 2} - 386u_{ - 1,0, + 1} + 34u_{ - 1,0, + 2} - 10u_{ - 1, - 1, - 1} + 10u_{ - 1, - 1, + 1} + 5u_{ - 1, - 2, - 1} - 5u_{ - 1, - 2, + 1} \hfill \\ \quad \quad \; \; - 10u_{ - 1, + 1, - 1} + 10u_{ - 1, + 1, + 1} + 5u_{ - 1, + 2, - 1} - 5u_{ - 1, + 2, + 1} - 34u_{ - 2,0, - 1} + 34u_{ - 2,0, + 1} - 386u_{ + 1,0, - 1} + 34u_{ + 1,0, - 2} \hfill \\ \quad \quad \; \; + 386u_{ + 1,0, + 1} - 34u_{ + 1,0, + 2} + 10u_{ + 1, - 1, - 1} - 10u_{ + 1, - 1, + 1} - 5u_{ + 1, - 2, - 1} + 5u_{ + 1, - 2, + 1} + 10u_{ + 1, + 1, - 1} - 10u_{ + 1, + 1, + 1} \hfill \\ \quad \quad \; \; - 5u_{ + 1, + 2, - 1} + 5u_{ + 1, + 2, + 1} + 34u_{ + 2,0, - 1} - 34u_{ + 2,0, + 1} )/960, \hfill \\ \end{gathered}$$

$$\begin{gathered} u_{xxx} = (2u_{ - 1,0,0} - u_{ - 2,0,0} - 2u_{ + 1,0,0} + u_{ + 2,0,0} )/12, \hfill \\ u_{yyy} = (2u_{0, - 1,0} - u_{0, - 2,0} - 2u_{0, + 1,0} + u_{0, + 2,0} )/12, \hfill \\ u_{zzz} = (2u_{0,0, - 1} - u_{0,0, - 2} - 2u_{0,0, + 1} + u_{0,0, + 2} )/12, \hfill \\ u_{xxy} = (2u_{0, - 1,0} - 2u_{0, + 1,0} - u_{ - 1, - 1,0} + u_{ - 1, + 1,0} - u_{ + 1, - 1,0} + u_{ + 1, + 1,0} )/4, \hfill \\ u_{xxz} = (2u_{0,0, - 1} - 2u_{0,0, + 1} - u_{ - 1,0, - 1} + u_{ - 1,0, + 1} - u_{ + 1,0, - 1} + u_{ + 1,0, + 1} )/4, \hfill \\ u_{xyy} = (2u_{ - 1,0,0} - u_{ - 1, - 1,0} - u_{ - 1, + 1,0} - 2u_{ + 1,0,0} + u_{ + 1, - 1,0} + u_{ + 1, + 1,0} )/4, \hfill \\ u_{yyz} = (2u_{0,0, - 1} - 2u_{0,0, + 1} - u_{0, - 1, - 1} + u_{0, - 1, + 1} - u_{0, + 1, - 1} + u_{0, + 1, + 1} )/4, \hfill \\ u_{xzz} = (2u_{ - 1,0,0} - u_{ - 1,0, - 1} - u_{ - 1,0, + 1} - 2u_{ + 1,0,0} + u_{ + 1,0, - 1} + u_{ + 1,0, + 1} )/4, \hfill \\ u_{yzz} = (2u_{0, - 1,0} - u_{0, - 1, - 1} - u_{0, - 1, + 1} - 2u_{0, + 1,0} + u_{0, + 1, - 1} + u_{0, + 1, + 1} )/4, \hfill \\ u_{xyz} = ( - 8u_{ - 1, - 1, - 1} + u_{ - 1, - 1, - 2} + 8u_{ - 1, - 1, + 1} - u_{ - 1, - 1, + 2} + u_{ - 1, - 2, - 1} - u_{ - 1, - 2, + 1} + 8u_{ - 1, + 1, - 1} - u_{ - 1, + 1, - 2} - 8u_{ - 1, + 1, + 1} \hfill \\ \quad \quad \; \; \; + u_{ - 1, + 1, + 2} - u_{ - 1, + 2, - 1} + u_{ - 1, + 2, + 1} + u_{ - 2, - 1, - 1} - u_{ - 2, - 1, + 1} - u_{ - 2, + 1, - 1} + u_{ - 2, + 1, + 1} + 8u_{ + 1, - 1, - 1} - u_{ + 1, - 1, - 2} \hfill \\ \quad \quad \; \; \; - 8u_{ + 1, - 1, + 1} + u_{ + 1, - 1, + 2} - u_{ + 1, - 2, - 1} + u_{ + 1, - 2, + 1} - 8u_{ + 1, + 1, - 1} + u_{ + 1, + 1, - 2} + 8u_{ + 1, + 1, + 1} - u_{ + 1, + 1, + 2} + u_{ + 1, + 2, - 1} \hfill \\ \quad \quad \; \; \; - u_{ + 1, + 2, + 1} - u_{ + 2, - 1, - 1} + u_{ + 2, - 1, + 1} + u_{ + 2, + 1, - 1} - u_{ + 2, + 1, + 1} )/16, \hfill \\ \end{gathered}$$

$$\begin{gathered} u_{xxxx} = (6u_{0,0,0} - 4u_{ - 1,0,0} + u_{ - 2,0,0} - 4u_{ + 1,0,0} + u_{ + 2,0,0} )/24, \hfill \\ u_{yyyy} = (6u_{0,0,0} - 4u_{0, - 1,0} + u_{0, - 2,0} - 4u_{0, + 1,0} + u_{0, + 2,0} )/24, \hfill \\ u_{zzzz} = (6u_{0,0,0} - 4u_{0,0, - 1} + u_{0,0, - 2} - 4u_{0,0, + 1} + u_{0,0, + 2} )/24, \hfill \\ \end{gathered}$$

$$\begin{gathered} u_{xxxy} = ( - 2u_{ - 1, - 1,0} + 2u_{ - 1, + 1,0} + u_{ - 2, - 1,0} - u_{ - 2, + 1,0} + 2u_{ + 1, - 1,0} - 2u_{ + 1, + 1,0} - u_{ + 2, - 1,0} + u_{ + 2, + 1,0} )/24, \hfill \\ u_{xxxz} = ( - 2u_{ - 1,0, - 1} + 2u_{ - 1,0, + 1} + u_{ - 2,0, - 1} - u_{ - 2,0, + 1} + 2u_{ + 1,0, - 1} - 2u_{ + 1,0, + 1} - u_{ + 2,0, - 1} + u_{ + 2,0, + 1} )/24, \hfill \\ u_{xyyy} = ( - 2u_{ - 1, - 1,0} + u_{ - 1, - 2,0} + 2u_{ - 1, + 1,0} - u_{ - 1, + 2,0} + 2u_{ + 1, - 1,0} - u_{ + 1, - 2,0} - 2u_{ + 1, + 1,0} + u_{ + 1, + 2,0} )/24, \hfill \\ u_{yyyz} = ( - 2u_{0, - 1, - 1} + 2u_{0, - 1, + 1} + u_{0, - 2, - 1} - u_{0, - 2, + 1} + 2u_{0, + 1, - 1} - 2u_{0, + 1, + 1} - u_{0, + 2, - 1} + u_{0, + 2, + 1} )/24, \hfill \\ u_{xzzz} = ( - 2u_{ - 1,0, - 1} + u_{ - 1,0, - 2} + 2u_{ - 1,0, + 1} - u_{ - 1,0, + 2} + 2u_{ + 1,0, - 1} - u_{ + 1,0, - 2} - 2u_{ + 1,0, + 1} + u_{ + 1,0, + 2} )/24, \hfill \\ u_{yzzz} = ( - 2u_{0, - 1, - 1} + u_{0, - 1, - 2} + 2u_{0, - 1, + 1} - u_{0, - 1, + 2} + 2u_{0, + 1, - 1} - u_{0, + 1, - 2} - 2u_{0, + 1, + 1} + u_{0, + 1, + 2} )/24, \hfill \\ u_{xxyy} = (4u_{0,0,0} - 2u_{0, - 1,0} - 2u_{0, + 1,0} - 2u_{ - 1,0,0} + u_{ - 1, - 1,0} + u_{ - 1, + 1,0} - 2u_{ + 1,0,0} + u_{ + 1, - 1,0} + u_{ + 1, + 1,0} )/4, \hfill \\ u_{yyzz} = (4u_{0,0,0} - 2u_{0,0, - 1} - 2u_{0,0, + 1} - 2u_{0, - 1,0} + u_{0, - 1, - 1} + u_{0, - 1, + 1} - 2u_{0, + 1,0} + u_{0, + 1, - 1} + u_{0, + 1, + 1} )/4, \hfill \\ u_{xxzz} = (4u_{0,0,0} - 2u_{0,0, - 1} - 2u_{0,0, + 1} - 2u_{ - 1,0,0} + u_{ - 1,0, - 1} + u_{ - 1,0, + 1} - 2u_{ + 1,0,0} + u_{ + 1,0, - 1} + u_{ + 1,0, + 1} )/4, \hfill \\ \end{gathered}$$

$$\begin{gathered} u_{xxyz} = (2u_{0, - 1, - 1} - 2u_{0, - 1, + 1} - 2u_{0, + 1, - 1} + 2u_{0, + 1, + 1} - 2u_{ - 1, - 1, - 1} + 2u_{ - 1, - 1, + 1} + 2u_{ - 1, + 1, - 1} - 2u_{ - 1, + 1, + 1} \hfill \\ \quad \quad \quad \; \; + u_{ - 2, - 1, - 1} - u_{ - 2, - 1, + 1} - u_{ - 2, + 1, - 1} + u_{ - 2, + 1, + 1} - 2u_{ + 1, - 1, - 1} + 2u_{ + 1, - 1, + 1} + 2u_{ + 1, + 1, - 1} - 2u_{ + 1, + 1, + 1} + u_{ + 2, - 1, - 1} \hfill \\ \quad \quad \quad \; \; - u_{ + 2, - 1, + 1} - u_{ + 2, + 1, - 1} + u_{ + 2, + 1, + 1} )/16, \hfill \\ u_{xyyz} = (2u_{ - 1,0, - 1} - 2u_{ - 1,0, + 1} - 2u_{ - 1, - 1, - 1} + 2u_{ - 1, - 1, + 1} + u_{ - 1, - 2, - 1} - u_{ - 1, - 2, + 1} - 2u_{ - 1, + 1, - 1} + 2u_{ - 1, + 1, + 1} \hfill \\ \quad \quad \quad \; \; + u_{ - 1, + 2, - 1} - u_{ - 1, + 2, + 1} - 2u_{ + 1,0, - 1} + 2u_{ + 1,0, + 1} + 2u_{ + 1, - 1, - 1} - 2u_{ + 1, - 1, + 1} - u_{ + 1, - 2, - 1} + u_{ + 1, - 2, + 1} + 2u_{ + 1, + 1, - 1} \hfill \\ \quad \quad \quad \; \; - 2u_{ + 1, + 1, + 1} - u_{ + 1, + 2, - 1} + u_{ + 1, + 2, + 1} )/16, \hfill \\ u_{xyzz} = (2u_{ - 1, - 1,0} - 2u_{ - 1, - 1, - 1} + u_{ - 1, - 1, - 2} - 2u_{ - 1, - 1, + 1} + u_{ - 1, - 1, + 2} - 2u_{ - 1, + 1,0} + 2u_{ - 1, + 1, - 1} - u_{ - 1, + 1, - 2} + 2u_{ - 1, + 1, + 1} \hfill \\ \quad \quad \quad \; \; - u_{ - 1, + 1, + 2} - 2u_{ + 1, - 1,0} + 2u_{ + 1, - 1, - 1} - u_{ + 1, - 1, - 2} + 2u_{ + 1, - 1, + 1} - u_{ + 1, - 1, + 2} + 2u_{ + 1, + 1,0} - 2u_{ + 1, + 1, - 1} + u_{ + 1, + 1, - 2} \hfill \\ \quad \quad \quad \; \; - 2u_{ + 1, + 1, + 1} + u_{ + 1, + 1, + 2} )/16. \hfill \\ \end{gathered}$$

The non-linear WENO hybridization follows the standard WENO-AO approach. This gives us a high-order interpolating polynomial within the zone of interest where the WENO has retained all the higher order modes when the solution is smooth and higher order. If the solution is non-smooth, then the WENO gives us the best lower order polynomial that is non-oscillatory. The variable $u_{0}$ then gives us the volume averaged value in the zone of interest.

## Appendix C A WENO-Stabilized Transcription Strategy from 2D Point Values to Area Averages

In two dimensions, we non-linearly hybridize between five smaller third-order stencils and a larger fifth-order stencil. The third-order stencils give rise to interpolating polynomials that have the form

C1 $$u(x,y) = u_{0} + u_{x} x + u_{y} y + u_{xx} (x^{2} - 1/12) + u_{yy} (y^{2} - 1/12)\, + u_{xy} xy,$$

and a smoothness indicator of the form

C2 $$IS = u_{x}^{2} + 13u_{xx}^{2} /3 + u_{y}^{2} + 13u_{yy}^{2} /3 + 7u_{xy}^{2} /6 \, {.}$$

The large fifth-order stencil gives rise to an interpolating polynomial of the form

C3 $$\begin{gathered} u(x,y) \, = \, u_{0} + u_{x} \, x + u_{y} \, y \, + \, u_{xx} \left( {x^{2} - {1 \mathord{\left/ {\vphantom {1 {12}}} \right. \kern-0pt} {12}}} \right) \, + \, u_{yy} \left( {y^{2} - {1 \mathord{\left/ {\vphantom {1 {12}}} \right. \kern-0pt} {12}}} \right) \, + \, u_{xy} \, x \, y \, \hfill \\ \quad \quad \quad \quad { + }u_{xxx} \left( {x^{3} - {{3x} \mathord{\left/ {\vphantom {{3x} {20}}} \right. \kern-0pt} {20}}} \right){ + }u_{yyy} \left( {y^{3} - {{3y} \mathord{\left/ {\vphantom {{3y} {20}}} \right. \kern-0pt} {20}}} \right) + u_{xxy} \left( {x^{2} - {1 \mathord{\left/ {\vphantom {1 {12}}} \right. \kern-0pt} {12}}} \right)y \, + \, u_{xyy} x\left( {y^{2} - {1 \mathord{\left/ {\vphantom {1 {12}}} \right. \kern-0pt} {12}}} \right) \hfill \\ \quad \quad \quad \quad + \,u_{xxxx} \left( {x^{4} - {{3x^{2} } \mathord{\left/ {\vphantom {{3x^{2} } {14}}} \right. \kern-0pt} {14}}\, + \,{3 \mathord{\left/ {\vphantom {3 {560}}} \right. \kern-0pt} {560}}} \right)\,\, + \,u_{yyyy} \left( {y^{4} - {{3y^{2} } \mathord{\left/ {\vphantom {{3y^{2} } {14}}} \right. \kern-0pt} {14}}\, + \,{3 \mathord{\left/ {\vphantom {3 {560}}} \right. \kern-0pt} {560}}} \right)\, + u_{xxxy} \left( {x^{3} - {{3x} \mathord{\left/ {\vphantom {{3x} {20}}} \right. \kern-0pt} {20}}} \right)y \hfill \\ \quad \quad \quad \quad + \,u_{xyyy} \, x\left( {y^{3} - {{3y} \mathord{\left/ {\vphantom {{3y} {20}}} \right. \kern-0pt} {20}}} \right)\, + \,u_{xxyy} \left( {x^{2} - {1 \mathord{\left/ {\vphantom {1 {12}}} \right. \kern-0pt} {12}}} \right)\left( {y^{2} - {1 \mathord{\left/ {\vphantom {1 {12}}} \right. \kern-0pt} {12}}} \right), \hfill \\ \end{gathered}$$

and a smoothness indicator of the form

C4 $$\begin{gathered} IS = (u_{x} + u_{xxx} /10)^{2} + \frac{13}{3}\,(u_{xx} + 123\,u_{xxxx} /455)^{2} + \frac{781}{{20}}\,u_{xxx}^{2} \hfill \\ \quad \quad \; \; + \frac{1\;421\;461}{{2\;275}}\,u_{xxxx}^{2} + (u_{y} + u_{yyy} /10)^{2} + \frac{13}{3}(u_{yy} + 123\,u_{yyyy} /455)^{2} \hfill \\ \quad \quad \; \; + \frac{781}{{20}}\,u_{yyy}^{2} + \frac{1\;421\;461}{{2\;275}}\,u_{yyyy}^{2} + \frac{7}{6}\,(u_{xy} + 13\,u_{xxxy} /140 + 13\,u_{xyyy} /140)^{2} \hfill \\ \quad \quad \; \; + \frac{47}{{10}}\,u_{xxy}^{2} + \frac{47}{{10}}\,u_{xyy}^{2} + \frac{88\;841}{{2\;100}}\,(u_{xxxy}^{2} + u_{xyyy}^{2} ) + \frac{1}{16\;800}(u_{xxxy} - u_{xyyy} )^{2} + \frac{5\;083}{{270}}\,u_{xxyy}^{2} \, {.} \hfill \\ \end{gathered}$$

Please note that this is not an FV-style reconstruction but rather a pointwise interpolation. The four smaller third-order interpolating stencils are one-sided and they can be identified on the mesh. We shall not describe all of them, but we will provide all the coefficients for one of them below:

Stencil-1 (Biased in 1st Quadrant, *x*, *y* > 0):

$$\begin{gathered} u_{0} = ( - 2u_{0, + 1} + u_{0, + 2} + 26u_{0,0} - 2u_{ + 1,0} + u_{ + 2,0} )/24, \hfill \\ u_{x} = ( - 3u_{0,0} + 4u_{ + 1,0} - u_{ + 2,0} )/2,\;\;u_{y} = (4u_{0, + 1} - u_{0, + 2} - 3u_{0,0} )/2, \hfill \\ u_{xx} = (u_{0,0} - 2u_{ + 1,0} + u_{ + 2,0} )/2,\;\;u_{yy} = ( - 2u_{0, + 1} + u_{0, + 2} + u_{0,0} )/2, \hfill \\ u_{xy} = - u_{0, + 1} + u_{0,0} - u_{ + 1,0} + u_{ + 1, + 1} . \hfill \\ \end{gathered}$$

These four one-sided stencils have to be non-linearly hybridized with a smaller third-order central stencil for which we provide all the coefficients below:

$$\begin{gathered} u_{0} = (20u_{0,0} + u_{0, - 1} + u_{0, + 1} + u_{ - 1,0} + u_{ + 1,0} )/24, \hfill \\ u_{x} = ( - u_{ - 1,0} + u_{ + 1,0} )/2,\;\;u_{y} = ( - u_{0, - 1} + u_{0, + 1} )/2, \hfill \\ u_{xx} = ( - 2u_{0,0} + u_{ - 1,0} + u_{ + 1,0} )/2,\;\;u_{yy} = ( - 2u_{0,0} + u_{0, - 1} + u_{0, + 1} )/2, \hfill \\ u_{xy} = (u_{ - 1, - 1} - u_{ - 1, + 1} - u_{ + 1, - 1} + u_{ + 1, + 1} )/4. \hfill \\ \end{gathered}$$

In addition, we will need a larger fifth-order interpolating stencil against which we have to perform non-linear hybridization vis-a-vis the five smaller third-order stencils. For the large fifth-order stencil, we provide all the coefficients below:

$$\begin{gathered} u_{0} = (4\;636u_{0,0} + 288u_{0, - 1} - 17u_{0, - 2} + 288u_{0, + 1} - 17u_{0, + 2} + 288u_{ - 1,0} + 10u_{ - 1, - 1} + 10u_{ - 1, + 1} - 17u_{ - 2,0} \hfill \\ \quad \quad \; \; + 288u_{ + 1,0} + 10u_{ + 1, - 1} + 10u_{ + 1, + 1} - 17u_{ + 2,0} )/5\;760, \hfill \\ u_{x} = ( - 144u_{ - 1,0} - 5u_{ - 1, - 1} - 5u_{ - 1, + 1} + 17u_{ - 2,0} + 144u_{ + 1,0} + 5u_{ + 1, - 1} + 5u_{ + 1, + 1} - 17u_{ + 2,0} )/240, \hfill \\ u_{y} = ( - 144u_{0, - 1} + 17u_{0, - 2} + 144u_{0, + 1} - 17u_{0, + 2} - 5u_{ - 1, - 1} + 5u_{ - 1, + 1} - 5u_{ + 1, - 1} + 5u_{ + 1, + 1} )/240, \hfill \\ u_{xx} = ( - 374u_{0,0} - 14u_{0, - 1} - 14u_{0, + 1} + 198u_{ - 1,0} + 7u_{ - 1, - 1} + 7u_{ - 1, + 1} - 11u_{ - 2,0} + 198u_{ + 1,0} + 7u_{ + 1, - 1} + 7u_{ + 1, + 1} \hfill \\ \quad \quad \; \; - 11u_{ + 2,0} )/336, \hfill \\ u_{yy} = ( - 374u_{0,0} + 198u_{0, - 1} - 11u_{0, - 2} + 198u_{0, + 1} - 11u_{0, + 2} - 14u_{ - 1,0} + 7u_{ - 1, - 1} + 7u_{ - 1, + 1} - 14u_{ + 1,0} + 7u_{ + 1, - 1} \hfill \\ \quad \quad \; \; + 7u_{ + 1, + 1} )/336, \hfill \\ u_{xy} = (188u_{ - 1, - 1} - 17u_{ - 1, - 2} - 188u_{ - 1, + 1} + 17u_{ - 1, + 2} - 17u_{ - 2, - 1} + 17u_{ - 2, + 1} - 188u_{ + 1, - 1} + 17u_{ + 1, - 2} + 188u_{ + 1, + 1} \hfill \\ \quad \quad \; \; - 17u_{ + 1, + 2} + 17u_{ + 2, - 1} - 17u_{ + 2, + 1} )/480, \hfill \\ u_{xxx} = (2u_{ - 1,0} - u_{ - 2,0} - 2u_{ + 1,0} + u_{ + 2,0} )/12, \hfill \\ u_{yyy} = (2u_{0, - 1} - u_{0, - 2} - 2u_{0, + 1} + u_{0, + 2} )/12, \hfill \\ u_{xxy} = (2u_{0, - 1} - 2u_{0, + 1} - u_{ - 1, - 1} + u_{ - 1, + 1} - u_{ + 1, - 1} + u_{ + 1, + 1} )/4, \hfill \\ u_{xyy} = (2u_{ - 1,0} - u_{ - 1, - 1} - u_{ - 1, + 1} - 2u_{ + 1,0} + u_{ + 1, - 1} + u_{ + 1, + 1} )/4, \hfill \\ u_{xxxx} = (6u_{0,0} - 4u_{ - 1,0} + u_{ - 2,0} - 4u_{ + 1,0} + u_{ + 2,0} )/24, \hfill \\ u_{yyyy} = (6u_{0,0} - 4u_{0, - 1} + u_{0, - 2} - 4u_{0, + 1} + u_{0, + 2} )/24, \hfill \\ u_{xxxy} = ( - 2u_{ - 1, - 1} + 2u_{ - 1, + 1} + u_{ - 2, - 1} - u_{ - 2, + 1} + 2u_{ + 1, - 1} - 2u_{ + 1, + 1} - u_{ + 2, - 1} + u_{ + 2, + 1} )/24, \hfill \\ u_{xyyy} = ( - 2u_{ - 1, - 1} + u_{ - 1, - 2} + 2u_{ - 1, + 1} - u_{ - 1, + 2} + 2u_{ + 1, - 1} - u_{ + 1, - 2} - 2u_{ + 1, + 1} + u_{ + 1, + 2} )/24, \hfill \\ u_{xxyy} = (4u_{0,0} - 2u_{0, - 1} - 2u_{0, + 1} - 2u_{ - 1,0} + u_{ - 1, - 1} + u_{ - 1, + 1} - 2u_{ + 1,0} + u_{ + 1, - 1} + u_{ + 1, + 1} )/4. \hfill \\ \end{gathered}$$

The non-linear WENO hybridization follows the standard WENO-AO approach. This gives us a high-order interpolating polynomial within the zone of interest where the WENO has retained all the higher order modes when the solution is smooth and higher order. If the solution is non-smooth, then the WENO gives us the best lower order polynomial that is non-oscillatory. The variable $u_{0}$ then gives us the volume averaged value in the zone of interest.
